# Supplementary material for: Reovirus infection is regulated by NPC1 and endosomal cholesterol homeostasis
Source: PLoS Pathog. 2022 Mar 9;18(3):e1010322. doi: 10.1371/journal.ppat.1010322 (PMC8906592; doi:10.1371/journal.ppat.1010322)
Supplement: S1 Table — (PDF) [file ppat.1010322.s001.pdf]

Gene Symbol	Number of perturbations	Ranks of perturbations	Most enriched perturbation	STARS Score	Average Score	p-value	FDR	q-value
Nrp1	4	5;6;7;11	4	15.43897608	7.614628772	0	0	0
Slc12a9	4	1;3;18;56	4	12.61179471	7.063286185	0	0	0
St3gal4	4	12;35;44;69	4	12.24915046	6.115368445	0	0	0
Npc1	4	16;19;51;131	4	11.13546164	5.935284028	0	0	0
Ccz1	4	4;8;15;142	4	10.99539344	6.496732638	0	0	0
Cmas	4	41;43;53;150	4	10.90018178	5.654722304	0	0	0
Slc35a1	4	52;89;127;218	4	10.25072084	5.150318119	0	0	0
Lsm12	4	28;54;62;353	4	9.413447997	5.310052377	0	0	0
Ctsl	4	37;60;63;2837	3	8.703672158	4.539347269	0	0	0
Gne	4	36;70;285;581	4	8.547842289	4.672853582	0	0	0
Slc17a5	4	10;67;85;6251	3	8.313557111	4.281249967	0	0	0
Spi1	4	25;33;86;987	3	8.298323993	4.962920443	0	0	0
Nans	4	78;99;600;697	4	8.231615706	4.288797459	0	0	0
Kctd5	4	24;46;100;2701	3	8.10189578	4.51974215	0	0	0
Rasa2	4	125;136;168;759	4	8.083579715	4.494532239	0	0	0
Ctsa	4	434;508;754;850	4	7.886871115	3.72937955	0	0	0
Casp8	4	68;76;122;16249	3	7.842936425	3.893485348	0	0	0
Adam17	4	123;164;212;52051	3	7.123500149	3.482727615	1.18E-06	0.000366487	0.000366487
Tiprl	4	101;116;253;6309	3	6.893370402	3.698191208	1.92E-06	0.000564197	0.000535987
Cbfb	4	110;159;261;3419	3	6.85285421	3.840870637	1.92E-06	0.000535987	0.000535987
Ccnc	4	221;244;453;2819	3	6.135532846	3.630002399	7.68E-06	0.002041856	0.001949044
Neu1	4	38;45;454;9379	3	6.132665378	3.642692089	7.68E-06	0.001949044	0.001949044
Srsf1	4	1558;2073;2394;2483	4	6.02463994	2.709590877	9.45E-06	0.002294527	0.002294527
Rplp2	4	1037;2412;2517;2551	4	5.977704984	2.695361377	1.02E-05	0.002370712	0.002370712
Rps8	4	2167;2324;2337;2751	4	5.846584457	2.634285298	1.31E-05	0.00293556	0.00293556
Rpl27	4	2078;2474;2742;3140	4	5.616828226	2.539960488	2.11E-05	0.004535275	0.004535275
Hist1h3g	4	1770;2366;3097;3237	4	5.563976021	2.518480488	2.33E-05	0.004825411	0.004825411
Dok1	4	109;451;714;10212	3	5.544166596	2.882075753	2.42E-05	0.004829774	0.004829774
Rpl7a	4	1171;1324;2263;3382	4	5.487852405	2.716478911	2.67E-05	0.005146614	0.005002546
Actl6a	4	2325;2493;2963;3385	4	5.486312126	2.483698125	2.69E-05	0.005002546	0.005002546
Rps3	4	1524;2393;2558;3427	4	5.4648904	2.558143574	2.84E-05	0.005107172	0.005047524
Rps6	4	1643;2071;3271;3512	4	5.422328789	2.504619569	2.98E-05	0.005205259	0.005047524
Rps4x	4	2387;2897;3482;3514	4	5.42133979	2.401980056	2.98E-05	0.005047524	0.005047524
Pabpn1	4	1253;2155;2662;3580	4	5.389014712	2.566469332	3.25E-05	0.005335618	0.005335618
Rpl11	4	2080;2083;2576;3610	4	5.374518011	2.536513439	3.40E-05	0.005418771	0.005418771
Rps23	4	1232;2274;3595;3643	4	5.358710133	2.475501082	3.63E-05	0.005634736	0.005510712

Srsf2	4	1572;3169;3679;3717	4	5.323776574	2.386924455	3.93E-05	0.005928173	0.005510712
Fadd	4	26;71;25865;41099	2	5.322380154	2.735636312	3.94E-05	0.005793869	0.005510712
Hspd1	4	1398;2126;2641;3747	4	5.309812046	2.545928732	4.02E-05	0.005751025	0.005510712
Rps5	4	1498;1744;2687;3762	4	5.302871653	2.567978039	4.05E-05	0.005648479	0.005510712
Pold1	4	259;1605;3485;3765	4	5.301486896	2.66330754	4.05E-05	0.005510712	0.005510712
Exosc4	4	1141;2727;3452;3881	4	5.248772247	2.43975223	4.62E-05	0.0061452	0.005997059
Eef2	4	2454;2456;3080;3897	4	5.241625195	2.423061017	4.73E-05	0.006136525	0.005997059
Rrm1	4	1148;2635;3502;3900	4	5.24028839	2.439611395	4.73E-05	0.005997059	0.005997059
Dtymk	4	1827;2693;2999;3982	4	5.204141795	2.430467128	5.10E-05	0.006321899	0.006098571
Rpl37	4	1543;3038;3416;3988	4	5.20152622	2.390058333	5.11E-05	0.006202393	0.006098571
Polr2l	4	1275;2594;3858;3993	4	5.199349577	2.40054314	5.14E-05	0.006105516	0.006098571
Spc24	4	3205;3607;3939;4016	4	5.189372002	2.264689215	5.24E-05	0.006098571	0.006098571
Rps18	4	1140;1556;3768;4123	4	5.143693479	2.491191586	5.75E-05	0.006546279	0.006546279
Eef1a1	4	2808;3328;3878;4176	4	5.121504858	2.278597867	5.98E-05	0.006679224	0.006581206
Aldoa	4	1064;1447;4067;4188	4	5.116520126	2.484316508	6.07E-05	0.00664527	0.006581206
Rnf128	4	9;91;46411;55707	2	5.107032415	2.817337954	6.25E-05	0.006707768	0.006581206
Snrpg	4	1054;2058;2271;4213	4	5.106180982	2.573001917	6.25E-05	0.006581206	0.006581206
Psmb1	4	910;3181;3776;4267	4	5.084056247	2.376962686	6.60E-05	0.006825819	0.006791669
Atxn2l	4	31;94;18529;39415	2	5.07889225	2.62903817	6.69E-05	0.006791669	0.006791669
Rpsa	4	2389;3054;3276;4309	4	5.067040842	2.3378981	6.84E-05	0.006817638	0.006817638
Rps15a	4	2067;3558;3956;4313	4	5.065428986	2.275308673	6.97E-05	0.00682823	0.00682823
Rps2	4	2616;3101;4272;4343	4	5.053387501	2.257471386	7.16E-05	0.006895325	0.006895325
Hmgb1	4	1480;3236;4111;4360	4	5.046600861	2.304503109	7.31E-05	0.006918217	0.006918217
Rpl24	4	1196;1519;2561;4410	4	5.02679246	2.567180401	7.62E-05	0.007091522	0.007028091
Asns	4	1110;3491;4053;4432	4	5.018147811	2.313609698	7.80E-05	0.007137483	0.007028091
Wdr82	4	1368;2066;4008;4498	4	4.992469014	2.381943506	8.11E-05	0.007301661	0.007028091
Slc7a11	4	2653;3199;3529;4501	4	4.991310766	2.287714591	8.11E-05	0.007185761	0.007028091
Rad51	4	1267;2486;2562;4506	4	4.989382069	2.471410958	8.12E-05	0.007086368	0.007028091
Hist2h3b	4	2366;3359;4246;4510	4	4.987840651	2.240506285	8.18E-05	0.007028091	0.007028091
Rpl19	4	1038;1272;1810;4535	4	4.978237653	2.689128197	8.39E-05	0.007096519	0.007096519
Rpl13a	4	1317;2624;4319;4619	4	4.946354969	2.316456305	9.05E-05	0.007544434	0.007544434
Uba52	4	2096;2637;3821;4800	4	4.879581869	2.295867337	0.000105456	0.008658253	0.008658253
Rplp0	4	1261;2496;3160;4824	4	4.870917622	2.393447289	0.000107228	0.008676179	0.008676179
Snapc5	4	2437;2597;4167;4897	4	4.844826402	2.257346255	0.000114318	0.009117671	0.008910213
Rpl32	4	1657;2052;3898;4902	4	4.843053591	2.34465215	0.000114909	0.009035709	0.008910213
Atp6v0c	4	2312;2722;4728;4904	4	4.842344972	2.220853218	0.000114909	0.008910213	0.008910213
Rps11	4	2068;2560;3196;4996	4	4.810057099	2.333840003	0.000123327	0.009432018	0.009432018
Ctu1	4	1887;2884;3268;5124	4	4.766110334	2.306705389	0.000135586	0.010229442	0.010192001

Nutf2	4	3173;3518;3923;5139	4	4.761032347	2.184962957	0.000136916	0.010192001	0.010192001
Rps13	4	1256;3091;3345;5169	4	4.750920689	2.319364569	0.000141494	0.010394244	0.010394244
Rpl34	4	2062;2112;4568;5198	4	4.741201718	2.261214299	0.000145039	0.01051627	0.010487164
Fam3c	4	13;140;49328;67876	2	4.733394314	2.639580122	0.000146516	0.010487164	0.010487164
Mcm2	4	1092;1239;3778;5246	4	4.725233673	2.449269101	0.000149618	0.010573611	0.010560784
Polr2g	4	2003;3229;3651;5296	4	4.708754912	2.2431538	0.000154787	0.010802202	0.010560784
Sf3b3	4	2190;2455;4189;5300	4	4.70744334	2.246421092	0.000154935	0.010679021	0.010560784
Psmd3	4	2797;4706;5135;5315	4	4.702533743	2.066378949	0.000156264	0.010639294	0.010560784
Krr1	4	2088;2122;3500;5323	4	4.699920953	2.319326816	0.000157003	0.010560784	0.010560784
Rpl31	4	1094;1653;4888;5357	4	4.688860233	2.326839967	0.00016099	0.010700109	0.01061303
Ppcdc	4	1260;3342;3839;5369	4	4.684973202	2.257590779	0.000161581	0.01061303	0.01061303
Pabpc1	4	2114;3984;4130;5483	4	4.648473835	2.160436374	0.000178123	0.011563514	0.011563514
Hus1	4	1224;2095;4723;5499	4	4.643411939	2.277008441	0.000181668	0.011658075	0.011658075
Urod	4	23;2179;2771;5548	4	4.628001009	2.745250054	0.000187281	0.011881672	0.011881672
Eif6	4	3555;4073;5389;5600	4	4.61179471	2.041334537	0.000195847	0.012285547	0.012285547
Anapc11	4	3092;3799;4066;5646	4	4.597583323	2.132371215	0.000203232	0.01260715	0.01260715
Nop58	4	1280;2569;3004;5659	4	4.593588042	2.344917528	0.000205595	0.012613593	0.012613593
BC061237	4	1327;2573;2638;5725	4	4.573444854	2.371093033	0.000215491	0.01307701	0.01307701
Vars2	4	1981;2340;4486;5805	4	4.549337922	2.213378577	0.000227602	0.01366346	0.013436567
Eif3e	4	4807;4930;5661;5807	4	4.548739514	1.963136201	0.00022775	0.013526876	0.013436567
Psmc1	4	412;3031;4864;5816	4	4.546049227	2.279875435	0.000228636	0.013436567	0.013436567
Eif3a	4	1557;1956;3803;5920	4	4.515259991	2.298595311	0.000248132	0.014430422	0.014203238
Rpl15	4	1065;2327;5441;5929	4	4.512621017	2.208644349	0.000248575	0.014307158	0.014203238
Rps29	4	2997;3639;4917;5933	4	4.511449425	2.076104505	0.000249314	0.014203238	0.014203238
Hist1h2bp	4	1283;2109;2787;5963	4	4.502687581	2.37907752	0.000253892	0.014317977	0.014317977
Rps14	4	2422;4384;5571;6002	4	4.491362854	2.02622174	0.000262015	0.014628325	0.014548805
Polr2b	4	1311;3447;4489;6010	4	4.48904893	2.169983521	0.000263197	0.014548805	0.014548805
Fdxr	4	1677;2267;6004;6074	4	4.470647668	2.142258042	0.000276342	0.015125669	0.015125669
Snrnp200	4	2230;2343;3361;6097	4	4.464082038	2.26060821	0.000281512	0.01525902	0.01525902
Psmd6	4	2441;4334;4984;6118	4	4.458108928	2.049161065	0.000285056	0.01530259	0.015290357
Rps3a1	4	2290;2378;2679;6133	4	4.453854959	2.312285916	0.000287567	0.015290357	0.015290357
Mre11a	4	1193;5432;5500;6148	4	4.449611383	2.043723069	0.000290373	0.015293914	0.015293914
Nol9	4	2515;3476;4654;6174	4	4.442280318	2.097474716	0.000295395	0.015413001	0.015413001
Alg2	4	1118;2740;5434;6256	4	4.419359858	2.15882555	0.00031238	0.016148329	0.016148329
Rac1	4	4103;5555;6024;6311	4	4.404154097	1.910490386	0.000325082	0.016650778	0.016418286
Rps15	4	1231;2106;4076;6313	4	4.403603661	2.265332988	0.00032523	0.016506904	0.016418286
Rps24	4	1290;2313;3670;6332	4	4.398383193	2.271471643	0.000328922	0.016543912	0.016418286
Dbr1	4	1299;3478;4628;6335	4	4.397560341	2.143174525	0.000329366	0.016418286	0.016418286

Rpl36	4	2035;2272;5850;6387	4	4.383359154	2.11541129	0.000343249	0.016958938	0.016817409
Pfn1	4	1948;4187;5382;6389	4	4.382815266	2.038139845	0.000343397	0.016817409	0.016817409
Cct5	4	2579;5687;6126;6413	4	4.376301858	1.932020435	0.000347828	0.016886283	0.016886283
Plcg2	4	1165;1629;3838;6448	4	4.366846703	2.321502625	0.00035477	0.017074815	0.017074815
Paics	4	2262;5900;6028;6577	4	4.332435451	1.931769283	0.000387706	0.018500541	0.018413696
Rps16	4	2473;2548;3224;6589	4	4.329268788	2.222059667	0.000390217	0.018462554	0.018413696
Ndor1	4	3352;3722;4074;6615	4	4.322427424	2.074091584	0.000396273	0.018591511	0.018413696
Pes1	4	1797;3764;4183;6627	4	4.319278936	2.113747246	0.000398783	0.0185534	0.018413696
Psmc4	4	3110;4663;4757;6629	4	4.318754742	2.002452877	0.000399079	0.018413696	0.018413696
Hsp90b1	4	1628;4828;6587;6704	4	4.299210796	1.961445805	0.000556229	0.025454318	0.025150164
Sys1	4	4345;4588;5831;6705	4	4.298951689	1.924537631	0.000556524	0.025260781	0.025150164
Eif2b2	4	1361;2162;5927;6708	4	4.298174602	2.136119819	0.000558592	0.025150164	0.025150164
Mcl1	4	1033;1227;2037;6764	4	4.283732422	2.526253509	0.000572033	0.025549268	0.025263789
Atp6v1b2	4	2176;4388;5693;6776	4	4.280653229	1.986903225	0.000573657	0.025418485	0.025263789
Vars	4	2584;4038;4039;6778	4	4.28014056	2.07440309	0.000574691	0.025263789	0.025263789
Kat8	4	3062;3916;4494;6812	4	4.271448261	2.037301688	0.000584144	0.025478714	0.025478714
Wdr36	4	2029;3303;3942;6864	4	4.258237719	2.128599913	0.000598766	0.025914034	0.025489165
Nme6	4	2572;3665;5737;6879	4	4.25444558	1.996136273	0.000602015	0.025854242	0.025489165
Alg13	4	1449;3929;5928;6887	4	4.252426486	2.022015638	0.000603788	0.025732417	0.025489165
Med9	4	502;1848;1942;43861	3	4.247323721	2.091261437	0.000609252	0.025768611	0.025489165
Ppp2r4	4	64;247;2738;17039	2	4.241426295	2.634903419	0.000615603	0.02584146	0.025489165
Ddx3x	4	393;2318;5426;6931	4	4.241363223	2.240119929	0.000615603	0.025648614	0.025489165
Nvl	4	1084;1672;1953;38768	3	4.240026156	2.029502031	0.000616342	0.025489165	0.025489165
Fbl	4	1697;2053;3817;7016	4	4.220188497	2.223437525	0.000638349	0.026205161	0.02620047
Snrnp35	4	1862;2268;5819;7027	4	4.217467004	2.091014726	0.000642928	0.02620047	0.02620047
Pprc1	4	4109;6143;6623;7094	4	4.200982082	1.829663187	0.000659174	0.026667898	0.026667898
Nampt	4	2904;4222;6773;7144	4	4.188781035	1.908252224	0.000671876	0.026986224	0.026893595
Sec13	4	1434;3335;4931;7152	4	4.186836795	2.083685438	0.000674387	0.026893595	0.026893595
Rpl17	4	2666;5785;6641;7169	4	4.182712497	1.867554177	0.000679557	0.026907547	0.026907547
Ran	4	1595;1673;2060;9497	3	4.171129792	1.97234372	0.000693145	0.027252302	0.027252302
Gm21637	4	5366;5769;6080;7249	4	4.163434419	1.835021167	0.00070112	0.027373112	0.027373112
Rpl9	4	1093;2397;2764;7275	4	4.157214828	2.303816474	0.000708653	0.027475065	0.027475065
Ikbkg	4	2021;6423;7074;7321	4	4.146265191	1.849540256	0.000726377	0.027968006	0.027915308
Gars	4	1661;4367;7238;7371	4	4.134441173	1.91944448	0.0007441	0.028454194	0.027915308
Psmd14	4	144;2538;4699;7375	4	4.13349872	2.326317165	0.000745134	0.028299895	0.027915308
Cdc5l	4	1083;1133;5475;7383	4	4.131615344	2.251827996	0.00074927	0.028264684	0.027915308
Polr1e	4	3068;5740;7234;7384	4	4.131380066	1.826125293	0.000749417	0.028080522	0.027915308
Strap	4	2818;4096;5061;7387	4	4.130674422	1.977833226	0.000750008	0.027915308	0.027915308

Eif4a3	4	1034;4365;5345;7416	4	4.123867934	2.033248565	0.000757245	0.027998022	0.027998022
Rrp9	4	1705;2347;3579;7454	4	4.114989264	2.195929837	0.000772311	0.028367172	0.02829862
Rps27	4	2016;2501;2648;7475	4	4.11010203	2.248196809	0.000780139	0.02846741	0.02829862
U2af2	4	2403;4257;5400;7477	4	4.109637296	1.963233788	0.000780582	0.02829862	0.02829862
Chek1	4	3247;4747;4867;7511	4	4.10175577	1.947039557	0.000791807	0.028520366	0.028520366
Rps19	4	1115;2157;2188;8835	3	4.09330663	1.935937344	0.00080569	0.028834415	0.02879861
Ppil4	4	3370;5811;6815;7569	4	4.088392797	1.823480454	0.000812927	0.028908114	0.02879861
Naca	4	2497;2908;7470;7584	4	4.084953521	1.93630401	0.000817506	0.028886939	0.02879861
Pelp1	4	2862;3922;6654;7589	4	4.083808607	1.904972405	0.000820165	0.02879861	0.02879861
Rpl29	4	2012;2141;2220;24723	3	4.074569127	1.872478708	0.000834196	0.029108223	0.028953035
Pkmyt1	4	3144;5521;6775;7632	4	4.073993371	1.83555754	0.000834934	0.028953035	0.028953035
Rpl23	4	2264;2356;2539;7676	4	4.064006954	2.25061387	0.000852806	0.029390214	0.029214965
Nol10	4	2131;2154;4373;7678	4	4.063554387	2.13087381	0.000852953	0.029214965	0.029214965
Gins3	4	2151;2181;6496;7747	4	4.048012594	2.024283083	0.00087821	0.029896617	0.029896617
Exoc5	4	160;312;14109;31399	2	4.03922242	2.045599766	0.000892684	0.030205185	0.030001452
Aars2	4	2987;3222;6551;7788	4	4.038843047	1.929187609	0.000893275	0.030043096	0.030001452
Magoh	4	2491;2543;4748;7798	4	4.036613895	2.064169859	0.00089741	0.030001452	0.030001452
Med12	4	504;726;4427;7868	4	4.021089413	2.424813947	0.000929756	0.030897791	0.030836946
Copb1	4	1158;2297;4955;7881	4	4.018221508	2.128801992	0.000933449	0.030836946	0.030836946
Hsd17b10	4	4923;5675;5707;7934	4	4.006578034	1.828275577	0.000955013	0.031363734	0.03122372
Gapdh	4	1400;2210;2342;8276	3	4.005552279	1.885347294	0.000956342	0.03122372	0.03122372
Trmt112	4	1116;1667;2647;7948	4	4.003515384	2.343941408	0.001099018	0.035673346	0.035673346
Spink4	4	2;56330;69044;76008	1	3.998079437	1.999039718	0.001107584	0.035743595	0.035743595
Rpl26	4	1222;2085;2384;59088	3	3.982630234	1.90593365	0.001128409	0.036206379	0.036107861
Sf3b4	4	1057;1531;2390;44706	3	3.979389014	1.985707593	0.001131806	0.036107861	0.036107861
Vmn1r225	4	167;348;29208;55624	2	3.944767381	2.007954174	0.001182467	0.037509727	0.037166686
Rplp1	4	2216;2452;2458;11111	3	3.943219536	1.801434193	0.001185273	0.037386323	0.037166686
Tamm41	4	1590;2238;2459;10177	3	3.942695212	1.853952329	0.001185864	0.037194818	0.037166686
Rpl35a	4	1455;2233;2466;50283	3	3.939030972	1.862578562	0.001191624	0.037166686	0.037166686
Rps17	4	1428;2282;2484;17056	3	3.929656719	1.857580275	0.001204917	0.037372503	0.037372503
Exoc2	4	171;367;9312;77159	2	3.898801895	1.989227657	0.001255429	0.0387241	0.038616305
Ddx10	4	1348;2150;2553;11907	3	3.894347604	1.867247963	0.001261928	0.038710683	0.038616305
Wdr91	4	262;370;3327;34513	2	3.891763446	2.332639079	0.001265768	0.038616305	0.038616305
Sec61a1	4	2182;2203;2581;8263	3	3.880293809	1.809792137	0.001282606	0.038917325	0.038917325
Nsun4	4	1101;1478;2622;27923	3	3.859990889	1.959115492	0.001315985	0.039714304	0.039714304
Gnb2l1	4	1215;2102;2631;11992	3	3.855577159	1.873049316	0.001324847	0.039766785	0.039766785
Commd8	4	104;400;6100;34521	2	3.82437569	2.222022337	0.001522467	0.045454176	0.045454176
Cenpo	4	2124;2585;2721;57870	3	3.812261926	1.76181623	0.001546394	0.045922954	0.045922954

Hist1h2bj	4	1279;2119;2787;10963	3	3.781409445	1.847498227	0.001602076	0.047324804	0.047324804
Det1	4	87;429;3041;43024	2	3.763899026	2.448522333	0.001635751	0.04806524	0.04806524
Nle1	4	1683;2028;2873;10068	3	3.742299723	1.818877773	0.001681685	0.049156256	0.049156256
Tardbp	4	1025;2226;2918;8940	3	3.722305388	1.845307633	0.001722449	0.05008559	0.05008559
Rpl3	4	1973;2189;2935;9878	3	3.714833174	1.77982633	0.001738253	0.050283238	0.050283238
H1foo	4	303;464;59370;75221	2	3.696161522	1.839587325	0.001926272	0.055434923	0.055269045
Cct2	4	197;2652;2983;12370	3	3.693969582	1.977049511	0.001930407	0.055269045	0.055269045
Hspa5	4	1758;2427;3079;8665	3	3.65324449	1.754312086	0.002035568	0.05798253	0.057963236
Dhps	4	1987;2586;3087;23699	3	3.649909051	1.727810848	0.002046054	0.057985391	0.057963236
Atxn1l	4	210;491;33262;65928	2	3.647330745	1.875876062	0.002055655	0.057963236	0.057963236
Gas7	4	422;495;16689;26681	2	3.640327228	1.773671179	0.002072049	0.058131914	0.057978864
Cdc123	4	1542;2968;3118;45749	3	3.637066507	1.721428851	0.002081059	0.058092756	0.057978864
Rpp38	4	2514;2754;3126;55955	3	3.633773323	1.687259356	0.002091841	0.058103217	0.057978864
Bard1	4	1277;1383;3131;31006	3	3.631719422	1.90083982	0.002097749	0.057978864	0.057978864
Nars2	4	455;1539;3157;27740	3	3.621092488	1.98405418	0.002126254	0.058477229	0.058477229
Tuba1c	4	2627;2667;3168;76064	3	3.61662318	1.685400579	0.002140138	0.058570537	0.058570537
Polr1c	4	1687;2252;3201;12385	3	3.603309126	1.761766495	0.002319	0.063155974	0.063155974
Pop5	4	1088;1573;3409;33220	3	3.522468509	1.862707532	0.002727383	0.073917385	0.073300791
Hist1h2bh	4	2109;3153;3411;14472	3	3.521715749	1.648361013	0.002732405	0.073695737	0.073300791
Wdr61	4	1698;2219;3413;9616	3	3.520963437	1.743633354	0.002733144	0.073361253	0.073300791
Ptma	4	1817;3155;3430;15349	3	3.514586806	1.66140275	0.002760615	0.073744092	0.073300791
Eif3f	4	2478;2675;3434;13152	3	3.513091089	1.664599866	0.002766523	0.073549996	0.073300791
Gins4	4	1443;2392;3443;22871	3	3.509732186	1.741613308	0.002779078	0.073533601	0.073300791
Cnga4	4	83;577;16482;25765	2	3.508086377	1.963147847	0.002783656	0.073307322	0.073300791
Pop7	4	1113;1766;3462;16703	3	3.502670369	1.830801618	0.002806402	0.073559342	0.073300791
Fam49b	4	490;2824;3467;10073	3	3.50081854	1.817872892	0.002812457	0.073373591	0.073300791
Tcp1	4	1802;2339;3474;13827	3	3.498230534	1.720831124	0.002822796	0.073300791	0.073300791
Abce1	4	239;871;3547;8458	3	3.471552847	2.137446254	0.002929138	0.075710087	0.075710087
Rpl21	4	2549;2613;3561;9814	3	3.466500402	1.65510224	0.002947896	0.07584379	0.07584379
Copa	4	1244;3473;3572;8562	3	3.462544769	1.667188445	0.002962813	0.07587792	0.07587792
Terf2	4	2690;3354;3608;21616	3	3.449685114	1.593536309	0.00315866	0.080524202	0.079586412
Mau2	4	14;618;22607;24137	2	3.448911591	2.200696441	0.003163239	0.080274376	0.079586412
Rab6a	4	381;3433;3617;36148	3	3.446490584	1.790484266	0.003172544	0.08014621	0.079586412
Gins2	4	1295;1631;3619;8244	3	3.445781787	1.817783863	0.003173725	0.079814906	0.079586412
Mocs3	4	1071;1627;3621;53348	3	3.445073387	1.837818963	0.003178895	0.079586412	0.079586412
Hnrnpa3	4	1796;2633;3638;8191	3	3.439068008	1.681494623	0.003207548	0.079945274	0.079945274
Pcna	4	2242;2719;3649;11106	3	3.435197362	1.651579637	0.003224976	0.080022417	0.080011308
Cnot3	4	2198;2967;3659;13190	3	3.431688878	1.634416436	0.00323886	0.080011308	0.080011308

Rpl38	4	1427;3446;3686;8392	3	3.422264466	1.644596476	0.003287305	0.080850322	0.080676547
Cpsf3l	4	1040;1161;3691;28317	3	3.420526913	1.906727332	0.00329469	0.080676547	0.080676547
Sf3a3	4	1366;3216;3705;48230	3	3.415674473	1.661783491	0.003318174	0.080896784	0.080896784
9130011E15Rik	4	332;647;27572;55058	2	3.409398795	1.730926705	0.003345941	0.081219076	0.081219076
Ssbp1	4	1087;1931;3745;17175	3	3.401912473	1.789135511	0.003525393	0.085204637	0.085204637
Alg1	4	1199;3122;3767;59264	3	3.394406986	1.676104964	0.003563795	0.085761491	0.085695833
Pelo	4	2061;2550;3775;11868	3	3.391688772	1.662539032	0.003576792	0.085704851	0.085695833
Arf4	4	2864;3286;3787;11985	3	3.387622424	1.576207077	0.003596879	0.085817843	0.085695833
Ndufab1	4	2142;3221;3794;9467	3	3.38525644	1.608258889	0.003609581	0.085754428	0.085695833
Mrpl53	4	1808;3099;3818;17587	3	3.377178091	1.631281996	0.003650493	0.086358913	0.085695833
Mphosph10	4	1301;3750;3819;51711	3	3.376842616	1.625263481	0.003654776	0.086095429	0.085695833
Unc119	4	196;672;17967;57638	2	3.376743745	1.795591597	0.003654776	0.085733684	0.085695833
Tceb2	4	2094;2621;3829;9923	3	3.373492774	1.650640604	0.003668512	0.085695833	0.085695833
Xab2	4	2143;3147;3852;15598	3	3.365821815	1.608190581	0.003711345	0.086335154	0.086335154
Arl2	4	2211;3837;3890;11185	3	3.353249652	1.560861921	0.003783569	0.087650058	0.087650058
Rab7	4	1176;3307;3894;15067	3	3.351933548	1.655519063	0.003928312	0.090627139	0.089743482
H2afz	4	2447;3044;3904;14289	3	3.348649301	1.597708568	0.003945593	0.090651216	0.089743482
H2afj	4	1457;2743;3906;38242	3	3.34799348	1.67144524	0.003950319	0.090387838	0.089743482
Nipbl	4	592;698;10216;61050	2	3.34405747	1.626844483	0.003970554	0.090480008	0.089743482
Ftsj3	4	895;2317;3927;17495	3	3.341127932	1.755755273	0.003987244	0.090490981	0.089743482
Cflar	4	1430;1834;3932;11044	3	3.33949879	1.756101407	0.003996696	0.090338281	0.089743482
Oxsr1	4	2449;3114;3945;21301	3	3.335272873	1.589545752	0.004017522	0.090442836	0.089743482
Oraov1	4	1331;1659;3951;16752	3	3.333327233	1.783224597	0.00402727	0.09029818	0.089743482
Ahctf1	4	3239;3251;3958;34850	3	3.331061118	1.552527856	0.004040415	0.090230543	0.089743482
Supt20	4	21;711;44086;68621	2	3.328172161	2.101791058	0.004057548	0.090252147	0.089743482
Hspe1	4	1273;2591;3976;15519	3	3.325252663	1.691602563	0.004078225	0.090352112	0.089743482
Wdr43	4	2689;2942;3981;12070	3	3.323643957	1.589352769	0.004086644	0.090180767	0.089743482
Sbno1	4	1067;3338;3990;18805	3	3.320753467	1.655894741	0.004106288	0.090257501	0.089743482
Cdc37	4	3124;3934;3998;39204	3	3.318189713	1.513286624	0.00412091	0.090223687	0.089743482
3110002H16Rik	4	593;720;19510;50858	2	3.317345416	1.61770163	0.004125636	0.089974326	0.089743482
Plk1	4	2296;2933;4005;18326	3	3.315950712	1.603564762	0.004131692	0.089755781	0.089743482
Sars2	4	3011;3225;4015;11652	3	3.312759048	1.556576543	0.0041472	0.089743482	0.089743482
Supv3l1	4	1384;3204;4040;11714	3	3.304815191	1.633499907	0.004326062	0.093252523	0.092947281
Rpl30	4	3317;3503;4049;8466	3	3.301967661	1.527509503	0.004345115	0.093302986	0.092947281
Obp2b	4	669;735;55151;78144	2	3.299600833	1.594747377	0.004360032	0.0932646	0.092947281
Speer4c	4	1266;3930;4064;29075	3	3.29723608	1.598512261	0.004370962	0.093141529	0.092947281
Vrk3	4	582;738;36845;46873	2	3.296095812	1.613268568	0.004378495	0.092947281	0.092947281
Polr2e	4	2014;3052;4086;70413	3	3.29032853	1.601902798	0.004410102	0.09326363	0.09326363

Dpm2	4	146;771;42301;54515	2	3.25846313	1.798525887	0.004744194	0.099950315	0.099732661
Ppme1	4	734;772;5992;29686	2	3.257348298	1.876349257	0.004751726	0.099732661	0.099732661
Ddx41	4	2144;2273;4245;11608	3	3.241505437	1.645246692	0.004858659	0.101595113	0.101363715
Fam50a	4	2276;3743;4248;76922	3	3.240602278	1.534944627	0.004865749	0.101363715	0.101363715
Snapc4	4	1173;2240;4263;32300	3	3.236096219	1.708403418	0.004897208	0.101639831	0.101639831
Polg2	4	3227;4028;4284;28006	3	3.22981484	1.483251901	0.004939745	0.102142956	0.102142956
Hnrnpk	4	1135;4163;4332;14260	3	3.215574602	1.577574749	0.005176505	0.106643633	0.106643633
Bub3	4	1216;4041;4354;66213	3	3.209101561	1.574916377	0.005229823	0.107345969	0.107345969
Lsm7	4	3162;4308;4372;14372	3	3.203830221	1.464900558	0.005267929	0.107732049	0.107732049
Wbscr16	4	3234;3845;4417;59803	3	3.190748206	1.48285147	0.005501291	0.112093833	0.111822199
Gm10354	4	2193;4064;4447;29075	3	3.18210225	1.507057174	0.0055713	0.113107522	0.111822199
Dnm1l	4	1019;2534;4454;33489	3	3.180093432	1.683092137	0.005583559	0.112945688	0.111822199
Rps26	4	2892;4362;4457;11838	3	3.179233497	1.464745105	0.005590058	0.112668925	0.111822199
Ilf3	4	3477;4443;4459;72321	3	3.178660535	1.44333095	0.005596852	0.112400085	0.111822199
Mcm3	4	2694;4356;4465;11173	3	3.176943223	1.471315075	0.005609258	0.112245482	0.111822199
Actr3	4	2546;3117;4468;11353	3	3.176085451	1.545740469	0.005620188	0.112062535	0.111822199
Dhfr	4	2149;2345;4471;24320	3	3.175228267	1.621875976	0.005628164	0.111822199	0.111822199
Mrps34	4	2970;3657;4484;10277	3	3.171520579	1.496514729	0.005658442	0.112025106	0.112025106
Rhox2a	4	423;4436;4523;8762	3	3.160463047	1.655014237	0.005744106	0.113319241	0.113319241
Snrpb	4	2405;3847;4532;14225	3	3.157925143	1.503179777	0.005906426	0.116111183	0.114730213
Gm13157	4	981;3484;4533;32837	3	3.157643472	1.615004771	0.005908346	0.115741388	0.114730213
Gm17019	4	3683;4264;4544;11289	3	3.154549271	1.440349324	0.005934784	0.115852791	0.114730213
Psmb6	4	2349;2404;4549;58423	3	3.153145345	1.602305342	0.005950144	0.115747931	0.114730213
Bloc1s1	4	2194;4203;4553;8870	3	3.152023339	1.49258905	0.005958859	0.115514956	0.114730213
Tnfrsf1a	4	1078;3141;4555;56225	3	3.151462714	1.625153357	0.005961517	0.115166609	0.114730213
Mars2	4	2410;3851;4560;8852	3	3.150062251	1.500795444	0.005975548	0.115039609	0.114730213
Gtf2h3	4	3280;4390;4573;35816	3	3.146428377	1.443217129	0.006007303	0.115253521	0.114730213
Ikbkap	4	2048;3436;4587;14566	3	3.142526764	1.538634583	0.006046148	0.115601518	0.114730213
Utp18	4	1177;2331;4589;32037	3	3.141970384	1.676127272	0.006049102	0.11526326	0.114730213
Timm10	4	3388;4110;4596;20278	3	3.140025005	1.45206477	0.006065201	0.115176925	0.114730213
Ddx19a	4	3563;3744;4602;48542	3	3.138359946	1.466132089	0.006080118	0.115068814	0.114730213
Tollip	4	368;887;14299;29476	2	3.138003086	1.625755404	0.006082777	0.114730213	0.114730213
Nxf1	4	1456;2505;4625;9224	3	3.131997713	1.636554681	0.006140231	0.115423941	0.115224775
Eif3c	4	3057;3508;4629;20248	3	3.13089454	1.492200575	0.006150275	0.115224775	0.115224775
Triap1	4	1258;1358;4631;9260	3	3.130343318	1.780936242	0.006288963	0.117429024	0.117240012
Nudt21	4	2319;4580;4636;12117	3	3.12896633	1.463769773	0.006303289	0.117304214	0.117240012
Rpl28	4	1995;3947;4651;9161	3	3.124844455	1.508192381	0.006337998	0.117558286	0.117240012
Rrs1	4	2146;4329;4652;15260	3	3.124570146	1.481874561	0.006341838	0.117240012	0.117240012

Cnot1	4	666;909;4812;18747	2	3.116965234	1.921237239	0.00641229	0.118151207	0.1180956
Ccnb1	4	2563;3020;4690;16231	3	3.114190867	1.536205957	0.006440057	0.118272499	0.1180956
Cct8	4	3753;4211;4696;70707	3	3.112559898	1.430671427	0.006451578	0.1180956	0.1180956
Msto1	4	1264;3875;4720;17491	3	3.106057265	1.553783636	0.006510214	0.118779485	0.118623959
Rpl13	4	3677;4637;4725;9142	3	3.104706807	1.410907264	0.00652587	0.118677295	0.118623959
Sacm1l	4	1674;3230;4733;24148	3	3.102549112	1.561806692	0.006544184	0.118623959	0.118623959
Zpr1	4	1666;3347;4735;14558	3	3.102010271	1.554764831	0.006684054	0.120767221	0.120417549
Snrpd3	4	3304;3656;4737;8311	3	3.101471662	1.468910161	0.006686269	0.120417549	0.120417549
Btf3	4	3978;4302;4782;27407	3	3.08941409	1.41515013	0.006805904	0.122178015	0.121244249
Rpl6	4	1082;3806;4784;9583	3	3.088880896	1.56931781	0.006808858	0.121839278	0.121244249
Wdr74	4	4051;4692;4793;20816	3	3.08648434	1.395040941	0.006833376	0.12188734	0.121244249
Atmin	4	34;943;31704;44728	2	3.085444791	1.951199643	0.006845192	0.121709252	0.121244249
Luc7l3	4	1250;4787;4798;30613	3	3.085154909	1.506442949	0.006853758	0.121474704	0.121244249
Rpl37a	4	2507;2601;4803;74667	3	3.083826894	1.562026035	0.006862472	0.121244249	0.121244249
Racgap1	4	1139;3507;4834;12231	3	3.07562465	1.577578082	0.00707368	0.12458156	0.1245243
Dhx33	4	2636;3971;4841;52433	3	3.073779981	1.466628238	0.007092733	0.1245243	0.1245243
Gemin7	4	17;23297;28441;54985	1	3.068905932	1.534452966	0.007145756	0.125061934	0.125061934
Kars	4	3165;4822;4878;18486	3	3.064074719	1.406882424	0.007195678	0.125542095	0.125542095
Snrpa1	4	1195;3700;4895;78781	3	3.059640782	1.557521697	0.007245895	0.126024401	0.125924959
Nup188	4	918;973;20037;39697	2	3.058573669	1.469970969	0.007262733	0.125924959	0.125924959
Cycs	4	3266;4905;4936;9268	3	3.049011704	1.396671631	0.007498458	0.12960957	0.129001437
Rbm22	4	3113;3913;4938;9717	3	3.048495527	1.44730126	0.00750156	0.129262987	0.129001437
Ccdc115	4	3072;3504;4943;16887	3	3.047206021	1.471047141	0.007516034	0.129113902	0.129001437
Paxip1	4	681;4626;4963;11540	3	3.042061323	1.566216926	0.007570091	0.12964362	0.129001437
Psmd4	4	3043;3612;4964;10648	3	3.041804646	1.464319689	0.007571568	0.129272373	0.129001437
Hist1h2ag	4	2396;4141;4966;59280	3	3.04129145	1.459242884	0.007578806	0.129001437	0.129001437
Atic	4	513;3028;4979;25671	3	3.037960829	1.682704653	0.007617502	0.129266004	0.129266004
Bccip	4	3873;4542;5035;63538	3	3.023714646	1.390082376	0.007913488	0.133881832	0.133312905
Wdr83	4	2692;4325;5039;17511	3	3.022703281	1.434288741	0.007925452	0.133679145	0.133312905
Aco2	4	1156;4181;5046;20643	3	3.02093537	1.526126501	0.007944357	0.133594413	0.133312905
Osgep	4	2156;2528;5051;43581	3	3.019674114	1.566861376	0.007963853	0.133520095	0.133312905
Banf1	4	2428;3441;5056;10661	3	3.018414137	1.49042432	0.007975373	0.133312905	0.133312905
Snrpe	4	1614;1655;5069;8632	3	3.015144162	1.684439753	0.008014366	0.133564786	0.133481404
Pold3	4	2269;4238;5075;41961	3	3.013637841	1.452958779	0.008033271	0.133481404	0.133481404
Farsb	4	4057;4100;5094;9178	3	3.008879834	1.403083608	0.008085704	0.133953956	0.133953956
Ldha	4	2075;4450;5103;7997	3	3.006632386	1.450107687	0.008110664	0.13396994	0.13396994
Tfcp2l1	4	20;8649;66178;78821	1	2.998373948	1.499186974	0.008351855	0.137546915	0.137355436
Cct3	4	4684;5033;5144;47064	3	2.996445163	1.345262356	0.008372089	0.137474629	0.137355436

Rpl8	4	1991;4265;5156;13404	3	2.993479279	1.459640447	0.008410343	0.137697783	0.137355436
Cops6	4	2874;4240;5158;44509	3	2.992985653	1.424592913	0.008414035	0.137355436	0.137355436
Smc2	4	2438;2450;5173;23226	3	2.989289702	1.55369259	0.008459969	0.137702648	0.137633147
Atp2a2	4	2906;2914;5181;30768	3	2.987323018	1.499763445	0.008480351	0.137633147	0.137633147
Cse1l	4	1079;5097;5200;13953	3	2.982664597	1.483358325	0.008675903	0.140398745	0.140135961
Tars	4	3259;4347;5202;22171	3	2.982175253	1.404775047	0.008684765	0.140135961	0.140135961
Rpl39	4	2923;3752;5218;11890	3	2.97826743	1.444436468	0.008734982	0.140540072	0.140540072
Rae1	4	1045;1072;20579;21630	2	2.975503546	1.42422425	0.008769396	0.140688322	0.140688322
Samd3	4	527;1081;42845;51601	2	2.96834123	1.518085804	0.008863922	0.141797355	0.14175033
Cad	4	4310;4715;5267;9677	3	2.966375732	1.358407792	0.008886372	0.14175033	0.14175033
Hip1r	4	22;10787;30669;49751	1	2.95701399	1.478506995	0.009146468	0.145483556	0.145086649
Cog1	4	1619;4539;5307;62060	3	2.956752061	1.45862825	0.009147501	0.145086649	0.145086649
Reep5	4	178;1100;72419;77180	2	2.953417344	1.668329766	0.009198752	0.145486219	0.145367393
Rpl18a	4	1551;3530;5337;23826	3	2.949583025	1.512905772	0.00925089	0.145897507	0.145367393
Rps27a	4	1569;4289;5338;22876	3	2.94934477	1.471554161	0.009253253	0.145523693	0.145367393
Exosc3	4	1312;4085;5343;13077	3	2.948154181	1.499603239	0.009269352	0.145367393	0.145367393
Actb	4	1336;4061;5353;8290	3	2.94577643	1.498351033	0.009300368	0.145445257	0.145445257
Dnm2	4	915;2084;5358;31053	3	2.944589264	1.676664644	0.009449838	0.147369965	0.147255767
Kif23	4	1463;2660;5364;39669	3	2.943166165	1.576255049	0.009468891	0.147255767	0.147255767
Nol6	4	2735;5066;5374;10143	3	2.94079796	1.380081364	0.009498283	0.147302541	0.147302541
Rfc5	4	4136;5020;5396;12928	3	2.935603792	1.341712239	0.009571689	0.148029748	0.148029748
Psma3	4	2577;3769;5416;16227	3	2.930900654	1.443874662	0.009643322	0.148725601	0.148518063
Smek2	4	356;1131;15151;28390	2	2.929620403	1.561028047	0.009656467	0.148518063	0.148518063
Lipa	4	378;3532;5445;36035	3	2.924112682	1.654844585	0.009877423	0.151499043	0.151499043
Wee1	4	1678;5381;5454;8476	3	2.922013617	1.411800639	0.00990903	0.151567439	0.151567439
Tubb5	4	1008;3456;5465;22367	3	2.919452918	1.554293458	0.009941819	0.151653484	0.151653484
Snapc3	4	3522;4278;5479;50177	3	2.916201492	1.384254349	0.009992775	0.152015424	0.152015424
Shq1	4	1251;1270;5527;8738	3	2.90511818	1.739510763	0.010289647	0.156106244	0.156106244
Mpi	4	1574;2625;5564;23528	3	2.896642077	1.559924185	0.010426415	0.157752502	0.157260567
Slc20a1	4	1555;1990;5568;12172	3	2.895729213	1.619290357	0.010440889	0.15754455	0.157260567
Hspa8	4	2521;3615;5575;52980	3	2.894133321	1.44545023	0.010463487	0.157459964	0.157260567
Cars	4	4389;4503;5579;30574	3	2.893222307	1.347865456	0.010478404	0.157260567	0.157260567
Ireb2	4	4258;5038;5604;32734	3	2.887543634	1.326342803	0.01069936	0.160146182	0.159964974
Rpl10a	4	4473;4734;5614;21897	3	2.885279452	1.333990662	0.010736875	0.160278003	0.159964974
Ubl5	4	4446;5134;5619;27477	3	2.884148915	1.317797269	0.010750906	0.160059493	0.159964974
Uba1	4	2794;3178;5628;8019	3	2.882116553	1.459170335	0.010786649	0.160164526	0.159964974
Elac2	4	506;1197;10477;18152	2	2.881087805	1.494772765	0.010801862	0.159964974	0.159964974
Pole2	4	801;1200;11795;33666	2	2.878946832	1.429187099	0.01083982	0.160102424	0.160102424

Asna1	4	4906;5285;5662;15087	3	2.874468775	1.300832372	0.011041132	0.162645488	0.160943454
Tsg101	4	1970;3812;5667;11361	3	2.873348086	1.453752781	0.011061071	0.162510422	0.160943454
Mak16	4	1207;5037;5678;13861	3	2.870886144	1.446170012	0.011096223	0.162598987	0.160943454
Vdac2	4	2540;3337;5689;30088	3	2.868429102	1.454872681	0.011133591	0.162719468	0.160943454
Myrf	4	27;7988;62507;70845	1	2.868154729	1.434077365	0.011137135	0.162346285	0.160943454
Sgol1	4	2213;4314;5696;23058	3	2.866868072	1.415103326	0.01115545	0.162189784	0.160943454
Mcmbp	4	2423;3013;5714;64484	3	2.862863036	1.479365781	0.011216597	0.162655219	0.160943454
Orc6	4	2830;4455;5715;21687	3	2.862640915	1.38337431	0.011219108	0.162270149	0.160943454
Ccdc174	4	1660;2174;5723;63563	3	2.860865381	1.585230183	0.011251306	0.162315347	0.160943454
Spc25	4	2320;2809;5731;35812	3	2.859092399	1.497351667	0.011412444	0.164215652	0.160943454
Unc45a	4	186;1230;7504;64949	2	2.85783136	1.852222955	0.011434303	0.164107232	0.160943454
Hist1h3c	4	3097;3596;5745;14481	3	2.855995797	1.417105708	0.011465467	0.162466251	0.160943454
Hist1h3d	4	3097;3596;5745;14481	3	2.855995797	1.417105708	0.011465467	0.162466251	0.160943454
Hist1h3a	4	3596;3758;5745;14481	3	2.855995797	1.393910995	0.011465467	0.162466251	0.160943454
Hist1h3i	4	3097;3596;5745;14481	3	2.855995797	1.417105708	0.011465467	0.162466251	0.160943454
Hist1h3b	4	3097;3596;5745;14481	3	2.855995797	1.417105708	0.011465467	0.162466251	0.160943454
Dynlrb1	4	1370;2270;5746;51122	3	2.855774908	1.594481489	0.011467239	0.162079995	0.160943454
Prc1	4	2672;5341;5753;60991	3	2.854229794	1.350026355	0.011491167	0.162008037	0.160943454
Tnfsf14	4	578;1236;43667;70903	2	2.853671108	1.466770076	0.011502244	0.161755736	0.160943454
Wdr73	4	1103;2406;5760;24872	3	2.852686611	1.603883608	0.011521149	0.161614511	0.160943454
Timeless	4	3410;4817;5768;14611	3	2.850925336	1.346755841	0.011546701	0.161566994	0.160943454
Ctc1	4	2654;5030;5771;14519	3	2.850265505	1.361813275	0.011559698	0.161344487	0.160943454
Speer4d	4	3848;4832;5782;29075	3	2.847849142	1.334111908	0.011602235	0.16153436	0.160943454
Fnta	4	4218;5141;5784;27529	3	2.847410311	1.313066742	0.011606666	0.16119407	0.160943454
Prmt1	4	2564;2695;5787;59049	3	2.846752358	1.493113172	0.011617448	0.160943454	0.160943454
Vprbp	4	1124;5118;5810;50151	3	2.841719679	1.443040862	0.011840914	0.16363323	0.16363323
Hist1h4d	4	5212;5271;5826;33095	3	2.838230762	1.287075151	0.011904572	0.16410673	0.163777206
M6pr	4	29;3231;8214;44081	1	2.837153226	1.626191493	0.011922591	0.163950311	0.163777206
Rpl4	4	3163;3935;5840;15742	3	2.835186039	1.3913024	0.011953017	0.163964847	0.163777206
Gm5795	4	4422;4861;5844;22569	3	2.834317498	1.316894081	0.011968673	0.163777206	0.163777206
Utp11l	4	4393;5539;5857;9185	3	2.831498959	1.290403169	0.012015936	0.164021933	0.164021933
Psmd12	4	2532;5719;5874;9328	3	2.827822879	1.334934687	0.012219906	0.166399359	0.164550004
Eif3g	4	2749;4190;5882;11481	3	2.826096746	1.389600721	0.01224531	0.16633958	0.164550004
Adsl	4	5077;5327;5883;51359	3	2.82588115	1.284115529	0.012252695	0.166035916	0.164550004
Elp6	4	1205;4476;5889;42308	3	2.824588362	1.458781479	0.012275884	0.165947358	0.164550004
Zbtb8os	4	4221;5596;5896;8553	3	2.823081822	1.289835599	0.012299958	0.165871178	0.164550004
Gpn1	4	3599;4541;5898;31143	3	2.822651721	1.3466522	0.012308968	0.165592694	0.164550004
Ryr3	4	30;55922;56088;73979	1	2.822446335	1.411223167	0.012311036	0.165222386	0.164550004

Prelid1	4	2295;4089;5911;9836	3	2.819859715	1.410729923	0.01235697	0.165441155	0.164550004
Ddx54	4	4927;5344;5914;12750	3	2.819216304	1.284408679	0.012367604	0.165187398	0.164550004
Pdcd11	4	1882;2860;5916;19317	3	2.81878755	1.504389683	0.012371739	0.164848261	0.164550004
Ptp4a1	4	4845;5210;5917;15758	3	2.818573229	1.290835892	0.012378829	0.164550004	0.164550004
Rpl41	4	1397;2677;5935;16597	3	2.814721814	1.547545666	0.012585753	0.166903231	0.166069662
Dkc1	4	1044;3024;5939;7995	3	2.813867578	1.552004426	0.012600671	0.166705081	0.166069662
Mrpl57	4	5325;5505;5943;18449	3	2.813013934	1.270189198	0.012615293	0.16650397	0.166069662
Polrmt	4	1274;5476;5946;21691	3	2.812374089	1.409090627	0.012626665	0.166261022	0.166069662
Gart	4	4159;4826;5949;12584	3	2.811734577	1.318236465	0.012641878	0.166069662	0.166069662
Rptor	4	2850;5241;5961;14360	3	2.809179844	1.336328055	0.012688698	0.166293435	0.166292793
Polr2c	4	1527;5631;5971;26158	3	2.807054943	1.383539951	0.012734632	0.166504573	0.166292793
Ifna9	4	830;2961;5975;65982	3	2.806206009	1.578502193	0.012748221	0.166292793	0.166292793
Kif18b	4	1580;3736;5991;33174	3	2.802816117	1.462596147	0.012804198	0.166633653	0.166422558
Upf2	4	1243;1313;6137;24686	2	2.80203204	1.699860068	0.012817786	0.166422558	0.166422558
Capzb	4	1579;5409;5999;10514	3	2.801124666	1.386722406	0.01297405	0.168060608	0.167008967
Uri1	4	129;1316;6665;17182	2	2.800082982	1.914647094	0.012991478	0.167896815	0.167008967
Top2a	4	5809;6001;6007;8285	3	2.799435538	1.242201961	0.013008021	0.167722353	0.167008967
Rnf168	4	4382;5449;6011;16562	3	2.798591842	1.28569469	0.013028107	0.167594295	0.167008967
Epor	4	4091;4638;6013;14195	3	2.798170212	1.324425241	0.013031061	0.167246933	0.167008967
Hnrnpa1	4	4571;5182;6015;13026	3	2.797748725	1.291847859	0.013042434	0.167008967	0.167008967
Ctsm	4	32;36919;56159;73153	1	2.794450343	1.397225171	0.013101513	0.167381574	0.167381574
Cdc20	4	2059;3732;6047;8681	3	2.791024513	1.433124459	0.013172113	0.167899325	0.167460363
Kansl1	4	1330;4328;6053;12116	3	2.789767805	1.446753963	0.013195892	0.167819281	0.167460363
Usp5	4	3310;3472;6054;32980	3	2.789558479	1.401448521	0.013197664	0.167460363	0.167460363
Phb	4	1633;4322;6111;33714	3	2.77768541	1.422996393	0.013568385	0.17177391	0.17177391
Ncbp2	4	4239;4359;6122;42433	3	2.77540726	1.328180402	0.013616091	0.17198787	0.17198787
Rpia	4	2000;2753;6132;25004	3	2.773339874	1.49489734	0.013789784	0.17378863	0.17378863
Psmb4	4	2013;2214;6144;8515	3	2.770863593	1.539426731	0.013834536	0.173959945	0.173959945
Ube2i	4	2408;4290;6155;8984	3	2.768598047	1.383330655	0.013884753	0.174199052	0.173963776
Med20	4	2494;5495;6158;22899	3	2.767980895	1.329474716	0.01389716	0.173963776	0.173963776
Yars2	4	2545;3728;6184;18044	3	2.762645196	1.405290037	0.014132147	0.176509565	0.175756127
Iars	4	2623;4011;6187;8410	3	2.762031028	1.387111224	0.014144406	0.176268341	0.175756127
Ppwd1	4	3911;6096;6188;47723	3	2.761826374	1.264355215	0.014150018	0.175945549	0.175756127
Lsm3	4	1157;3840;6193;12234	3	2.760803612	1.478499235	0.014166265	0.175756127	0.175756127
Scgb1b3	4	966;5120;6208;8821	3	2.757740431	1.437759544	0.014230366	0.176159937	0.176159937
Rangap1	4	5009;6092;6221;32299	3	2.755091843	1.240693658	0.014295648	0.176576555	0.176219527
Polr3b	4	1129;3804;6222;12075	3	2.754888342	1.481506342	0.014298307	0.176219527	0.176219527
Taf1c	4	2223;5175;6241;11521	3	2.751028219	1.348677137	0.014383085	0.176873928	0.176873928

Chordc1	4	2407;3357;6252;8124	3	2.748798949	1.429002518	0.014555743	0.17860377	0.17860377
Pdrg1	4	2261;3941;6266;22382	3	2.745967541	1.401314642	0.014622946	0.179034883	0.179034883
Taf8	4	3369;4511;6276;42947	3	2.7439491	1.334515662	0.014660018	0.179096018	0.179096018
Hps3	4	648;5523;6296;28675	3	2.739922156	1.460066563	0.014753067	0.179839247	0.179839247
Lsm4	4	2778;5252;6306;16490	3	2.737913631	1.320561229	0.014938723	0.181705647	0.18020516
Hist1h2ae	4	1245;1416;8335;11610	2	2.737577917	1.320671227	0.014941973	0.181350072	0.18020516
Eif3d	4	3417;4982;6310;11733	3	2.737111142	1.311275392	0.014956595	0.18113377	0.18020516
Gmppb	4	3001;3095;6314;9865	3	2.736309177	1.421348195	0.014971069	0.18091662	0.18020516
Ikbkb	4	2281;4014;6320;27226	3	2.735107212	1.393951614	0.014996621	0.180833981	0.18020516
Pagr1a	4	5117;5294;6321;41194	3	2.734907	1.261947684	0.015004153	0.180534887	0.18020516
Umps	4	3189;5119;6323;11056	3	2.734506672	1.311657183	0.015009027	0.18020516	0.18020516
Txnl4a	4	2487;2488;6338;44549	3	2.731508373	1.484058314	0.015068254	0.18052803	0.180503789
Eif5	4	2147;4593;6348;13556	3	2.729513574	1.370972138	0.01511788	0.180734746	0.180503789
Pgs1	4	3306;4969;6353;9010	3	2.728517391	1.312768061	0.015136343	0.180568805	0.180503789
Tmem192	4	555;1433;46080;55810	2	2.727400827	1.430431947	0.015163224	0.180503789	0.180503789
Cdipt	4	3692;3814;6368;14800	3	2.72553369	1.355794772	0.015336473	0.182177718	0.182177718
Rpl23a	4	2805;4202;6378;10317	3	2.723548584	1.361424982	0.015380191	0.182309146	0.182309146
Nubp1	4	4780;5350;6405;19744	3	2.718204809	1.261578115	0.015500417	0.183344976	0.182455623
Ddx55	4	3146;3589;6409;17388	3	2.717415117	1.381367863	0.015526707	0.183267667	0.182455623
Sbds	4	1024;4193;6410;32257	3	2.717217774	1.462226405	0.015529514	0.18291408	0.182455623
Oip5	4	1429;4445;6415;38547	3	2.716231533	1.415532129	0.015553145	0.182806756	0.182455623
Prpf38a	4	4134;5757;6416;30064	3	2.716034381	1.25927285	0.015555951	0.182455623	0.182455623
Nars	4	2129;3149;6420;11771	3	2.715246087	1.446067159	0.015701433	0.183775898	0.183505286
Rps28	4	2065;3002;6421;32641	3	2.715049093	1.459027062	0.015711182	0.183505286	0.183505286
Wdr18	4	1586;2965;6432;28332	3	2.712884245	1.487723245	0.015756968	0.183655848	0.183642582
Ppard	4	1265;1460;35291;49756	2	2.711487414	1.309778143	0.015788723	0.183642582	0.183642582
Mrc1	4	39;40285;45421;67343	1	2.708650281	1.354325141	0.01585563	0.184037382	0.184037382
Lilra5	4	898;1472;54563;56976	2	2.704510835	1.355028485	0.016081164	0.186267922	0.185598077
Ppat	4	1829;6455;6480;13678	3	2.703482176	1.312090654	0.01611026	0.1862186	0.185598077
Hist1h3e	4	1537;3596;6485;14481	3	2.702506934	1.448228734	0.016134926	0.18611837	0.185598077
Nat10	4	4871;5775;6487;9492	3	2.702117055	1.240551509	0.016140834	0.185802629	0.185598077
Zfp780b	4	664;1479;14458;31519	2	2.700467893	1.396094914	0.016178201	0.185849584	0.185598077
Rsl24d1	4	1152;2570;6501;8086	3	2.699391373	1.547186666	0.016203753	0.185760888	0.185598077
Fam175a	4	40;5096;64640;77808	1	2.697671265	1.454923652	0.016245551	0.185858429	0.185598077
Yae1d1	4	2822;4805;6512;18752	3	2.697254016	1.326891384	0.016256038	0.185598077	0.185598077
Plrg1	4	4537;6109;6527;61505	3	2.694345444	1.233644212	0.016326933	0.186027072	0.186027072
Mrpl32	4	1726;5329;6556;15936	3	2.688741741	1.352577695	0.016608592	0.188850853	0.188759082
Fam96b	4	3924;6503;6563;15898	3	2.687392961	1.232613123	0.01663887	0.188810592	0.188759082

Ipo11	4	3429;3996;6571;21909	3	2.685853318	1.343171684	0.016682588	0.188922701	0.188759082
C8a	4	1349;1506;16296;36569	2	2.685054391	1.292125702	0.016707697	0.188824034	0.188759082
Nfyc	4	2516;4418;6581;67785	3	2.683931487	1.351841248	0.01673576	0.188759082	0.188759082
Eif2b1	4	1077;2022;6592;12834	3	2.681820956	1.600329624	0.016915508	0.190401771	0.18913955
Pafah1b1	4	305;3007;6594;12365	3	2.681437614	1.650624856	0.016927914	0.190158037	0.18913955
Tubgcp3	4	1119;5041;6599;14167	3	2.680479785	1.406261746	0.016953761	0.190065961	0.18913955
Atp5f1	4	1411;1782;6600;11817	3	2.68028831	1.598772664	0.016956124	0.189711508	0.18913955
Cdan1	4	1048;3104;6608;15441	3	2.678757583	1.5123852	0.016992606	0.189739435	0.18913955
Pgd	4	2008;4234;6609;15527	3	2.678566377	1.381555231	0.01699615	0.189400215	0.18913955
Gpn2	4	1581;2659;6611;9425	3	2.678184055	1.502158471	0.017006637	0.18913955	0.18913955
Zfp959	4	42;26966;37422;41293	1	2.676514701	1.338257351	0.017050651	0.189252055	0.189252055
Coasy	4	2304;3581;6630;11878	3	2.674557945	1.401368654	0.017104708	0.189475368	0.189475368
Olfr206	4	338;1530;60762;79116	2	2.671588311	1.482429862	0.017307497	0.191342089	0.191342089
Traip	4	1329;1545;7147;21345	2	2.66328102	1.610142834	0.017657983	0.194831068	0.194555756
Exosc7	4	251;1546;11970;30558	2	2.66273013	1.522081943	0.017667879	0.194555756	0.194555756
Rad9a	4	1431;3464;6709;8000	3	2.659595328	1.452561933	0.017750737	0.195083398	0.194820783
Opa1	4	4406;5378;6723;23543	3	2.656962745	1.252438574	0.017818826	0.195446964	0.194820783
Skiv2l2	4	1175;2145;6724;30955	3	2.65677492	1.572540435	0.017821041	0.195087988	0.194820783
Sp110	4	2587;3193;6725;14201	3	2.656587125	1.409289358	0.017831528	0.194820783	0.194820783
Ppie	4	3460;4876;6741;17887	3	2.653586317	1.293582134	0.018042145	0.196736901	0.196210424
Polr3e	4	1375;2887;6743;18928	3	2.653211734	1.492994103	0.018055437	0.196498064	0.196210424
Supt5	4	4279;5161;6745;8229	3	2.652837267	1.262343269	0.018064152	0.196210424	0.196210424
Hmgcs1	4	2135;5852;6763;9112	3	2.649472219	1.302589719	0.018155872	0.196823751	0.196823751
Ehmt1	4	276;1576;15315;20232	2	2.646370413	1.503020365	0.018243604	0.197391554	0.197391554
Cox6c	4	2298;4078;6789;24849	3	2.644627922	1.367346106	0.01828363	0.197441987	0.197431287
Supt6	4	3275;4138;6799;10388	3	2.642769842	1.329557853	0.018460129	0.198963125	0.197431287
C330021F23Rik	4	1565;1584;28099;45418	2	2.642061502	1.257493773	0.018476671	0.198757714	0.197431287
Vhl	4	3735;6068;6805;32760	3	2.641656351	1.239472937	0.018489225	0.198510277	0.197431287
Polr2d	4	1090;3887;6807;10023	3	2.641285413	1.452311484	0.018496315	0.19820523	0.197431287
Vmn1r3	4	1306;6502;6816;14921	3	2.639617588	1.329074324	0.018546532	0.197983341	0.197431287
Vmn1r2	4	1306;6502;6816;14921	3	2.639617588	1.329074324	0.018546532	0.197983341	0.197431287
Mrps16	4	598;4989;6822;9194	3	2.638506971	1.463747008	0.018575333	0.197912373	0.197431287
Kpnb1	4	1553;1591;8050;12887	2	2.638309394	1.257290622	0.018577991	0.197563669	0.197431287
Vps28	4	2105;5473;6837;11527	3	2.635734846	1.313992733	0.018656123	0.198017371	0.197431287
Ddb1	4	4606;4857;6839;10441	3	2.635365705	1.263694729	0.018668973	0.197777755	0.197431287
Tuba1b	4	1466;3528;6840;12645	3	2.635181177	1.440187775	0.018671632	0.197431287	0.197431287
Ect2	4	2165;4029;6844;32125	3	2.634443342	1.37319757	0.018819034	0.198613735	0.198613735
Sdhd	4	1888;1923;6859;41242	3	2.631680433	1.540851893	0.018907357	0.199169385	0.198896475

Rad51d	4	3604;6674;6865;24529	3	2.63057702	1.221143581	0.018936749	0.199103331	0.198896475
Birc5	4	1322;4423;6868;15373	3	2.630025689	1.402999446	0.0189527	0.198896475	0.198896475
Dusp5	4	47;23588;45662;46957	1	2.627747977	1.313873988	0.019020789	0.199236517	0.199236517
Clp1	4	1096;5508;6905;8832	3	2.623246364	1.37623789	0.019278816	0.201561108	0.201301497
Top3a	4	1450;4502;6907;24319	3	2.622880987	1.388095818	0.019290041	0.201301497	0.201301497
Gabarapl2	4	48;9566;27157;70491	1	2.618620966	1.309310483	0.019407609	0.202150521	0.201722433
Cct4	4	4524;6341;6935;21158	3	2.617777178	1.207349704	0.019433751	0.202045871	0.201722433
Ckap5	4	4177;6202;6937;15164	3	2.617413438	1.218823715	0.019438773	0.201722433	0.201722433
B4galt1	4	439;2299;6943;26814	3	2.616322868	1.651655889	0.01960124	0.203031028	0.203031028
Katnb1	4	1076;5214;6951;30458	3	2.614870292	1.387112497	0.019643186	0.203088721	0.203088721
Ints4	4	3241;6507;6979;9193	3	2.609799884	1.230930726	0.019799893	0.204330508	0.203833033
Ccdc83	4	49;13006;56116;66060	1	2.609682493	1.304841247	0.019802404	0.203979378	0.203833033
Snrpf	4	4419;4928;6983;29725	3	2.609077262	1.257866819	0.019824707	0.203833033	0.203833033
AY761184	4	98;2952;6996;57098	3	2.606731696	1.758220815	0.02001435	0.205404628	0.205112856
Cenpa	4	473;2132;7003;15841	3	2.60547057	1.656891034	0.020056592	0.205460462	0.205112856
Ripk1	4	208;1654;14708;25095	2	2.605280129	1.529900242	0.020059398	0.205112856	0.205112856
Uqcc2	4	2291;6786;7013;74224	3	2.603671225	1.254750285	0.020108581	0.205239871	0.205239871
Fancl	4	50;23202;41720;49369	1	2.600924939	1.300462469	0.020204289	0.205840415	0.205840415
Atl2	4	3383;4701;7041;19186	3	2.598647173	1.289390864	0.020398955	0.207445108	0.207445108
Ctr9	4	2039;3865;7063;9062	3	2.594714212	1.377799572	0.02052243	0.208321321	0.208321321
Rabggta	4	1305;5456;7070;76700	3	2.593465478	1.352618065	0.020564819	0.20837275	0.20837275
Stt3b	4	294;1680;28261;45543	2	2.592022202	1.475856721	0.020605879	0.208410549	0.208410549
Prmt5	4	2055;4529;7131;12401	3	2.582637612	1.3414837	0.021140544	0.213431571	0.212736768
Iscu	4	2367;3045;7133;53439	3	2.582284229	1.409320352	0.021151622	0.213157948	0.212736768
Nup160	4	3618;3798;7140;27490	3	2.581048195	1.322421522	0.021193125	0.213191378	0.212736768
Rrp1	4	3190;5098;7145;9505	3	2.580166083	1.273873722	0.021222664	0.213104557	0.212736768
Scd2	4	2383;2632;7146;9354	3	2.579989737	1.438565742	0.021224141	0.212736768	0.212736768
Hist1h1e	4	1592;1712;37104;39609	2	2.57598984	1.233143858	0.021347616	0.213590935	0.213359303
Cyc1	4	1589;1713;9961;43076	2	2.575493781	1.233234868	0.021362681	0.213359303	0.213359303
Ppcs	4	2175;4959;7185;12210	3	2.573132155	1.315000449	0.021570344	0.215048628	0.214694695
Hyou1	4	1512;5404;7186;13335	3	2.572956827	1.334294782	0.021573298	0.214694695	0.214694695
Ints8	4	4201;7114;7205;10649	3	2.569630411	1.179281984	0.021681856	0.215391106	0.215391106
Arcn1	4	3859;7184;7227;10861	3	2.565790125	1.184151552	0.021939293	0.217561406	0.217561406
Dhx37	4	6358;6945;7242;27921	3	2.563178698	1.14684133	0.022027764	0.218051426	0.217950637
Pak2	4	184;5301;7250;13756	3	2.561788231	1.558573897	0.022061882	0.21800263	0.217950637
Mrps21	4	3830;4346;7258;32945	3	2.560399356	1.284293645	0.022109293	0.218085125	0.217950637
Ubash3b	4	55;26170;77079;78424	1	2.559614105	1.279807052	0.022134697	0.217950637	0.217950637
Cpsf6	4	1684;4395;7289;8952	3	2.555032435	1.360780698	0.02242123	0.220383322	0.21931939

Kif11	4	1548;1757;14860;56227	2	2.553955549	1.229611699	0.022450326	0.220281497	0.21931939
Gm3696	4	4742;5249;7296;29631	3	2.55382383	1.22502775	0.022452542	0.219916739	0.21931939
Itgb5	4	4610;7132;7301;13486	3	2.552961277	1.16630351	0.022482081	0.21982042	0.21931939
Gtf2e1	4	6258;7068;7303;31517	3	2.552616428	1.142033411	0.022492863	0.219541356	0.21931939
Pex14	4	4075;6964;7307;41921	3	2.551927023	1.181797748	0.022509405	0.21931939	0.21931939
Gm5082	4	1186;1776;14031;15085	2	2.544825069	1.263121776	0.022879979	0.222541673	0.222040961
Rfc4	4	2168;3183;7350;14938	3	2.544540577	1.399348357	0.022895191	0.222302354	0.222040961
Lyst	4	57;4971;22401;33494	1	2.54413468	1.41045014	0.022908041	0.222040961	0.222040961
Gnl3l	4	1944;4212;7362;9290	3	2.542487252	1.351851802	0.023083063	0.223349635	0.22288266
Naa50	4	3414;5477;7369;13518	3	2.541291084	1.243277926	0.02312427	0.223361248	0.22288266
Smc3	4	784;5000;7379;15522	3	2.539584318	1.409933748	0.023176998	0.223483907	0.22288266
Naa20	4	6426;6950;7380;23264	3	2.539413774	1.139906088	0.023186746	0.223192423	0.22288266
Acaca	4	3835;4340;7389;10589	3	2.537879954	1.278826323	0.023233419	0.22325676	0.22288266
Lars	4	1947;6863;7392;38112	3	2.537369112	1.25217372	0.023248632	0.223019091	0.22288266
Sec61b	4	58;8505;14755;14944	1	2.536597914	1.268298957	0.023274331	0.22288266	0.22288266
Kitl	4	631;1801;21165;26470	2	2.532962488	1.347457923	0.023532654	0.224970562	0.224724724
Haus2	4	1424;1803;38534;43086	2	2.532020777	1.23369805	0.023568545	0.224928521	0.224724724
Nsa2	4	2247;7355;7429;30306	3	2.531086378	1.22274252	0.023599857	0.224843002	0.224724724
Tsen34	4	2663;4151;7438;55597	3	2.529563062	1.320472526	0.023656868	0.225002203	0.224724724
Tmub1	4	59;16831;29345;63071	1	2.529190267	1.264595134	0.023667945	0.224724724	0.224724724
BC055324	4	5487;6339;7445;9410	3	2.528379584	1.16831253	0.023820812	0.22579218	0.225216966
Armc5	4	961;1816;27592;37934	2	2.525925621	1.286032002	0.023908249	0.226236872	0.225216966
Cpsf2	4	4683;5276;7462;58221	3	2.525510232	1.21801694	0.023925973	0.226021499	0.225216966
Skiv2l	4	3375;5697;7467;28644	3	2.524667598	1.232296247	0.023958319	0.225944751	0.225216966
Cdc23	4	2090;2385;7468;14614	3	2.524499141	1.45840796	0.023961716	0.225595714	0.225216966
Taf10	4	692;6831;7474;11467	3	2.523488894	1.356718665	0.023998197	0.22555881	0.225216966
Txn1	4	4603;5727;7478;10314	3	2.522815865	1.202376439	0.024024487	0.225426406	0.225216966
Rps20	4	1319;1877;7482;30052	3	2.522143209	1.555160943	0.024042506	0.225216966	0.225216966
Scgb1b20	4	6208;6305;7488;14905	3	2.521134927	1.157346795	0.024205564	0.226364599	0.226137744
Rps9	4	2134;2446;7490;18864	3	2.520799019	1.450093242	0.024221811	0.226137744	0.226137744
Stk11	4	3957;7225;7498;11313	3	2.51945632	1.169153917	0.024274834	0.226254425	0.226254425
Tpra1	4	61;15712;57160;68891	1	2.514745187	1.257372594	0.024575399	0.228674088	0.228674088
Tpt1	4	2851;4509;7545;31411	3	2.511597924	1.292439682	0.024687354	0.229333604	0.229333604
Thap11	4	4514;7485;7576;12440	3	2.506442538	1.147111682	0.025022923	0.232064746	0.232064746
Mtg2	4	1645;4848;7587;20054	3	2.504618479	1.330567713	0.025092488	0.232323983	0.23224242
Ncoa4	4	1211;5974;7592;38198	3	2.503790271	1.319751368	0.025125277	0.23224242	0.23224242
Etohi1	4	2795;5954;7606;9104	3	2.501474303	1.235677919	0.025213157	0.232669515	0.232669515
Mcrs1	4	2009;2988;7614;18291	3	2.500152883	1.409028379	0.025396007	0.233970141	0.2339311

Snap23	4	2789;4099;7620;27167	3	2.499162766	1.310974582	0.02543367	0.2339311	0.2339311
Srsf3	4	3171;6315;7627;21633	3	2.498008653	1.210989709	0.025481819	0.23398848	0.233975262
Elp2	4	2451;7222;7634;49953	3	2.496855643	1.209172897	0.025522288	0.233975262	0.233975262
Ddi2	4	1639;4678;7645;14872	3	2.495045989	1.335804466	0.025589638	0.234208115	0.234208115
Wdr75	4	1106;2346;7652;12576	3	2.493895802	1.519218627	0.025762444	0.235403809	0.234726859
Utp20	4	1647;3825;7653;11731	3	2.493731579	1.376238044	0.025764808	0.23504072	0.234726859
Rps10	4	2424;4863;7654;52738	3	2.493567378	1.288355538	0.025776771	0.234766253	0.234726859
Rbm17	4	5497;6853;7668;77030	3	2.491270909	1.143479623	0.025861402	0.23515343	0.234726859
Rps25	4	2376;3105;7669;11444	3	2.491107042	1.382084961	0.025864799	0.234801905	0.234726859
Cdh11	4	2490;3470;7682;65233	3	2.488978799	1.353849726	0.02595002	0.235193123	0.234726859
Pik3ap1	4	1616;1897;8216;28881	2	2.488927143	1.202090427	0.02595002	0.234811934	0.234726859
Mms19	4	3451;5245;7687;12941	3	2.488161242	1.23770365	0.025982661	0.234726859	0.234726859
Cdnf	4	65;33602;66824;77045	1	2.487227155	1.243613578	0.026143947	0.235802354	0.235802354
Rasgrp3	4	4371;6672;7707;14674	3	2.484896539	1.167135879	0.026239508	0.236282533	0.236282533
Hps4	4	66;9500;57450;59239	1	2.480612949	1.240306475	0.02653313	0.238541814	0.238253845
Olfr38	4	3437;7530;7740;42145	3	2.479529014	1.164176396	0.026579064	0.238570604	0.238253845
2310011J03Rik	4	3461;4226;7742;16753	3	2.479204476	1.279146977	0.026586449	0.238253845	0.238253845
Nsf	4	1062;6871;7758;11512	3	2.476611307	1.298876261	0.026692791	0.238823485	0.238611564
Ak2	4	4470;5248;7761;22724	3	2.476125708	1.21087445	0.026711844	0.238611564	0.238611564
Rcan1	4	906;1929;31261;79396	2	2.47475494	1.277203358	0.026900602	0.239913834	0.239468849
Cnot11	4	207;1930;27159;53405	2	2.474315955	1.486937713	0.02691419	0.23965219	0.239468849
Twistnb	4	3742;5567;7776;60268	3	2.473700641	1.214549263	0.026936492	0.239468849	0.239468849
Bub1b	4	2936;4865;7787;59725	3	2.471925353	1.264222631	0.027005615	0.239701666	0.239603905
Nup85	4	3807;5070;7791;21162	3	2.471280441	1.231208053	0.027037518	0.239603905	0.239603905
Gm5862	4	3930;6180;7822;11978	3	2.466294038	1.187304078	0.027369099	0.242157967	0.241703729
Yars	4	3966;5255;7824;17798	3	2.465973042	1.218879396	0.027384459	0.241910499	0.241703729
Polr3k	4	3265;5956;7829;31342	3	2.465170925	1.211657963	0.027418577	0.241829253	0.241703729
Timm17a	4	3214;7574;7834;12496	3	2.464369342	1.165585485	0.027449298	0.241718348	0.241703729
Nop56	4	1066;1959;8355;16803	2	2.461685899	1.250187451	0.027683694	0.243398529	0.241703729
Espl1	4	2278;3731;7852;19994	3	2.46148805	1.340742084	0.027694772	0.243113067	0.241703729
D830030K20Rik	4	2217;5194;7857;30552	3	2.460688913	1.275620127	0.027726822	0.24301232	0.241703729
Ppp2r1a	4	3158;4724;7873;8894	3	2.458135229	1.259782555	0.02784306	0.243648596	0.241703729
Psmd11	4	231;3486;7874;32618	3	2.457975803	1.593892255	0.027847491	0.243306013	0.241703729
Eif4a1	4	3142;3602;7875;14818	3	2.457816399	1.315841282	0.027850445	0.242951616	0.241703729
Cdc7	4	4617;5203;7879;24798	3	2.457178991	1.205000955	0.027880428	0.24283374	0.241703729
Ipo7	4	2971;7566;7882;11600	3	2.456701157	1.171375634	0.027897856	0.242607055	0.241703729
Rpl14	4	2434;4428;7883;18963	3	2.456541921	1.297783165	0.027900662	0.242254116	0.241703729
Slmo2	4	3926;4562;7887;10739	3	2.455905188	1.245985997	0.028057665	0.24323904	0.241703729

Olfr456	4	430;6560;7899;75710	3	2.453997006	1.397909454	0.028134911	0.243530551	0.241703729
Mrps14	4	5351;6909;7901;29324	3	2.453679269	1.134763548	0.028151305	0.243295256	0.241703729
Arfrp1	4	2377;4675;7902;61471	3	2.453520432	1.288287543	0.02815485	0.242949808	0.241703729
Wdr20rt	4	139;1979;36928;79636	2	2.453086838	1.537141567	0.028180992	0.242800122	0.241703729
Psmd7	4	269;2260;7907;9637	3	2.452726562	1.666842288	0.028186162	0.242470478	0.241703729
Psmd8	4	7311;7738;7909;29993	3	2.45240916	1.087151976	0.028199307	0.242210352	0.241703729
Psma4	4	2205;3120;7914;10885	3	2.451616022	1.378597657	0.028233868	0.242134692	0.241703729
Lsm5	4	2415;4081;7917;32099	3	2.45114039	1.313924485	0.028256909	0.241960615	0.241703729
Ints3	4	3953;4538;7919;47211	3	2.450823406	1.245163586	0.028270201	0.241703729	0.241703729
Atp5b	4	2868;6603;7925;21318	3	2.449872956	1.199742582	0.028437247	0.242760172	0.24195792
Lonp1	4	2649;3591;7929;16670	3	2.449239738	1.330768531	0.028466048	0.242635036	0.24195792
Qars	4	2926;3423;7930;42112	3	2.449081486	1.331029949	0.028469593	0.242295334	0.24195792
Ppa1	4	1202;3272;7931;8931	3	2.448923255	1.42971106	0.028473286	0.24195792	0.24195792
Naf1	4	449;7472;7943;17764	3	2.447026098	1.366010779	0.028557621	0.242305772	0.241958822
Krt26	4	182;3521;7945;52261	3	2.446710196	1.614690577	0.028571061	0.242051951	0.241958822
Fen1	4	2430;3184;7950;57953	3	2.445920803	1.363344692	0.028603407	0.241958822	0.241958822
Ap5b1	4	72;6105;20329;35872	1	2.44292263	1.321648952	0.028836179	0.243558828	0.243558828
Rpl18	4	1015;2017;9081;71050	2	2.436991735	1.248771324	0.029083867	0.245279806	0.244909852
Mon1b	4	73;21255;43679;68100	1	2.436948641	1.218474321	0.029083867	0.244909852	0.244909852
Kif3b	4	74;34487;38905;60112	1	2.431056156	1.215528078	0.029333033	0.246636031	0.246636031
Cdc45	4	1867;2042;17242;23622	2	2.426571843	1.161803682	0.029551921	0.24810282	0.247587594
Emg1	4	1741;2044;9485;62582	2	2.425743922	1.170939868	0.02956994	0.247881345	0.247587594
Mon1a	4	75;15054;48091;60610	1	2.425242987	1.212621494	0.029579245	0.247587594	0.247587594
Mat2a	4	1254;2056;13002;35847	2	2.420793818	1.214083659	0.029807881	0.249127843	0.249127843
Rfwd2	4	77;15925;20064;49798	1	2.413846276	1.206923138	0.03008378	0.251057913	0.251057913
Dbpht2	4	1073;2089;14746;45190	2	2.407332683	1.231160898	0.030352146	0.252919453	0.252721944
Orc5	4	1435;2091;9545;15653	2	2.406523898	1.190812868	0.03037371	0.252721944	0.252721944
Hist1h2bn	4	1279;2099;10697;16685	2	2.403296694	1.205532147	0.030581668	0.254073594	0.253778162
Mboat4	4	79;25826;49984;69300	1	2.40274266	1.20137133	0.030591564	0.253778162	0.253778162
Tasp1	4	80;32699;56823;77687	1	2.39729614	1.19864807	0.030841912	0.25547536	0.25547536
Aldh1a2	4	81;23162;35550;62618	1	2.391917485	1.195958742	0.031087237	0.257125994	0.257125994
Dnaja2	4	82;8607;31330;39803	1	2.386605027	1.193302514	0.031338619	0.258821756	0.258821756
Rhbdf2	4	84;3243;12826;48064	1	2.376172347	1.471503346	0.031838427	0.262561209	0.262333275
Cdk1	4	1664;2169;9777;25340	2	2.375586928	1.160340288	0.031857776	0.262333275	0.262333275
Thoc3	4	1335;2178;12094;30500	2	2.372091192	1.189237211	0.032070165	0.263693268	0.263693268
Isca1	4	1376;2195;12663;63144	2	2.365528535	1.182898053	0.032350495	0.265607076	0.265283648
Prss44	4	858;2196;60052;61565	2	2.365144128	1.248282041	0.032358618	0.265283648	0.265283648
Zmat2	4	2004;2202;21841;53651	2	2.362841466	1.13107478	0.032537037	0.266355243	0.26625502

Stx18	4	848;2206;16661;20219	2	2.361309946	1.248646004	0.032572484	0.26625502	0.26625502
Gm9758	4	1187;2218;9664;42283	2	2.356732493	1.200307776	0.032817367	0.267864558	0.267476677
Olfr177	4	88;56513;64305;79060	1	2.356034469	1.178017235	0.032835386	0.267620377	0.267476677
Olfr330	4	962;2222;23713;72580	2	2.355212344	1.228982535	0.032865664	0.267476677	0.267476677
Ogdh	4	426;2231;9602;29696	2	2.351802292	1.34281908	0.033081745	0.268843354	0.268819346
Rpl5	4	1748;2236;10704;55155	2	2.349913939	1.145121349	0.033126941	0.268819346	0.268819346
a	4	90;13735;32434;58261	1	2.346307387	1.173153694	0.033347896	0.270219601	0.270219601
Nudcd3	4	216;2254;9498;14321	2	2.343151722	1.437104376	0.033548617	0.271452073	0.271427975
Wdr5	4	2245;2259;17249;18150	2	2.341283206	1.10879938	0.033594256	0.271427975	0.271427975
Ddx6	4	92;4763;18522;39921	1	2.336794826	1.35290692	0.033826732	0.272911335	0.272911335
Rhoq	4	93;39588;46676;72410	1	2.332116083	1.166058042	0.034077965	0.274541531	0.274541531
Cct7	4	2115;2289;14976;26741	2	2.330160888	1.112998404	0.03413542	0.274608139	0.274608139
Crkl	4	95;10721;22187;57547	1	2.322908184	1.161454092	0.034576297	0.277754628	0.277754628
Atp6v1d	4	96;9695;32654;47316	1	2.318376935	1.159188468	0.034813942	0.27926184	0.27893571
Rpp21	4	2207;2322;8601;30948	2	2.31809868	1.103329364	0.034823247	0.27893571	0.27893571
Zfp451	4	97;13670;36824;64593	1	2.313892813	1.156946407	0.035068425	0.280497161	0.280497161
Gm7861	4	98;59698;74567;78848	1	2.309454851	1.154727426	0.035328668	0.280568927	0.280568927
Gm7849	4	98;59698;74567;78848	1	2.309454851	1.154727426	0.035328668	0.280568927	0.280568927
Gm6696	4	98;11575;34689;65008	1	2.309454851	1.154727426	0.035328668	0.280568927	0.280568927
Gm14851	4	98;6996;25063;57098	1	2.309454851	1.241246454	0.035328668	0.280568927	0.280568927
Defa25	4	98;20832;34689;65008	1	2.309454851	1.154727426	0.035328668	0.280568927	0.280568927
Psma1	4	890;2353;8084;12819	2	2.306927575	1.223752055	0.03551846	0.281675513	0.281341473
Ppp1r2	4	1192;2354;10231;19334	2	2.306569751	1.183006029	0.035526731	0.281341473	0.281341473
Polr2i	4	2292;2360;8067;10996	2	2.304426096	1.093778066	0.035583151	0.281389142	0.281389142
4930402K13Rik	4	272;2369;15721;75906	2	2.30122114	1.390062113	0.035798494	0.282691647	0.282691647
Cpsf4	4	1047;2379;20217;22232	2	2.297674792	1.198015586	0.036017973	0.284023084	0.284023084
Trim28	4	1132;2391;11979;56856	2	2.293439439	1.185773834	0.036259902	0.285527547	0.285399487
Ush1g	4	102;66184;71963;75420	1	2.292146275	1.146073138	0.036294758	0.285399487	0.285399487
Mars	4	1036;2400;8836;24968	2	2.290277309	1.197017955	0.036480857	0.286459389	0.286338201
A930018P22Rik	4	103;34706;35439;38457	1	2.287925603	1.143962802	0.036537573	0.286501784	0.286338201
Clns1a	4	1014;2409;19732;37353	2	2.287127405	1.19895372	0.036567999	0.286338201	0.286338201
Cenph	4	105;23921;25667;29573	1	2.27960629	1.139803145	0.037042256	0.289645537	0.289645537
Nup107	4	1281;2435;9678;23943	2	2.278095532	1.163583316	0.037212256	0.290567864	0.289973962
Peo1	4	1879;2439;9891;33083	2	2.276714864	1.110990882	0.03724918	0.290449961	0.289973962
Rabgef1	4	106;6948;12374;45047	1	2.275506105	1.23173229	0.037292012	0.290378388	0.289973962
Ifi204	4	761;2443;27402;43389	2	2.275336536	1.235179333	0.037292012	0.289973962	0.289973962
Vgll4	4	107;19338;33908;52223	1	2.271444574	1.135722287	0.037542064	0.291512302	0.291512302
Atp6v1a	4	2229;2461;8144;59635	2	2.269162792	1.085704895	0.037745739	0.292686753	0.292686753

Cyp2a22	4	108;31710;35844;66167	1	2.267420978	1.133710489	0.037801569	0.29271312	0.29271312
Tgs1	4	2305;2481;19761;22161	2	2.262357601	1.079009461	0.038203011	0.295411925	0.295411925
Arpc4	4	1516;2495;8566;78133	2	2.257627642	1.133681725	0.038471082	0.297073376	0.297073376
Krt24	4	111;27131;68578;75979	1	2.255570901	1.12778545	0.038536069	0.297164189	0.297164189
Huwe1	4	2127;2506;8141;10001	2	2.253930467	1.086836453	0.038707989	0.29807821	0.29783098
Vmn1r5	4	2365;2509;30114;68719	2	2.25292506	1.07248228	0.038741073	0.297922054	0.29783098
Chst7	4	112;9211;67925;75715	1	2.251692239	1.12584612	0.038782576	0.29783098	0.29783098
Mrps27	4	2121;2520;19013;55381	2	2.249249182	1.085651349	0.038994522	0.299047277	0.298884429
Mxd3	4	113;8010;8583;10461	1	2.247848201	1.1239241	0.039038831	0.298976401	0.298884429
Abt1	4	1966;2529;8312;58220	2	2.246253975	1.094771167	0.039216955	0.299929121	0.298884429
Nup133	4	1934;2530;7967;57982	2	2.245921855	1.096857261	0.039225964	0.299587631	0.298884429
Prim1	4	1702;2533;11318;17292	2	2.244926311	1.113729698	0.039261559	0.299449844	0.298884429
Cpa3	4	114;30700;67807;75409	1	2.244038176	1.122019088	0.039283419	0.299207812	0.298884429
Rrm2	4	1247;2536;8353;41284	2	2.243931986	1.155901295	0.039294496	0.298884429	0.298884429
Lrfn2	4	115;19071;20148;52081	1	2.24026157	1.120130785	0.039532437	0.300285162	0.300285162
Noc3l	4	2418;2554;18845;44017	2	2.237991493	1.064594005	0.039736407	0.301424403	0.301424403
Akr1c6	4	117;48089;58823;70392	1	2.232806315	1.116403158	0.040033132	0.303263193	0.303263193
Ube2t	4	118;7851;19925;57445	1	2.229126553	1.184514753	0.040289535	0.304791966	0.304791966
Gm5797	4	1070;2592;11547;22569	2	2.22559156	1.170969256	0.040528214	0.306182703	0.305768943
Mpp2	4	119;6883;47752;55954	1	2.225477983	1.217518836	0.040528214	0.305768943	0.305768943
Dpy19l2	4	120;19661;47191;77654	1	2.221860082	1.110930041	0.040906615	0.30820733	0.307221056
Cand1	4	1130;2604;31854;38647	2	2.221714884	1.16211058	0.040916511	0.307866414	0.307221056
Gnl2	4	2321;2607;17095;39882	2	2.220748607	1.064228252	0.040949152	0.307697328	0.307221056
Uqcrc2	4	1505;2610;12380;26869	2	2.219783481	1.122060095	0.040984156	0.30754643	0.307221056
Psmb2	4	2228;2611;9569;11757	2	2.219462028	1.069197325	0.040995824	0.307221056	0.307221056
NO_CURRENT_144	4	121;3444;51505;75116	1	2.218272342	1.40222196	0.041165381	0.308078181	0.307664535
Tfdp1	4	2395;2619;10280;11877	2	2.216894988	1.058816099	0.041208656	0.307989196	0.307664535
Vmn1r220	4	2421;2620;8478;12901	2	2.216574679	1.057292442	0.041220325	0.307664535	0.307664535
Tti1	4	2136;2628;18886;64999	2	2.214016763	1.07297106	0.041425329	0.308781857	0.308430618
Phb2	4	1053;2629;9997;20393	2	2.213697591	1.169229112	0.041433452	0.308430618	0.308430618
Prim2	4	2051;2642;10033;64789	2	2.209559771	1.076887894	0.041714521	0.310109413	0.310109413
Marveld3	4	124;22129;63638;72275	1	2.207685181	1.103842591	0.041906971	0.31112582	0.31112582
Tsr2	4	2503;2664;9556;28816	2	2.202605164	1.048275339	0.042201627	0.312897324	0.312832302
Rars2	4	1448;2668;11811;24498	2	2.201347091	1.121187243	0.04224889	0.312832302	0.312832302
Clcn4-2	4	126;12528;35829;53383	1	2.200769092	1.100384546	0.042396588	0.313510134	0.313333218
Ruvbl2	4	2036;2673;10011;27795	2	2.199777247	1.07460573	0.042428786	0.313333218	0.313333218
Zfp119a	4	128;53023;54931;62848	1	2.193962439	1.096981219	0.042892557	0.316339687	0.316339687
Arhgdia	4	283;2700;36154;75369	2	2.191352386	1.347760212	0.042990923	0.316646865	0.316646865

Slc22a17	4	130;41735;63516;71520	1	2.187261828	1.093630914	0.043384833	0.319127167	0.319127167
Xpo1	4	1920;2718;13729;71781	2	2.185784236	1.077784898	0.043445684	0.319154281	0.319154281
A530064D06Rik	4	132;5063;18037;52356	1	2.180664022	1.284340757	0.043886709	0.321970428	0.321970428
Vmn2r109	4	133;14733;67318;77105	1	2.177402699	1.088701349	0.04414193	0.323417843	0.323417843
Orc2	4	134;11576;39549;49331	1	2.174165928	1.087082964	0.044385927	0.32477933	0.324757889
Ttc12	4	1027;2760;29197;64887	2	2.172939356	1.159118685	0.044441166	0.324757889	0.324757889
Plxna2	4	135;24746;40510;76618	1	2.170953345	1.085476673	0.044632434	0.325729253	0.325729253
Mrpl17	4	1012;2777;9905;50990	2	2.167797846	1.159451968	0.044886474	0.327155591	0.327155591
Bricd5	4	137;31697;41754;65832	1	2.164599321	1.082299661	0.045135935	0.328544882	0.32827707
Lias	4	2769;2790;12102;43510	2	2.163888115	1.022249148	0.045157942	0.32827707	0.32827707
Lasp1	4	138;32324;78231;78384	1	2.16145719	1.080728595	0.045375353	0.329428601	0.329428601
Bysl	4	1671;2811;9126;17248	2	2.15761221	1.087113328	0.045657307	0.331045126	0.330695968
Tuba3b	4	1794;2812;45784;57391	2	2.157314574	1.077417773	0.045668385	0.330695968	0.330695968
Gpr135	4	141;10872;38431;63805	1	2.152166328	1.076083164	0.046126543	0.333580942	0.333580942
Dmc1	4	143;24257;33048;54130	1	2.146082181	1.07304109	0.046618819	0.336704869	0.336704869
Twf2	4	145;43376;61189;63440	1	2.140082994	1.070041497	0.04712232	0.339901693	0.339874876
Inhbc	4	1075;2876;20899;35119	2	2.138491296	1.141288577	0.047179479	0.339874876	0.339874876
Nup62	4	2111;2882;9531;39791	2	2.136748998	1.048779413	0.047390686	0.340956444	0.340838132
Zc3h4	4	814;2886;10979;36169	2	2.135589562	1.17914233	0.047435291	0.340838132	0.340838132
Vmn2r110	4	147;13600;31017;67755	1	2.134166441	1.06708322	0.047622571	0.341743979	0.341305283
Eprs	4	2253;2891;11905;40727	2	2.134142616	1.039282461	0.047622571	0.341305283	0.341305283
Hsd3b2	4	148;21582;57875;75098	1	2.13123845	1.065619225	0.047871294	0.342647993	0.342122794
4933402P03Rik	4	741;2902;34817;36274	2	2.130968481	1.19080203	0.047883553	0.342296896	0.342122794
Rcc1	4	1608;2905;8631;12781	2	2.13010498	1.08315695	0.047920477	0.342122794	0.342122794
Aaed1	4	149;2974;10497;12121	1	2.128330287	1.412942329	0.048117949	0.343093881	0.342830857
Slc13a4	4	477;2913;16763;70832	2	2.127806843	1.252064033	0.048142467	0.342830857	0.342830857
Fcf1	4	2472;2922;15921;33953	2	2.125229278	1.024113007	0.04837834	0.344071682	0.344071682
Slitrk3	4	2836;2927;16381;46536	2	2.123800867	1.005804329	0.048565916	0.344966294	0.344928212
Lbx1	4	151;11578;29018;62261	1	2.122572389	1.061286194	0.048622336	0.344928212	0.344928212
Ubr4	4	152;15531;23259;41850	1	2.119722139	1.059861069	0.048871797	0.346257924	0.346257924
1700003F12Rik	4	153;10055;21442;60779	1	2.116890687	1.058445343	0.049110624	0.347509018	0.347509018
Naa25	4	718;2957;8542;27208	2	2.115283402	1.190011676	0.049306767	0.348455289	0.348039707
Retn	4	154;12822;48029;63495	1	2.114077788	1.057038894	0.049359938	0.348390054	0.348039707
9030624G23Rik	4	1534;2962;8404;22186	2	2.113872579	1.084154725	0.04937264	0.348039707	0.348039707
Dgkd	4	155;35977;36537;63565	1	2.111283201	1.055641601	0.049617522	0.349324877	0.349244254
Zfp954	4	1984;2975;36114;54487	2	2.110216018	1.04820645	0.049668626	0.349244254	0.349244254
Mfsd1	4	983;2981;50907;73330	2	2.108533986	1.143746199	0.049868313	0.350207284	0.349767325
Olfr457	4	156;17399;34774;54333	1	2.108506693	1.054253346	0.049868313	0.349767325	0.349767325

C1qbp	4	1050;2985;16291;24354	2	2.10741459	1.134197891	0.050045845	0.350572088	0.350400473
Cpne4	4	157;10059;42247;73828	1	2.10574803	1.052874015	0.050120433	0.350654606	0.350400473
Polr1a	4	2357;2993;8814;18867	2	2.10518048	1.02367965	0.05014687	0.350400473	0.350400473
Syp	4	158;27612;54606;61506	1	2.103006988	1.051503494	0.050373439	0.351543634	0.351543634
Psma2	4	2411;3005;11188;25091	2	2.101840958	1.019590823	0.050564412	0.352435842	0.352435842
Col24a1	4	161;27184;75607;76835	1	2.094887375	1.047443688	0.051128764	0.355925049	0.355730313
Itga10	4	1792;3033;42444;53474	2	2.094102498	1.056497402	0.051164507	0.355730313	0.355730313
Vdac3	4	162;22568;36546;76574	1	2.09221463	1.046107315	0.051371136	0.356722699	0.356722699
Nid1	4	163;19723;26832;38217	1	2.089558433	1.044779216	0.051622517	0.358022996	0.358022996
Pfdn5	4	2257;3055;12864;19680	2	2.088074364	1.023692036	0.051809797	0.358876053	0.358876053
Rhox1	4	1440;3065;39949;71044	2	2.085349256	1.08327889	0.052065019	0.360197026	0.360085975
Sgms1	4	165;21378;34674;55484	1	2.084294879	1.04214744	0.052113464	0.360085975	0.360085975
Chmp6	4	2243;3073;15904;24464	2	2.083175841	1.022881393	0.052291735	0.360871143	0.360430611
Pcf11	4	740;3078;16883;33960	2	2.081820452	1.174609346	0.052357017	0.360875587	0.360430611
Gpr107	4	166;27764;38879;51777	1	2.081687129	1.040843565	0.052357017	0.360430611	0.360430611
Vmn1r9	4	333;3081;35144;70894	2	2.081008322	1.287699974	0.052518746	0.361098716	0.360474725
Nop16	4	1632;3083;23962;25575	2	2.08046736	1.064599967	0.052541491	0.360810757	0.360474725
Tnpo3	4	3077;3088;14597;18058	2	2.079116555	0.980467347	0.052610318	0.360839566	0.360474725
Ubtf	4	2063;3089;8864;9022	2	2.078846668	1.03253951	0.052621691	0.360474725	0.360474725
Hist1h2ah	4	2726;3094;15688;26723	2	2.0774986	0.995475481	0.052810892	0.36132746	0.361050724
Mast1	4	1767;3096;16969;35862	2	2.076960009	1.052677613	0.052835114	0.361050724	0.361050724
Mrpl20	4	1338;3107;10056;23341	2	2.074004237	1.089566582	0.053094323	0.362378492	0.361938041
Usp46	4	169;32591;55216;67085	1	2.073957694	1.036978847	0.053094619	0.361938041	0.361938041
Trnt1	4	2849;3116;19313;70693	2	2.071593993	0.987813478	0.05333699	0.36314685	0.36280597
Hdac3	4	170;11930;15697;22663	1	2.071411872	1.035705936	0.053351908	0.36280597	0.36280597
Prpf19	4	2381;3119;13317;24587	2	2.070792194	1.010885171	0.05351083	0.363443997	0.363443997
5031439G07Rik	4	594;3132;40237;74601	2	2.06732701	1.200790405	0.053805782	0.36500326	0.364808474
Adgra3	4	172;22700;55036;59815	1	2.066365136	1.033182568	0.053842411	0.364808474	0.364808474
Zfp534	4	2285;3138;12615;25896	2	2.065732757	1.014617122	0.05400222	0.365447752	0.36522603
Smarca5	4	2338;3143;8790;10328	2	2.064406644	1.011153306	0.054072081	0.365477517	0.36522603
Fam187a	4	173;13435;18162;76355	1	2.063863874	1.031931937	0.054100291	0.36522603	0.36522603
Ckm	4	174;16604;32356;69621	1	2.061377124	1.030688562	0.054353593	0.366492884	0.366492884
Tfap4	4	175;6342;17875;19794	1	2.058904719	1.183550571	0.054599805	0.367708937	0.367265914
Myh9	4	2172;3164;11957;13514	2	2.058860931	1.019034696	0.054599805	0.367265914	0.367265914
Gemin2	4	1976;3167;15038;75319	2	2.058071828	1.031365225	0.054761238	0.367908534	0.367908534
Nol11	4	2419;3177;8387;17950	2	2.055447112	1.003691645	0.055014392	0.369165082	0.368773168
Taf7	4	2072;3179;11013;46616	2	2.054923205	1.023985234	0.055049248	0.368955526	0.368773168
Cd164l2	4	177;15874;41722;55228	1	2.054002293	1.027001147	0.055088093	0.368773168	0.368773168

Xbp1	4	179;20254;70682;77177	1	2.049155321	1.02457766	0.05558672	0.371665457	0.371665457
Olfr209	4	209;3209;59189;66532	2	2.047105686	1.343153244	0.055818162	0.372766504	0.37251374
Herc4	4	180;11478;21853;78968	1	2.046752243	1.023376121	0.055954191	0.373228495	0.37251374
Ykt6	4	2302;3211;11034;28780	2	2.046587237	1.007257696	0.055968961	0.372881515	0.37251374
Gemin5	4	2670;3212;10709;14366	2	2.04632814	0.987774106	0.055980481	0.37251374	0.37251374
Olfr640	4	181;20411;24541;69974	1	2.04436257	1.022181285	0.05621665	0.373639948	0.373639948
Sp7	4	751;3235;64409;67915	2	2.04039207	1.158724364	0.056536267	0.375317457	0.375317457
Foxr2	4	183;11309;49973;70365	1	2.039622849	1.019811424	0.056696372	0.375933305	0.375933305
Nifk	4	2081;3252;19683;42652	2	2.036032844	1.017111397	0.057024556	0.377660847	0.377660847
Krt71	4	185;54138;55062;60333	1	2.034935005	1.017467502	0.057204747	0.378405332	0.378405332
Zrsr2	4	2429;3263;23531;29070	2	2.033224856	0.995743254	0.057303999	0.378613288	0.378613288
Sall1	4	187;37239;72049;78957	1	2.030297922	1.015148961	0.057713712	0.380869571	0.380869571
Asic1	4	2875;3283;13108;63651	2	2.028144713	0.972162319	0.057942644	0.381928902	0.381125341
Gpi1	4	188;26851;49696;51240	1	2.027998078	1.013999039	0.057955936	0.38156603	0.381125341
Cox20	4	2831;3284;54506;62702	2	2.027891557	0.97406174	0.057957266	0.381125341	0.381125341
Ifnl3	4	189;19709;72762;73464	1	2.025710521	1.01285526	0.058207465	0.382320329	0.382320329
Smg6	4	190;10718;13206;16371	1	2.023435123	1.011717561	0.058453825	0.383487315	0.383487315
Akirin2	4	2593;3308;16656;22266	2	2.021839904	0.983413368	0.058544068	0.38362856	0.38362856
4933427D06Rik	4	191;66687;71860;74775	1	2.021171755	1.010585877	0.058691618	0.384144553	0.38390689
Ddx21	4	2330;3318;23869;39579	2	2.019331939	0.996579657	0.058923208	0.385208747	0.38390689
Dgcr14	4	1128;3319;11912;44659	2	2.019081578	1.09481153	0.058935762	0.384840187	0.38390689
Oas1d	4	192;30959;61832;63791	1	2.018920292	1.009460146	0.058948316	0.384472489	0.38390689
Timm50	4	3208;3323;15090;18323	2	2.018080924	0.954830224	0.058987752	0.384280768	0.38390689
Hcfc1	4	1350;3324;8497;9000	2	2.017830957	1.069616165	0.058999124	0.38390689	0.38390689
AI846148	4	193;16834;47908;67846	1	2.016680611	1.008340306	0.059195267	0.384734778	0.384734778
Kcnn3	4	194;31959;64822;67250	1	2.01445259	1.007226295	0.059444285	0.385904003	0.385904003
Thumpd1	4	195;10433;23722;57798	1	2.012236108	1.006118054	0.059693008	0.387068597	0.386918979
Ing3	4	2595;3350;12661;26201	2	2.011359339	0.979819505	0.059739237	0.386918979	0.386918979
Hist2h3c2	4	3097;3359;8126;50326	2	2.009131418	0.956314526	0.059983233	0.387599991	0.387599991
Hist2h3c1	4	3097;3359;8126;50326	2	2.009131418	0.956314526	0.059983233	0.387599991	0.387599991
Paf1	4	3174;3373;47301;53170	2	2.00567817	0.952045306	0.060423668	0.389994608	0.389346372
Ly6h	4	198;19943;21376;26103	1	2.005654728	1.002827364	0.060423668	0.389544268	0.389346372
Speer3	4	1180;3376;57989;78428	2	2.004940143	1.083861148	0.060465466	0.389364125	0.389346372
4930524N10Rik	4	199;38702;55297;75750	1	2.003483243	1.001741621	0.060666482	0.390208492	0.389346372
Lhx2	4	2853;3384;58174;69928	2	2.002975427	0.964760138	0.06069617	0.389950189	0.389346372
Wac	4	3014;3386;13353;23751	2	2.002485009	0.95756104	0.060727334	0.389701959	0.389346372
Reep4	4	1475;3387;15249;29158	2	2.002239914	1.052293685	0.06074166	0.389346372	0.389346372
Arf3	4	200;50633;60781;78484	1	2.001322724	1.000661362	0.060918159	0.39002991	0.389925441

Ptms	4	476;3395;26414;35138	2	2.000281881	1.209854036	0.060973693	0.389938293	0.389925441
