# Supplementary material for: Reovirus infection is regulated by NPC1 and endosomal cholesterol homeostasis
Source: PLoS Pathog. 2022 Mar 9;18(3):e1010322. doi: 10.1371/journal.ppat.1010322 (PMC8906592; doi:10.1371/journal.ppat.1010322)
Supplement: S2 Table — (PDF) [file ppat.1010322.s002.pdf]

Gene Symbol	Gene ID	Gene Accession	Sequence	zscorerun1	zscorerun2	zscorerun3	Average	numhits
MGAT4A	11320	NM_012214	GAAUAA, GAGUG	4.54	46.42	69.31	40.09	3
RTN4R	65078	NM_023004	JCUGUU, CGAAUC	4.51	39.1	67.51	37.04	3
F11R	50848	NM_144501	GAUUUA, GGGAA	8.58	59.5	38.73	35.60	3
PROSC	11212	NM_007198	GAGAA, GGACAU	4.61	49.09	52.92	35.54	3
ALOX12B	242	NM_001139	GAUCAA, GAUCAU	1.54	27.48	38.79	22.60	2
RPS6	6194	NM_001010	GUCCGC, UGGACU	2.21	24.93	30.32	19.15	2
C1QTNF7	114905	NM_031911	CCGCUA, GGAAAC	2.28	22.48	29.54	18.10	2
FAU	2197	NM_001997	GGUAAA, AAGAAG	5.75	21.41	27.03	18.06	3
RPLP0	6175	NM_001002	GCGCAA, ACAAAC	3.06	29.59	21.5	18.05	3
RPS3	6188	NM_001005	GGUUG, GGACCC	-0.15	22.76	30.55	17.72	2
HCA66	55813	NM_018428	GACUCA, GAUAAU	2.76	28.27	19.63	16.89	2
RPS24	6229	NM_001026	CGGGAA, CACCGU	2.11	19.04	27.92	16.36	2
HOXA7	3204	NM_006896	GACAAG, GAAAGA	7.93	21.8	19.27	16.33	3
HINT2	84681	NM_032593	GGGAAU, GAACCU	0.07	24.57	24.2	16.28	2
MGC71745	133418	NM_198449	AGAAUA, GAAGAU	3.86	42.42	0.8	15.69	2
RPS13	6207	NM_001017	GCGAGU, UGACAU	-0.31	29.18	17.53	15.47	2
RPS3A	6189	NM_001006	ACGGAA, GAUUGA	0.18	25.97	20.03	15.39	2
C10ORF81	79949	NM_024889	UCAGAA, AGGAAC	2.87	21.35	18.72	14.31	2
ATXN2L	11273	NM_017492	AGUGA, GAGGAG	1.88	19.7	21.26	14.28	2
PKHD1L1	93035	NM_177531	JCCAUU, AGACAU	5.91	17.24	19.12	14.09	3
RPS16	6217	NM_001020	GGUCAC, AUAAGC	-0.04	20.09	19.95	13.33	2
RPS7	6201	NM_001011	AAUCUU, UCGUGA	2.95	13.95	22.1	13.00	2
RPS4X	6191	NM_001007	AAGAAU, GCAUUC	0.07	20.09	18.48	12.88	2
RPS10	6204	NM_001014	CAAGAA, CGGAUU	0.81	23.37	14.24	12.81	2
PPP1R3F	89801	NM_033215	GCGCUA, GCACAU	4.76	18.33	15.18	12.76	3
RPS9	6203	NM_001013	AAAUUU, GGAGG	0.89	26.25	10.25	12.46	2
POLR1A	25885	NM_015425	GAAAUU, ACAUGC	2.22	12.49	21.81	12.17	2
RPS17	6218	NM_001021	GAAUUU, GCGAAU	1.05	16.59	18.38	12.01	2
FBL	2091	NM_001436	AUCUUU, GCGUAA	4.65	12.12	18.12	11.63	3
RPS14	6208	NM_005617	GUGUCU, CCGUGU	0.78	18.67	13.69	11.05	2
CCNE1	898	NM_057182	JGUAUA, GGACAA	3.27	14.44	15.15	10.95	3
RPS18	6222	NM_022551	GACAUU, CAUUAA	0.03	19.31	13.48	10.94	2
MGC3040	66000	NM_001136469	AGCGAUA, GAGUA	3.05	11.74	17.85	10.88	3

MGC20481	90416	NM_001080792	GAUCAA, GCUAA	3.23	5.72	22.34	10.43	3
ARRDC2	27106	NM_015683	GGACUA, GGGAAA	-0.92	11.28	20.13	10.16	2
IL31RA	133396	NM_139017	ACAUAU, GAAACA	0.4	13.09	16.95	10.15	2
CYP2A7	1549	NM_030589	GUCACU, CCACGA	5.03	11.25	13.98	10.09	3
KRT18	3875	NM_199187	GCUGGA, CACACA	1.71	11.59	16.89	10.06	2
FLJ12975	79867	NM_024809	GACUCA, GGAACU	2.99	14.16	12.98	10.04	2
ZNF219	51222	NM_001102454	CCGCCA, GAUUGU	4.94	19.89	5.07	9.97	3
RPS4Y1	6192	NM_001008	GAGAUA, UUGACA	0.82	15.83	13.05	9.90	2
ATF7	11016	NM_006856	CCUUUG, GAAGU	4.13	16.97	8.21	9.77	3
C6ORF204	387119	NM_206921	GCGCUU, AAUUGA	3.31	13.15	12.53	9.66	3
SIAT4A	6482	NM_173344	CGAUGG, CGGCAG	7.84	7.6	13.13	9.52	3
CSF3	1440	NM_172219	GCCUU, GGAGGU	3.06	16.37	8.62	9.35	3
LOC389123	389123	NM_203424	GAUGUU, GUACC	5.75	10.46	11.83	9.35	3
POU3F1	5453	NM_002699	GGCAAG, GCACGA	0.18	12.19	15.34	9.24	2
RWDD1	51389	NM_015952	GACUAU, GCAGAA	1.07	12.38	14.15	9.20	2
LOC92154	92154	NM_138383	AACGAG, GGGUAG	1.51	12.03	14.05	9.20	2
DCD	117159	NM_053283	CGUCUU, UCGAA	1.08	13.37	12.94	9.13	2
RPL7	6129	NM_000971	AAUGGA, GGUAAA	2.5	12.12	12.04	8.89	2
PLCD4	84812	NM_032726	CCGACA, GACCAA	1.32	14.1	10.7	8.71	2
NKIRAS1	28512	NM_020345	GGUUUG, GUUCU	-1.46	12.18	15.1	8.61	2
ODZ2	57451	NM_001122679	CGAAUA, CCAUUG	1.43	12.03	12.03	8.50	2
RPL10A	4736	NM_007104	ACGAAA, CCUAUA	2.77	11.58	11.07	8.47	2
C7ORF3	64434	NM_138400	AGCGAA, GGCAA	4.73	9.29	10.92	8.31	3
C6ORF93	84946	NM_032860	GCAGAU, GAAGAA	0.8	15.12	8.84	8.25	2
FLJ39743	283777	NM_182562	CGUUAA, CUAGA	-0.55	10.4	14.8	8.22	2
HEATR1	55127	NM_018072	GAUAUA, ACUAA	3.3	4.54	16.63	8.16	3
EVER2	147138	NM_152468	GGCAUGU, GAACU	2.19	13.45	8.79	8.14	2
NQO2	4835	NM_000904	UCCUUU, GAGGC	2.24	16.61	5.55	8.13	2
EEF2	1938	NM_001961	AUGUAU, GGACAU	2.6	12.04	9.43	8.02	2
NOL5A	10528	NM_006392	GCGGUA, CAGUAU	1.81	13.39	8.85	8.02	2
IQSEC3	440073	NM_015232	GUAUUA, CAAAC	2.9	15.16	5.96	8.01	2
CASKIN2	57513	NM_020753	CGGAAU, CCACAA	3.9	11.62	8.47	8.00	3
IL17D	53342	NM_138284	GCCUAC, GGGCCU	6.47	5.91	11.41	7.93	3
FLJ20359	54905	NM_017781	CGCGAA, GGUUC	3.84	13.95	5.95	7.91	3

RPS5	6193	NM_001009	GGAUUA, GCUCAU	0.45	13.25	9.97	7.89	2
DKFZP434G156	64753	NM_022742	GCGCUU, AAUUAC	1.09	10.52	11.78	7.80	2
C6ORF11	9277	NM_005452	GGAUCU, CCGAAC	1.04	10.52	11.82	7.79	2
PCDHGA3	56112	NM_032011	GCUCAA, GCGAAA	2.95	9.65	10.68	7.76	2
FLJ12519	84128	NM_032168	GCGGCA, GAACAG	0.13	12.51	10.51	7.72	2
CRYGS	1427	NM_017541	CCUGUA, UGACUC	1.67	12.73	8.74	7.71	2
SYNGR4	23546	NM_012451	GGCCAA, UAUGAU	0.37	10.78	11.99	7.71	2
POLR2J3	548644	NM_001097615	GAAGGA, GCGGGU	1.98	12.48	8.65	7.70	2
ARHGEF15	22899	NM_173728	UGAAGA, GGUUAC	4.09	8.89	9.95	7.64	3
BMS1L	9790	NM_014753	GGUUAU, CAAAAC	2.2	11.82	8.9	7.64	2
C16orf50	84229	NM_032269	CAAGUA, GACUUC	0.36	12.36	10.17	7.63	2
CENTB5	116983	NM_030649	UCGUAA, UCAAAG	1.07	7.27	14.25	7.53	2
DDX21	9188	NM_004728	GCAUGU, GUACCU	-0.56	11.85	11.1	7.46	2
SGCG	6445	NM_000231	UACUGU, UGUCAC	0.7	11.25	10.3	7.42	2
RPL38	6169	NM_001035258	AUGAAC, CGGAAA	0.09	12.95	8.99	7.34	2
OR51L1	119682	NM_001004755	UCGUCU, GUUCUA	2.13	6.63	13.15	7.30	2
PSG11	5680	NM_001113410	GCAUAA, ACUCAU	0.06	11.5	10.25	7.27	2
KSR2	283455	NM_173598	UAUUCA, GAGGAG	3.82	6.86	11.06	7.25	3
RPL36	25873	NM_033643	CUAUGG, GCGCCA	0.3	11.06	10.34	7.23	2
DHCR7	1717	NM_001360	UGACUU, GCCCAG	1.56	10.74	9.24	7.18	2
ERBP	30836	NM_014597	UCGCAA, AGAAAC	1.44	8.74	11.36	7.18	2
NFE2L2	4780	NM_006164	UUAAUA, UGGAGU	2.26	6.88	12.24	7.13	2
PIN4	5303	NM_006223	GAGAUU, GCAUUU	0.45	10.9	9.92	7.09	2
RASSF2	9770	NM_170773	AAGUGU, GAGAAC	3.4	7.58	10.26	7.08	3
NAT8L	339983	NM_178557	GUACUA, GUUUG	3.04	10.15	7.66	6.95	3
TMEM43	79188	NM_024334	CGACAA, UUAAAU	3.85	9.44	7.56	6.95	3
AZI1	22994	NM_001009811	CAGUGC, CCUGGA	4.32	8.83	7.51	6.89	3
KIF27	55582	NM_017576	GAAACA, CAGGAC	0.89	7.25	12.43	6.86	2
TRIM75	391714	XM_939332	GUCGUU, GAGACC	0.98	8.1	11.46	6.85	2
LAMR1	3921	NM_001012321	AGCAUA, GGUCAU	-0.16	6.62	13.96	6.81	2
MGC15619	84329	NM_032369	GGUGAA, UAACUA	5.09	7.83	7.35	6.76	3
PWP2H	5822	NM_005049	GGCUAA, CUUCAC	2.67	9.3	8.24	6.74	2
LOC606495	606495	NM_001031672	CGCACU, CAAUGA	3.17	9.02	7.92	6.70	3
AP2B1	163	NM_001282	CCAAGA, GAUGUU	0.79	9.64	9.6	6.68	2

TRIM61	391712	NM_001012414	CUAAUU, UCAGAA	6.1	5.91	7.77	6.59	3
LATS2	26524	NM_014572	CCAAUA, AAUCAGA	6.53	8.35	4.88	6.59	3
GPR147	64106	NM_022146	GCAAGA, GUGAGC	-0.64	9.47	10.61	6.48	2
RNASE9	390443	NM_001001673	CGUGGA, CCACUA	3.5	9.64	6.27	6.47	3
LOC113444	113444	NM_138428	CGAAUA, CGAACA	1.15	8.68	9.56	6.46	2
CIRH1A	84916	NM_032830	UUAUGA, GAACAC	2.34	7.54	9.48	6.45	2
DAP3	7818	NM_004632	UGGUAA, GAGAAC	2.36	8.92	8.07	6.45	2
CDH10	1008	NM_006727	CACAUUU, CAGCU	2.63	9.63	7.08	6.45	2
MIA	8190	NM_006533	GAGAUC, CAAAGU	3.95	8.57	6.82	6.45	3
URG4	55665	NM_017920	GACAUU, GGAGUC	-0.33	7.1	12.53	6.43	2
S100A9	6280	NM_002965	AACAUA, CCAAUA	0.47	10.22	8.55	6.41	2
RPL23	9349	NM_000978	GUAUAU, AGAUAA	-1.03	8.48	11.75	6.40	2
BBX	56987	NM_020235	AACUUG, CCGAUC	1.94	7.81	9.37	6.37	2
ELMO3	79767	NM_024712	CUUCAU, ACAAGG	7.11	5.08	6.88	6.36	3
KIAA1467	57613	NM_020853	GGACUU, CCUGAC	5.45	5.93	7.65	6.34	3
RPS27A	6233	NM_002954	GUAUG, GGGUGU	-1	13.1	6.91	6.34	2
DKFZP564O0463	25879	NM_015420	GCGGAA, CGAAAG	-0.31	9.96	9.3	6.32	2
HNRNPA3	220988	NM_194247	GAGGUG, UAUAAU	4.11	7.11	7.7	6.31	3
DKFZP434K2435	84216	NM_032256	CGACUU, UCAAGC	6.75	6.62	5.44	6.27	3
WBSCR27	155368	NM_152559	AUACAA, CGGACU	3.26	8.06	7.37	6.23	3
DHRSX	207063	NM_145177	GAAGAA, CGAAGC	6.39	6.48	5.81	6.23	3
NBL1	4681	NM_005380	CUAGGA, GCAAGC	0.3	4.35	13.87	6.17	2
FLJ90650	206338	NM_173800	GGCAUU, ACGUGU	5.25	5.57	7.66	6.16	3
ARF3	377	NM_001659	UGAGAAU, GGAAA	-1.15	12.4	7.21	6.15	2
FOXD1	2297	NM_004472	CGAUGC, AAACAC	1.38	8.31	8.58	6.09	2
WBSCR22	114049	NM_017528	GGAUAU, CCGGUG	1.65	8.12	8.48	6.08	2
LOC57821	57821	NM_021179	GAGCAA, AUGAUA	2.63	7.32	8.29	6.08	2
ZNF263	10127	NM_005741	GAGAGA, GCUUAG	2.69	5.93	9.53	6.05	2
SERPINB9	5272	NM_004155	UUGAUG, GGAAUC	2.68	8.1	7.32	6.03	2
RPL17	6139	NM_001035006	GAAAGA, GCUCAG	-1	14.1	4.98	6.03	2
XDH	7498	NM_000379	UGGAAU, UAGAGC	0.29	10.41	7.33	6.01	2
HSFY1	86614	NM_152584	CCUUUA, UCAGAA	3.49	7.86	6.63	5.99	3
RIMS4	140730	NM_182970	UAGCGU, CGUCU	0.8	11.25	5.92	5.99	2
MITF	4286	NM_198158	UUGAUU, AGACG	3.35	7.59	7.02	5.99	3

ELL3	80237	NM_025165	CGGCGA, GCAAGA	2.15	8.87	6.92	5.98	2
PPP2R2A	5520	NM_002717	ACUACA, GCAAGU	1.85	8.49	7.6	5.98	2
GCN1L1	10985	NM_006836	GCGGAA, GCUAUC	0.2	8.42	9.25	5.96	2
MPHOSPH10	10199	NM_005791	UCGGAA, AAACAC	1.3	9.82	6.69	5.94	2
KIAA1751	85452	NM_001080484	AGCGAGA, CCACAU	1.07	8.57	8.15	5.93	2
MAP2K2	5605	NM_030662	AUGGAA, GGUCCG	6.49	6.48	4.81	5.93	3
CASP8	841	NM_033358	AGCAUU, GAUAAU	0.55	6.62	10.56	5.91	2
CYBA	1535	NM_000101	ACCAUG, GUACAU	1.18	3.73	12.81	5.91	2
GAL3ST4	79690	NM_024637	CGGAGU, CCGAGA	2.59	7.32	7.77	5.89	2
DNMT3B	1789	NM_175849	GAAUUA, CGAAAU	4.37	9.96	3.34	5.89	3
PLCD3	113026	NM_133373	GCCACA, CCUAUG	1.16	6.13	10.35	5.88	2
LOC647060	647060	NM_001085452	GGGUAA, GGAGAC	3.08	-0.26	14.69	5.84	2
CDH4	1002	NM_001794	GAUAUC, GCAGUA	1.32	8.19	7.97	5.83	2
EVC	2121	NM_153717	AUAAUA, GCAGGC	1.56	7.32	8.58	5.82	2
RNASEH2A	10535	NM_006397	GGACUU, AUGCAU	0.96	8.79	7.71	5.82	2
TPO	7173	NM_175722	ACAGAA, GUACGU	3.65	7.06	6.73	5.81	3
CES2	8824	NM_198061	UUGUAU, CAUGAA	5.23	7.04	5.12	5.80	3
LOC283130	283130	NM_001077241	GGCAAU, GGUCAG	1.6	7.32	8.45	5.79	2
LOC129401	129401	NM_001008544	CGCUCCA, CUGACA	1.6	7.45	8.22	5.76	2
APOBEC3B	9582	NM_004900	GACACA, UGACCU	2.82	6	8.44	5.75	2
GPR4	2828	NM_005282	AUCGGGU, UACCA	0.8	7.23	9.21	5.75	2
TLR3	7098	NM_003265	UAAUAA, AGACCA	2.61	8.06	6.55	5.74	2
KIAA0251	23042	NM_015027	GUUUAA, UGACAG	0.68	9.15	7.33	5.72	2
OR6B2	389090	NM_001005853	AAGGAC, CGCCAA	1.62	9.19	6.35	5.72	2
HOOK2	29911	NM_001100176	GGACUU, UCACCU	1.78	5.56	9.8	5.71	2
RPL35A	6165	NM_000996	GCUUAU, AGUCAC	-0.02	10.06	7.04	5.69	2
C6ORF154	221424	NM_001012974	CGGCAA, CUGCAC	1.85	7.11	8.11	5.69	2
USP45	85015	XM_371838	CUCUUA, GAAGGC	1.22	4.96	10.89	5.69	2
ESD	2098	NM_001984	GAUCAA, GUAAAU	1.37	7.17	8.52	5.69	2
ECHDC1	55862	NM_018479	GAGCUU, GCAUGU	3.38	10.13	3.53	5.68	3
ZNRD1	30834	NM_014596	ACUUU, GGACCUC	0.48	10.62	5.93	5.68	2
SPIN-2	474343	NM_001006683	GGAAUA, UCACAC	4.1	6.82	6.1	5.67	3
IPO8	10526	NM_006390	GAACGA, GGCCAG	1.69	6.96	8.36	5.67	2
C7ORF16	10842	NM_006658	GUGUUU, UAAGGA	1.86	10.53	4.61	5.67	2

MPP1	4354	NM_002436	UCAUA, UGAGAG	2.02	7.54	7.41	5.66	2
XPO1	7514	NM_003400	ACAAU, UAGAUA	0.49	7.24	9.2	5.64	2
GNPNAT1	64841	NM_198066	AAGUAA, GUGAA	6.37	7.6	2.89	5.62	2
SGEF	26084	NM_015595	UACGAA, AGAGU	3.06	5.34	8.4	5.60	3
CAMK4	814	NM_001744	GACUAC, GGUGC	5.86	5.89	5.04	5.60	3
C10ORF80	159686	NM_001008723	AUUUA, GCGAAU	2.86	6.31	7.61	5.59	2
FLJ45032	643853	NM_001039770	CCACA, GGUGGA	-0.44	6.37	10.85	5.59	2
ZFYVE28	57732	NM_020972	ACGGGC, ACAACA	4.6	6.79	5.33	5.57	3
FLJ21128	80153	NM_025083	JAUGAA, CGUAU	4.6	7.64	4.46	5.57	3
POLD3	10714	NM_006591	GACUA, CAAUUA	3.5	7.74	5.44	5.56	3
FLJ21934	79799	NM_024743	CGACUA, GAGGCA	2.94	7.01	6.62	5.52	2
FBXO23	26262	NM_001006616	GACUUU, GAACC	0.26	9.12	7.19	5.52	2
RABL3	285282	NM_173825	GAAGUA, UGGAA	3.39	7.28	5.89	5.52	3
C20orf118	140711	NM_080628	GAGACA, GUUACA	7.15	3	6.38	5.51	3
NEU1	4758	NM_000434	GAGACA, GAACGA	3.59	5.9	6.97	5.49	3
FLJ30058	158763	NM_144967	CGUUA, GGACGA	-1.04	9.15	8.31	5.47	2
MET	4233	NM_000245	AAUAUA, GAGCCA	4.34	8.22	3.85	5.47	3
SPRR3	6707	NM_005416	GAGCA, GCAGAA	1.92	5.63	8.84	5.46	2
HTATIP	10524	NM_182709	GCAUGA, GGACA	-0.6	5.8	11.17	5.46	2
C20ORF6	51575	NM_016649	GAGCCA, CAGAGG	0.49	6.63	9.13	5.42	2
HARSL	23438	NM_012208	GGACUA, CCUAGU	0.87	9.09	6.27	5.41	2
FERD3L	222894	NM_152898	GAUGU, GGAAGA	4.86	6.7	4.58	5.38	3
ASMT	438	NM_004043	UAAUGA, UGGCA	2.43	7.88	5.82	5.38	2
KRTHA5	3886	NM_002280	UGGACA, CAGCA	0.64	9.79	5.69	5.37	2
RPL18	6141	NM_000979	GGAUAU, GAUCC	-0.15	9.15	7.08	5.36	2
ADAM23	8745	NM_003812	GGUAUA, GCUGU	0.47	5.16	10.44	5.36	2
OMD	4958	NM_005014	UGCACA, GGUGA	2.55	7.44	6.07	5.35	2
DLAT	1737	NM_001931	UGGCAA, GCACAG	2.69	6.73	6.64	5.35	2
ZNF17	7565	NM_006959	GAGAA, GGGAAU	4.15	3.43	8.47	5.35	3
KIAA1811	84446	NM_032430	GAAAUA, GGGGU	2.85	6.11	7.09	5.35	2
GPR146	115330	NM_138445	CAGCGU, UGGCCA	1.99	4.01	10.03	5.34	2
DDX17	10521	NM_030881	JAUGCU, CAAAUG	1.1	8.45	6.47	5.34	2
SLITRK2	84631	NM_032539	GAGCGA, GGUCCU	1.73	7.22	7	5.32	2
C4ORF9	8602	NM_003703	GAAGAA, AAAGAU	0.3	6.29	9.35	5.31	2

MGC17986	163071	NM_153608	ACGGAA, AAUCCU	1.74	9.41	4.76	5.30	2
CLUL1	27098	NM_199167	ACUGGUA, UAUAC	-0.01	9.27	6.64	5.30	2
CNKSR3	154043	NM_173515	GGAAA, GGAUUG	-0.49	8.27	8.11	5.30	2
C22ORF11	26150	NM_015653	GUGAAU, CAAAU	2.51	5.41	7.94	5.29	2
IRF5	3663	NM_032643	CAGGUG, UCAAG	2.46	6.69	6.69	5.28	2
KIAA0690	23223	NM_015179	CCGCAA, UCAGCA	2.13	7.89	5.78	5.27	2
FAM9C	171484	NM_174901	GAGCUA, UGGCA	2.12	3.66	10.02	5.27	2
CRYGA	1418	NM_014617	CGAUUU, UGUAC	2.61	6.59	6.56	5.25	2
CALCB	797	NM_000728	GGUGAU, AAACCU	5.47	6.55	3.72	5.25	3
C9ORF89	84270	NM_032310	CUCUUG, GAUCA	2.64	6.17	6.84	5.22	2
SLC4A11	83959	NM_032034	CGACUA, GUUUCU	1.51	9.4	4.71	5.21	2
SSTR5	6755	NM_001053	CCAAGA, GCGGG	5.05	5.03	5.52	5.20	3
CEP250	11190	NM_001035518	CAACUA, AAGCUG	6.04	4.81	4.74	5.20	3
DKFZP761A132	84256	NM_020912	AGCAA, GCAGCUU	2.65	7.71	5.22	5.19	2
FLJ43752	401253	NM_207497	ACGAGAA, AGACA	3.24	7.48	4.74	5.15	3
LIN9	286826	NM_173083	CGAUUA, ACAGGA	1.41	6.66	7.37	5.15	2
SMAD6	4091	NM_005585	UCAUCA, GUGCGG	0.95	5.82	8.66	5.14	2
C3orf31	132001	NM_138807	UAACUA, AAAUUA	1.9	6.91	6.61	5.14	2
FLJ30656	124801	NM_152344	CGCAAA, GGAAAU	0.59	7.4	7.41	5.13	2
ZNF223	7766	NM_013361	GACAAU, AGUGU	4.68	2.96	7.74	5.13	2
STX19	415117	NM_001001850	GCUCAA, GAACUU	4.59	5.87	4.9	5.12	3
DKFZP434F2021	25871	NM_001025073	UAUUAA, CAGAGA	2.07	6.04	7.25	5.12	2
ATP11C	286410	NM_001010986	ACGCUA, AUGGAA	-0.04	6.37	8.99	5.11	2
CAMK2G	818	NM_001222	GACUUC, GUACA	1.43	7.13	6.75	5.10	2
GPR77	27202	NM_018485	CAGCAA, UGCAGU	1.43	8.45	5.41	5.10	2
RPS12	6206	NM_001016	GGCUCA, GCAAG	-1.83	9.7	7.38	5.08	2
RPL8	6132	NM_000973	GGAGAA, CAUGAA	-0.13	8.46	6.9	5.08	2
BSND	7809	NM_057176	GAUGUU, CAGCG	2.92	6.81	5.48	5.07	2
C6ORF210	57107	NM_020381	UUAGAA, GAAUAU	0.58	5.97	8.65	5.07	2
IFT140	9742	NM_014714	GAGACA, GGUCAA	1.85	8.47	4.87	5.06	2
ANXA2	302	NM_004039	UAUUUA, AAAACC	0.58	6.77	7.84	5.06	2
PGM1	5236	NM_002633	CGAGCAA, CAGUU	1.14	8.36	5.68	5.06	2
HOXB8	3218	NM_024016	CGGCAAA, UGACCA	1.07	7.52	6.55	5.05	2
RPL14	9045	NM_003973	GGUCGA, GCAUAA	-0.47	7.86	7.73	5.04	2

YTHDC2	64848	NM_022828	GUUAAA, CGAAAU	2.13	6.24	6.71	5.03	2
EPB41L4A	64097	NM_022140	GCUACA, AGUAAG	2.05	8.07	4.93	5.02	2
ZNF236	7776	NM_007345	GCGUAA, GAGGAG	2.72	6.59	5.73	5.01	2
MYOD1	4654	NM_002478	UGACGA, GCGCAA	3.6	5.65	5.71	4.99	3
HT014	57095	NM_020362	CCGACA, AAACCU	3.23	5.59	6.14	4.99	3
LPIN1	23175	NM_145693	GAGAAU, CAAAU	1.32	5.26	8.38	4.99	2
DAAM1	23002	NM_014992	UAUUA, GUGGAG	1.14	7.01	6.78	4.98	2
ZC3HDC8	84524	NM_032494	GAUGAU, AGAGAC	3.33	6.16	5.42	4.97	3
TRIM22	10346	NM_006074	CAAGGA, GUAGAU	4.22	3.58	7.08	4.96	3
ADM	133	NM_001124	UUCUAA, CCACUU	4.94	6.5	3.41	4.95	3
NOP5/NOP58	51602	NM_015934	AGAGAA, ACGCAA	3.79	3.36	7.67	4.94	3
DNAJC16	23341	NM_015291	GCGAGA, CGUGAA	2.35	5.48	6.98	4.94	2
LCN7	64129	NM_022164	CACUGA, GAAGGA	1.58	3.62	9.61	4.94	2
FPRL2	2359	NM_002030	AUUCUA, GGUCGU	2.69	6.67	5.42	4.93	2
PRKCZ	5590	NM_001033582	CUAAUC, GACCAA	2.65	4.54	7.59	4.93	2
CAMKK1	84254	NM_172207	GACAGA, GCCCAG	4.44	3.29	7	4.91	3
RAB3GAP1	22930	NM_012233	GGGCUA, CAGCUG	5.74	5	3.97	4.90	3
PPRC1	23082	NM_015062	AUCGUU, GGACUC	3.07	6.67	4.97	4.90	3
CASP9	842	NM_032996	UUAUAA, UCAUAC	0.02	6.05	8.64	4.90	2
CLDN23	137075	NM_194284	CAGCGA, UUAACA	1.63	4.97	8.06	4.89	2
MGC16169	93627	NM_033115	AUGAGU, CAAAGU	5.33	4.16	5.14	4.88	3
ZDHHC2	51201	NM_016353	CAUCUA, ACAAAU	0.63	7.09	6.88	4.87	2
C9ORF41	138199	NM_152420	GCGUAA, AGGAGA	2.68	7.27	4.64	4.86	2
BYSL	705	NM_004053	GGGCUU, GAGCA	2.25	3.81	8.51	4.86	2
PIK3CG	5294	NM_002649	GAGUUC, GAAUUC	1.06	5.38	8.12	4.85	2
FLJ23231	80149	NM_025079	CAGCGU, CAGCACA	4.54	4.19	5.82	4.85	3
FLJ11036	55287	NM_018306	GGACUA, CGGAAA	2.41	5.85	6.26	4.84	2
TNNI3K	51086	NM_015978	CACCUA, GGAAUA	5.14	4.61	4.74	4.83	3
FLJ39575	286006	NM_001134468	UAGGAA, UAACAU	4.94	5.71	3.84	4.83	3
CABIN1	23523	NM_012295	CGACUA, GGAGAA	-1.04	10.61	4.91	4.83	2
RPL11	6135	NM_000975	UGGGAUC, GAACU	-0.78	5.41	9.84	4.82	2
COLEC10	10584	NM_006438	GAGAUA, CCCAAU	6.57	2.7	5.15	4.81	2
CCR9	10803	NM_006641	UUUACA, GCACCA	3.07	4.59	6.72	4.79	3
MFAP3	4238	NM_001135037	UCGUUA, UCUGUA	0.71	8.39	5.23	4.78	2

GTF2H3	2967	NM_001516	AACAUA, AGAAUG	3.26	7.04	4.01	4.77	3
IFRG15	64163	NM_022347	UCCCUA, AGGUAU	0.16	9.8	4.35	4.77	2
BRCC2	414899	NM_001001786	AGGACA, GCUCUA	3.05	4.82	6.42	4.76	3
RPL34	6164	NM_000995	AAGUUA, GUUAAA	0.12	5.89	8.24	4.75	2
C6ORF142	90523	NM_138569	CGAGAA, GAGGAG	0.78	6.4	7.03	4.74	2
BAG2	9532	NM_004282	UGAUGA, CAAUUA	-0.44	4.46	10.19	4.74	2
3'HEXO	90459	NM_153332	CCGAAU, CGGAAG	1.03	6.86	6.3	4.73	2
C18ORF10	25941	NM_015476	GAAUAA, GCAUUA	2.61	6.93	4.6	4.71	2
BRD3	8019	NM_007371	CGAAUU, GGAGAG	2.04	4.84	7.21	4.70	2
ALOX15B	247	NM_001141	CUCCUG, UCUCUG	5.71	5.31	3.06	4.69	3
CABP1	9478	NM_004276	GAGAGU, GGGAAA	1.82	5.48	6.77	4.69	2
FLJ32800	196951	NM_152647	UCGAUA, GGUCAG	0.82	5.4	7.81	4.68	2
OR5D13	390142	NM_001001967	GGCUUA, GCUCCC	3.34	4.58	6.09	4.67	3
OR9I1	219954	NM_001005211	CAAAUA, UCACCA	4.3	5.97	3.73	4.67	3
ZNF342	162979	NM_145288	CCGCCA, GAGCCA	2.13	2.08	9.76	4.66	1
CGI-41	51093	NM_015997	GAGUAC, GCACCC	-0.43	6.72	7.67	4.65	2
DKFZP586L0724	25926	NM_015462	GCGAGA, UGAAGU	1.95	6.57	5.41	4.64	2
CYP11B1	1584	NM_001026213	CCAACG, GCACCCA	7.46	4.36	2.07	4.63	2
HUS1B	135458	NM_148959	ACGACG, CAAAGG	0.58	7.42	5.87	4.62	2
CRP	1401	NM_000567	AACGAA, GGUCUA	0.87	6.64	6.33	4.61	2
PHF23	79142	NM_024297	CAAUUG, GCAAGA	-1.19	3.81	11.2	4.61	2
LOC92270	92270	NM_001017971	ACAAUA, ACGAUU	3.52	4.3	5.99	4.60	3
DDX18	8886	NM_006773	GAAUA, GAUAAU	0.43	2.56	10.81	4.60	1
ME1	4199	NM_002395	UAUAUA, GAGUAA	7.01	1.87	4.91	4.60	2
TUWD12	282809	NM_172240	GCGCUU, CGACAU	2.88	3.04	7.87	4.60	2
WFDC2	10406	NM_006103	CGGACA, AUGAAA	3.15	6.07	4.53	4.58	3
C6ORF10	10665	NM_006781	GGUAAA, GACAGG	4.15	4.44	5.14	4.58	3
LOC129138	129138	NM_138797	GAGAUG, AGUUAA	5.34	3.78	4.58	4.57	3
FLJ32871	146279	NM_144674	GCUAUA, CCAAGU	1.98	6.79	4.9	4.56	2
SCD	6319	NM_005063	CUGAUCA, GCACA	-0.69	5.86	8.5	4.56	2
KIN	22944	NM_012311	CCUAGA, GAACAG	6.12	3.02	4.52	4.55	3
FLJ12448	64897	NM_022895	CCUUAA, ACAAGA	1.27	6	6.35	4.54	2
RBM24	221662	NM_153020	CGUGAA, AUGAAA	0.29	4.85	8.43	4.52	2
NR2E3	10002	NM_014249	GGUUCC, GAAGGA	3.35	4.66	5.54	4.52	3

FGF5	2250	NM_033143	UGUUA, GCAGAG	1.51	5.83	6.21	4.52	2
C9ORF67	84814	NM_032728	CGUAAA, UGGUC	3.79	5.18	4.56	4.51	3
TFG	10342	NM_001007565	GUAGGA, GUGAA	3.73	4.72	5.07	4.51	3
REL	5966	NM_002908	GUGUAA, AAAGA	0.06	6.43	7.03	4.51	2
GTF2H2	2966	NM_001515	CUUUAA, GAGAA	6.78	3.61	3.12	4.50	3
MASS1	84059	NM_032119	AUAAA, GGAAUU	3.69	4.41	5.4	4.50	3
AMELY	266	NM_001143	CAGGAA, GGAUG	6.36	4.15	2.97	4.49	2
RPS11	6205	NM_001015	GGGCUU, GGGAC	-0.4	4.44	9.43	4.49	2
RGS2	5997	NM_002923	GACCCA, GAAUA	3.68	5.01	4.76	4.48	3
MIS12	79003	NM_024039	UGAAA, GACGUU	3.78	3.59	6.07	4.48	3
USP21	27005	NM_012475	UUUGC, CGAGAG	1.86	5.79	5.78	4.48	2
OR13C9	286362	NM_001001956	GAUUCA, CAGGA	6.06	4.39	2.96	4.47	2
SKB1	10419	NM_006109	AUGAUU, CGAAA	3.53	6.47	3.4	4.47	3
TCEAL8	90843	NM_001006684	AAGCCA, CCAGA	6.37	5.81	1.21	4.46	2
C9ORF10OS	158293	NM_198841	AAGCUA, GGACA	4.04	4.8	4.51	4.45	3
SPRED1	161742	NM_152594	CCCAAA, AGAAU	1.32	8.09	3.94	4.45	2
CNTN3	5067	NM_020872	AUUUG, CGUCU	6.1	3.85	3.39	4.45	3
OLFM1	10439	NM_006334	UCAUA, GACCAU	2.07	5.86	5.41	4.45	2
COPG2	26958	NM_012133	AUCGA, CCAUGU	0.11	8.08	5.11	4.43	2
AKAP3	10566	NM_006422	CAAUGU, GGGCU	2.24	6.18	4.86	4.43	2
ZNF383	163087	NM_152604	GGCAU, CUGGAA	0.29	8.42	4.57	4.43	2
LPA	4018	NM_005577	CAUUAU, GGAGG	-0.67	7.14	6.79	4.42	2
AIF1	199	NM_032955	AGCUA, GAAAUU	2.22	5.92	5.1	4.41	2
REPRIMO	56475	NM_019845	GCGGU, UGAUC	-0.75	6.93	7.05	4.41	2
GNB3	2784	NM_002075	CUCUCC, UGACU	2.3	4.97	5.95	4.41	2
RPUSD4	84881	NM_032795	CGGUCU, CUUGG	0.82	5.42	6.97	4.40	2
CGI-116	51019	NM_016053	AAGAUA, UCUCAU	-0.53	4.26	9.47	4.40	2
FSCN1	6624	NM_003088	GCAAA, GAGCAU	2.29	4.41	6.49	4.40	2
C6ORF149	57128	NM_020408	CGUUU, UGUCA	3.16	7.38	2.65	4.40	2
ALS2CR8	79800	NM_024744	GUGUAA, AAAGA	1.86	4.05	7.27	4.39	2
PRAP1	118471	NM_145202	AGUCAA, GUCCA	4.17	4.02	4.96	4.38	3
RPS19	6223	NM_001022	GAACCA, UCAGA	-1.61	6.42	8.3	4.37	2
DAB1	1600	NM_021080	GAUUCC, GCCAA	2.38	4.71	6.01	4.37	2
C15ORF17	57184	NM_020447	GAGCUC, GCAUG	-0.61	7.86	5.84	4.36	2

KA36	125115	NM_182497	GAAUU, GCCCGG	1.34	4.64	7.1	4.36	2
ITGA7	3679	NM_002206	JACUUA, UAUGAU	3.25	5.41	4.39	4.35	3
FLJ37078	222183	NM_001110199	GAAGCU, CAAAGA	1.71	5.2	6.14	4.35	2
EPRS	2058	NM_004446	JAAAUC, GGUGAG	-1.15	5.09	9.09	4.34	2
CYP4F11	57834	NM_021187	UGACUU, GGUCUG	5.67	2.6	4.73	4.33	2
ALCAM	214	NM_001627	AUUGU, GAUGGU	4.2	5.23	3.56	4.33	3
FLJ39441	144108	NM_194285	CACGAA, GAGAAU	0.61	6.07	6.3	4.33	2
WHSC2	7469	NM_005663	UGCGGA, UGACCA	5.22	3.05	4.7	4.32	3
TEX11	56159	NM_031276	CGGAAA, AGAACA	4.75	3.27	4.93	4.32	3
P8	26471	NM_012385	CAAGAG, CAGGGU	1.01	6.35	5.58	4.31	2
FLJ22938	79729	NM_024676	CCGACA, AGACAU	0.06	4.03	8.84	4.31	2
BRI3BP	140707	NM_080626	JCGUGGA, GCGUG	1.06	5.59	6.24	4.30	2
C2ORF25	27249	NM_015702	UCGUCA, GAUGAA	3.04	3.27	6.54	4.28	3
FLJ33977	285605	NM_173666	ACAUUA, UCUCAG	1.88	5.42	5.54	4.28	2
GPR19	2842	NM_006143	UACUAU, AAACUA	3.63	4.75	4.43	4.27	3
DEFB124	245937	NM_001037500	JUGAGUA, GCAAG	-0.46	9.18	4.08	4.27	2
FUCA1	2517	NM_000147	GACUA, GGAUUU	1.86	7.38	3.54	4.26	2
FLJ14525	84886	NM_032800	CUUACU, GAAGCG	4.6	4.46	3.7	4.25	3
DPP9	91039	NM_139159	CUCGUA, GAACAA	1.15	6.42	5.18	4.25	2
CASP1	834	NM_033295	AGAUGC, CCGCAA	0.21	5.42	7.11	4.25	2
ZNF408	79797	NM_024741	GCAUUU, UCGCCA	3.01	4.27	5.45	4.24	3
MUC7	4589	NM_152291	ACGGAGA, GAUCA	2.47	5.95	4.28	4.23	2
CHN1	1123	NM_001025201	CGCCAU, CCGGAU	0.52	6.49	5.67	4.23	2
SNRK	54861	NM_017719	ACAUUA, GAAGUG	3.17	4.75	4.75	4.22	3
FLJ20259	54870	NM_198880	UAUACA, CGGAGU	3.87	4.49	4.3	4.22	3
DBH	1621	NM_000787	CACUAC, ACGUAC	-0.29	4.41	8.54	4.22	2
ORAOV1	220064	NM_153451	GGACUU, CUUUGA	2.06	4.41	6.18	4.22	2
ZNF18	7566	NM_144680	GACAAA, AGACAU	4.94	6.19	1.5	4.21	2
TIPARP	25976	NM_015508	JAAUAA, GAAAGA	4.21	3.31	5.12	4.21	3
DRIM	27340	NM_014503	CUGUUU, AGAAUU	3.38	6.33	2.92	4.21	2
ZNF579	163033	NM_152600	CGACUU, CCUCGC	1.56	5.05	6	4.20	2
LOC128710	128710	NM_001009608	GGAGAA, AAAGCA	1	5.41	6.17	4.19	2
FLJ23751	92370	NM_001037172	ACGUGA, AUGAG	3.61	4.44	4.48	4.18	3
RPL21	6144	NM_000982	ACUAAA, CAAGUG	-1.52	7.12	6.93	4.18	2

SEMA5B	54437	NM_001031702	CGACUA, AGAACG	4.74	2.27	5.51	4.17	2
ABCG1	9619	NM_207629	CACAUU, GGAAAU	0.86	6.13	5.52	4.17	2
CKLFSF7	112616	NM_181472	GCCUUU, GAAAGU	3.53	6.46	2.51	4.17	2
ACLY	47	NM_198830	UAAACA, GAGAGC	2.7	4.65	5.15	4.17	2
ODZ1	10178	NM_014253	GAGCUU, GGAGCA	1.79	6.09	4.62	4.17	2
NAT5	51126	NM_181528	GAGGCA, UGUAU	1	4.69	6.8	4.16	2
MGAM	8972	NM_004668	ACUUAA, GGACAU	7.52	1.96	3	4.16	2
NAP1L3	4675	NM_004538	GGAUAG, GGGCAG	-0.35	3.46	9.37	4.16	2
GC20	10289	NM_005875	CGACUU, GGUAUC	4.57	3.59	4.31	4.16	3
OR10X1	128367	NM_001004477	UGGGUA, AGUCAC	1.11	8.33	3.03	4.16	2
FLJ22573	79713	NM_024660	GACUCA, ACACCU	1.56	6.3	4.6	4.15	2
ARHGEF7	8874	NM_003899	GGAACA, GGACGA	2.79	5.04	4.62	4.15	2
SIP1	8487	NM_001009183	AGUCAC, GAGCGG	1.52	4.93	6	4.15	2
FLJ23577	79925	NM_144722	GAUCGA, ACACAU	4.28	3.73	4.42	4.14	3
MAGEH1	28986	NM_014061	GAUUUA, GUUCGU	2.91	5.52	3.99	4.14	2
PPP6C	5537	NM_002721	CAAGUA, GUUUGC	0.74	5.62	6.05	4.14	2
C18ORF4	92126	NM_032160	CGUUAA, GAAUGU	1.11	4.78	6.51	4.13	2
PRSS2	5645	NM_002770	CAAUUC, AAUCUA	5.09	3.57	3.72	4.13	3
LOC92345	92345	NM_001128931	GUAUUA, UCAGA	0.66	4.45	7.27	4.13	2
INHBC	3626	NM_005538	GACCUUG, CCACAU	-0.46	5.66	7.18	4.13	2
ZNF451	26036	NM_001031623	UGAUU, CCGUCU	3.69	4.49	4.19	4.12	3
RREB1	6239	NM_001003698	GACAUA, GGGCAC	1.87	4.16	6.33	4.12	2
LASS2	29956	NM_013384	CCGAUU, CAUCCG	1.34	3.77	7.25	4.12	2
C9ORF115	138428	NM_001002913	UGUUU, CAAGGC	1.35	4.81	6.19	4.12	2
DDX5	1655	NM_004396	CCUUGA, GCAUGU	3.81	3.15	5.39	4.12	3
LOC83693	83693	NM_031463	UGCACA, ACGUGA	2.38	4.1	5.86	4.11	2
ACTA1	58	NM_001100	GCAGAA, CGGUAU	2.4	5.19	4.74	4.11	2
SOX6	55553	NM_033326	CCGAAA, ACAUGG	6.05	2.5	3.77	4.11	2
TAP2	6891	NM_018833	GCCAUU, GGAACA	5.3	3.73	3.29	4.11	3
NUP88	4927	NM_002532	GCGUAU, GAACAC	2.03	7.23	3.05	4.10	2
APEH	327	NM_001640	GCAUUA, GGACAA	1.33	6.08	4.89	4.10	2
SRP72	6731	NM_006947	AGACAA, CUACAU	4.31	4.78	3.2	4.10	3
ZCCHC9	84240	NM_001131036	CCGCAA, GAUGGU	1.51	2.31	8.47	4.10	1
CTSW	1521	NM_001335	GGGAUU, GAAGGC	1.28	5.3	5.71	4.10	2

CRX	1406	NM_000554	CUAUUU, GUGAG	2.53	4.75	5	4.09	2
MINA	84864	NM_153182	GAUUAA, GUACAU	3.77	7.21	1.29	4.09	2
CGREF1	10669	NM_006569	GUCUAA, GCACAU	2.73	4.72	4.82	4.09	2
PANX1	24145	NM_015368	AGGUAU, CAUAUU	3.07	2.83	6.36	4.09	2
GAP43	2596	NM_002045	ACCAAA, UAGCUU	2.57	3.33	6.36	4.09	2
HSPG2	3339	NM_005529	GAGCUA, CCGCAG	1.56	5.25	5.44	4.08	2
NR0B2	8431	NM_021969	CUUCUU, CGUAG	2.29	5.73	4.22	4.08	2
RFC3	5983	NM_181558	GAGUAG, CCUUGG	3.16	5.24	3.83	4.08	3
TNXB	7148	NM_032470	CACUUG, GCGGAC	0.12	6.19	5.91	4.07	2
NURIT	220082	NM_152719	GCGUCC, UCGAGC	3.22	6.11	2.85	4.06	2
SMARCC1	6599	NM_003074	CUCCAA, GAACAU	3.95	4.16	4.02	4.04	3
IL11	3589	NM_000641	CGCCUG, GCACAC	2.88	4.65	4.6	4.04	2
LOC93109	93109	NM_001011655	CGGCUA, GUUCAG	1.98	6.56	3.57	4.04	2
LOC120237	120237	NM_001029865	GGAACU, CCGGGA	1.97	5.02	5.12	4.04	2
TNIP3	79931	NM_024873	GAAUUA, ACACAA	2.4	3.85	5.85	4.03	2
CEACAM3	1084	NM_001815	GCGUGA, CUUGGA	3.64	3.26	5.17	4.02	3
NEURL2	140825	NM_080749	CGCAUU, CAAAGA	2.61	5.06	4.39	4.02	2
FLJ31882	146723	NM_152460	AGCUAAC, AUAAAC	1.29	5.55	5.22	4.02	2
TRAM1L1	133022	NM_152402	GUAACA, GAGUAG	1.06	4.2	6.8	4.02	2
ETAA16	54465	NM_019002	CGGAUA, GAGAAU	1.17	5.76	5.11	4.01	2
GRK4	2868	NM_001004057	CCCAUAA, GGGACA	2.38	4.89	4.76	4.01	2
DPH2L2	1802	NM_001039589	GCGAGU, CGACAG	3.37	1.83	6.82	4.01	2
HSPC051	29796	NM_013387	CAGCAU, AGUAUC	2.27	6.1	3.64	4.00	2
AUTS2	26053	NM_015570	AGUAAA, GAAAGC	0.42	6.46	5.13	4.00	2
CTSL	1514	NM_145918	UAACAA, GCAGUG	-0.27	5.47	6.81	4.00	2
MGC48986	161753	NM_175881	UCAUGA, UGGCCA	4.76	5.23	2.01	4.00	2
VRK1	7443	NM_003384	GCAUGA, GGCUUU	2.92	5.85	3.23	4.00	2
MGC13017	91368	NM_080656	JAAUGAA, GCCAGA	-0.57	5.38	7.18	4.00	2
RGS12	6002	NM_198227	GCACGU, CAACUC	2.87	3.3	5.81	3.99	2
IL18RAP	8807	NM_003853	GAAUCG, GGAACC	2.41	8.14	1.43	3.99	1
USP47	55031	NM_017944	GGAUAA, GCUGUC	1.25	4.34	6.38	3.99	2
MRPL41	64975	NM_032477	GGAGUU, UCAAGC	1.38	4.03	6.51	3.97	2
CBLB	868	NM_170662	AGCAAA, GGUCGA	1.3	4.37	6.25	3.97	2
DKFZP564N2472	285877	NM_182595	GAACUU, CUGGGA	4.79	3.9	3.2	3.96	3

ZDHHC16	84287	NM_198045	CUUCUA, CAACGU	0.14	7.27	4.48	3.96	2
MGC40222	221718	NM_152738	GAAACA, CUGCGA	4.99	3.33	3.55	3.96	3
COL17A1	1308	NM_130778	GCGUUU, CAAGCC	2.79	4.51	4.56	3.95	2
OVCH1	341350	NM_183378	CGGUGA, ACGAGG	0.79	4.55	6.52	3.95	2
NXF3	56000	NM_022052	CGGACA, GCAUCC	0.57	4.3	6.99	3.95	2
HMOX1	3162	NM_002133	JGGUGG, CAGUUC	8.52	1.3	2.03	3.95	1
FLJ44186	346689	NM_198508	CGAGAA, CAUCCG	3.63	4.7	3.51	3.95	3
C6ORF111	25957	NM_015491	CCGCAA, UGAAAG	0.69	4.72	6.42	3.94	2
PGRMC1	10857	NM_006667	CUACAA, UGAGUA	1.06	5.51	5.23	3.93	2
LOC649055	649055	XM_938138	GCGAAA, GUCGAA	2.21	5.72	3.82	3.92	2
TRIM16	10626	NM_006470	GUAAAA, GGAACA	2.66	5.24	3.82	3.91	2
PAPD5	64282	NM_022447	UUAUU, GAUAAA	0.37	5.25	6.08	3.90	2
FLJ20718	55027	NM_182922	GAUUCA, CCAAUG	2.89	3.77	5.01	3.89	2
PRO1853	55471	NM_001083946	GGAUA, GCUACU	2.61	3.26	5.77	3.88	2
HDLBP	3069	NM_203346	ACGUUA, CAACAU	2.33	6.6	2.69	3.87	1
RPL7A	6130	NM_000972	CACCGU, AAAUGU	-1.55	7.33	5.83	3.87	2
ACADL	33	NM_001608	JAAUUG, GGUAU	1.11	5.51	4.98	3.87	2
MUTED	63915	NM_201280	GAGUAA, GGCUUA	4.25	1.31	6.02	3.86	2
CAD	790	NM_004341	CAAUUC, GAACCA	4.2	5.36	2.02	3.86	2
LIPA	3988	NM_000235	UGUAUA, CAAAU	1.29	4.84	5.44	3.86	2
CUL2	8453	NM_003591	AUACUA, GCAGA	1.4	3.91	6.25	3.85	2
MFTC	81034	NM_030780	GACCGA, GGGACU	1.23	5.15	5.18	3.85	2
UNG	7374	NM_080911	CUUGUU, GAAGCC	4.37	3.13	4.05	3.85	3
DLX5	1749	NM_005221	GACUAU, CAACUU	3.69	2.91	4.95	3.85	2
OTUB1	55611	NM_017670	UCUAUC, GACGGA	2.01	4.94	4.57	3.84	2
LOC51693	51693	NM_016209	JGAUAA, UCUCUC	0.09	5.32	6.11	3.84	2
BAI3	577	NM_001704	GAUAUA, GAUGGC	2.68	4.11	4.72	3.84	2
GGA3	23163	NM_014001	GGGCUU, UGUGA	2.27	5.61	3.62	3.83	2
GPR158L1	440435	NM_001004334	JUAUUA, GGAGAC	0.73	4.63	6.14	3.83	2
MDA5	64135	NM_022168	JCUUUA, UGACAC	0.16	7.08	4.25	3.83	2
KCTD5	54442	NM_018992	AUUUU, GAACGA	7	2.64	1.84	3.83	1
CHD7	55636	NM_017780	JUGAAA, CCAUGA	-0.21	7.9	3.79	3.83	2
KAAG1	353219	NM_181337	GACGAA, CGGAGU	0.54	7.2	3.73	3.82	2
UBE2F	140739	NM_080678	ACGUUA, CAAUAA	2.03	5.88	3.55	3.82	2

MGC20806	201255	NM_144999	GCGAGA, GUUUG	0.59	5.88	4.99	3.82	2
DBI	1622	NM_020548	UGGAUU, CAACAA	0.33	5.23	5.88	3.81	2
HSD17B6	8630	NM_003725	GGAAU, GCUGUU	2.38	4.54	4.51	3.81	2
BCL6B	255877	NM_181844	CCACUA, GCAGUG	0.09	5.58	5.74	3.80	2
LRRC18	474354	NM_001006939	GAGAAA, AAGCCA	2.19	-0.97	10.17	3.80	1
SLC35C2	51006	NM_173073	CGUUGC, GGCAU	2.19	5.76	3.44	3.80	2
FLJ13072	91646	NM_001110822	CGGUGA, AGAUCA	3.29	4.01	4.07	3.79	3
ZNF490	57474	NM_020714	UGGAAA, UGAGA	2.11	5.13	4.1	3.78	2
ENDOG	2021	NM_004435	UGCACA, GAUGG	0.42	6.34	4.58	3.78	2
GNAT1	2779	NM_000172	GGACUA, GCACG	2.18	1.1	8.05	3.78	1
AMACR	23600	NM_203382	UGGAUG, GAUAU	3.53	5.39	2.41	3.78	2
LOC124751	124751	NM_213597	GACGUU, GCCAA	3.08	3.85	4.4	3.78	3
NT5C2	22978	NM_012229	GCGUUA, CUUGGU	0.61	5.8	4.9	3.77	2
ZNF384	171017	NM_133476	CCUUAU, CAUUU	2.38	5.01	3.92	3.77	2
IRF7	3665	NM_004030	GCACUG, GCGCG	0.67	5.34	5.3	3.77	2
EED	8726	NM_003797	UGUAU, GGAUCU	4.48	4.49	2.32	3.76	2
OR1A2	26189	NM_012352	UCAAA, GUUCU	3.97	4	3.32	3.76	3
PCDHB10	56126	NM_018930	UCCAAA, GGAAAG	4.23	2.94	4.12	3.76	2
KCTD8	386617	NM_198353	GGACA, GCGUGU	1.84	4.18	5.26	3.76	2
PCDHB1	29930	NM_013340	GUAUCA, GGGUG	2.77	2.29	6.21	3.76	1
WBP5	51186	NM_001006614	AGCAAU, ACUUAU	-0.02	5.66	5.63	3.76	2
PYCR1	5831	NM_153824	UGGCUG, GCCCA	-1.29	5.12	7.43	3.75	2
TEX261	113419	NM_144582	GGUUGU, CAUCCU	1.97	2.53	6.73	3.74	1
FLJ20013	54784	NM_017621	AAGGGC, CAGGA	1.28	3.41	6.52	3.74	2
KIAA1836	200014	NM_032449	AGUAUC, GCCAA	3.56	2.85	4.77	3.73	2
ZW10	9183	NM_004724	CUAGAA, CGUAU	8.09	3.21	-0.14	3.72	2
DUX1	26584	NM_012146	GAAUGA, GGAGA	4.75	4.24	2.17	3.72	2
RPL5	6125	NM_000969	GCGUAU, UAGAA	1.56	4.37	5.22	3.72	2
DSG2	1829	NM_001943	CAAGAA, CCUUA	4.85	2.88	3.4	3.71	2
MKNK2	2872	NM_017572	GAACUU, AGGCU	1.02	4.91	5.2	3.71	2
ARID5B	84159	NM_032199	GGACUA, UAAGC	4.87	3.41	2.8	3.69	2
C2ORF22	130814	NM_152391	GGUAA, CCUCAC	3.29	4.65	3.14	3.69	3
KRTAP17-1	83902	NM_031964	GGGUGA, CACCUA	0.62	5.65	4.81	3.69	2
SBBI54	126119	NM_138334	GUCAAU, UGGAC	3.18	3.58	4.31	3.69	3

NUMBL	9253	NM_004756	GAGAUU, CCGAGU	1.38	5.73	3.95	3.69	2
FAM32A	26017	NM_014077	GCGGAA, GUUAUU	0.61	3.63	6.81	3.68	2
MAP1LC3B	81631	NM_022818	CACAUA, GGGUAG	-1.81	7.73	5.13	3.68	2
SLC30A3	7781	NM_003459	GUCGGU, CCGAGA	0.49	3.38	7.16	3.68	2
LEPRE1	64175	NM_022356	GGAGAA, GAGUGA	0.2	6.34	4.49	3.68	2
ADRA1A	148	NM_033304	GUUUGU, GACCAA	1.04	3.83	6.15	3.67	2
HRPT2	79577	NM_024529	AGAUUA, GGAUGU	-0.42	6.6	4.84	3.67	2
MGC3207	84245	NM_032285	GAGAUC, GAUCAU	2.06	4.27	4.68	3.67	2
ZFH4	79776	NM_024721	AUUUA, GAACGA	0.46	6.37	4.18	3.67	2
ADRA1D	146	NM_000678	CGACUA, GACGAG	0.04	5.12	5.83	3.66	2
ITGA8	8516	NM_003638	AGUUAA, GCAUAC	4.38	2.9	3.7	3.66	2
ESAM	90952	NM_138961	UGGGAUC, CAUCA	1.15	5.07	4.76	3.66	2
DUSP7	1849	NM_001947	AAGUAU, CAACG	0.47	4.31	6.19	3.66	2
FLJ32745	165055	NM_144978	UCGUUA, GAGCA	4.7	2.63	3.63	3.65	2
MGC26979	91147	NM_153704	GAGAAA, CCUUAA	2.2	3.55	5.21	3.65	2
RNF39	80352	NM_170770	UUAAUA, GUGGAC	1.73	3.68	5.54	3.65	2
CDON	50937	NM_016952	UAUUAC, GAUCUU	0.77	4.19	5.98	3.65	2
C21ORF6	10069	NM_016940	AACGGA, GGAGU	0.66	6.25	4.04	3.65	2
UFD1L	7353	NM_001035247	UCAUCA, UGACAU	-0.16	5.64	5.48	3.65	2
POPDC3	64208	NM_022361	CGGAAU, GCAGAA	-0.2	6.1	5.05	3.65	2
MIA2	117153	NM_054024	CGAAGA, GGAUAC	0.6	3.79	6.55	3.65	2
RYK	6259	NM_002958	GUAAUA, CCAUGC	3.19	2.15	5.59	3.64	2
PCDH21	92211	NM_033100	CGAGAA, GGGCUG	1.18	3.81	5.94	3.64	2
CDX2	1045	NM_001265	CACGUG, UCACUA	0.93	4.61	5.39	3.64	2
STMN4	81551	NM_030795	GCGUCA, GAAUAA	-0.36	6.54	4.75	3.64	2
IL22RA2	116379	NM_181310	UAUUA, GGAGGC	3.98	3.45	3.47	3.63	3
SLC35C1	55343	NM_018389	GACUUU, UGAGGC	-0.13	6.69	4.33	3.63	2
KIAA1632	57724	NM_020964	UGAUA, AAACAA	1.26	4.57	5.05	3.63	2
CXORF1	9142	NM_004709	UAGUAA, GCUUUA	4.49	2.89	3.48	3.62	2
MUS81	80198	NM_025128	CGUCUA, UGACCC	3.1	4.07	3.69	3.62	3
SCRN2	90507	NM_138355	GUCGAA, CAGGG	2.62	3.63	4.61	3.62	2
GPR174	84636	NM_032553	AUAUAU, CACUGA	1.49	5.59	3.78	3.62	2
SPARC	6678	NM_003118	GACUCU, GAGAAG	4.6	3.45	2.79	3.61	2
NAV3	89795	NM_014903	GAUGUA, CAGGGA	5.28	3.19	2.36	3.61	2

ICA1	3382	NM_001136020	CGAAUU, GGACUU	2.33	4.41	4.09	3.61	2
ARHGDIA	396	NM_004309	GGAAAG, CCGGGU	0.43	7.37	3.03	3.61	2
OBFC1	79991	NM_024928	CUACUA, CAUACA	0.32	4.91	5.6	3.61	2
GANC	2595	NM_198141	GGAUGU, UGGCUC	3.06	4.53	3.22	3.60	3
GOLPH3	64083	NM_022130	GACGUA, GCGGCA	0.69	4.89	5.23	3.60	2
LAMB3	3914	NM_000228	GUAAUC, UCCGAC	2.92	2.81	5.07	3.60	1
IGF2AS	51214	NM_016412	GCCAAA, GAUCGA	0.16	3.46	7.18	3.60	2
PTTG2	10744	NM_006607	GAAAUU, AAAGA	0.58	5.72	4.49	3.60	2
ATG4B	23192	NM_013325	ACAUACA, UCGACA	1	2.24	7.54	3.59	1
POP7	10248	NM_005837	AGGAAA, ACAUGA	1.1	4.02	5.63	3.58	2
PACS1	55690	NM_018026	CGUCAA, CGCCAA	5.35	4.31	1.08	3.58	2
FLJ36004	160492	NM_152590	AAGCUA, AGACAA	2.97	3.47	4.3	3.58	2
HS6ST2	90161	NM_147175	CGACUA, CCCAAU	0.61	6.28	3.85	3.58	2
UNC119	9094	NM_005148	AGGUUU, GCACUC	1.39	4.66	4.68	3.58	2
LOC150159	150159	NM_001100874	GGGAUU, GAGUU	0.62	5.15	4.96	3.58	2
RPL4	6124	NM_000968	GAAUUA, AGUGCU	0.08	5.67	4.98	3.58	2
MT	27349	NM_014507	GAGAAA, ACCAGG	0.46	4.38	5.88	3.57	2
FBXO16	157574	NM_172366	UACAUC, GACCGG	4.43	2.85	3.41	3.56	2
USP8	9101	NM_005154	CAAGUA, CUUCGU	2.48	3.1	5.1	3.56	2
KIAA1221	84146	NM_032186	GUUUAU, CAUGCU	0.84	2.78	7.06	3.56	1
EPN2	22905	NM_148921	GGGACA, CCUUUC	0.08	4.07	6.52	3.56	2
C20ORF175	140876	NM_080829	GGAGUU, CGACUG	5.25	2.35	3.06	3.55	2
SLC35E3	55508	NM_018656	CGGACA, GCUGAU	0.9	4.54	5.22	3.55	2
ITLN1	55600	NM_017625	GGGAUU, GGAAA	1.65	4.26	4.74	3.55	2
LOC152485	152485	NM_178835	GUCUAA, CCAGAG	-0.28	5.23	5.7	3.55	2
PAPD4	167153	NM_173797	GAUGAA, GAAAG	4.24	4.22	2.18	3.55	2
ENPP1	5167	NM_006208	UUAUUA, AAAGUA	2.45	4.41	3.78	3.55	2
KCNH4	23415	NM_012285	UAAUAA, CAUCGA	2.06	5.04	3.53	3.54	2
H2AFZ	3015	NM_002106	AUAGUAA, CAACCA	2.3	4.22	4.09	3.54	2
STXBP6	29091	NM_014178	GAGCGA, AACUGG	1.07	3.25	6.29	3.54	2
OR52W1	120787	NM_001005178	GGGUAU, UGAUC	1.06	3.87	5.68	3.54	2
SERAC1	84947	NM_032861	ACGAGA, CAUAGU	0.81	4.2	5.6	3.54	2
ADH1C	126	NM_000669	UACCGU, CUGGAU	1.81	5.55	3.24	3.53	2
CXCL9	4283	NM_002416	GUCAAA, GAAUGG	1.1	3.47	6.02	3.53	2

EHD4	30844	NM_139265	CAUUCA, AGAUCA	0.62	3.48	6.49	3.53	2
C6ORF81	221481	NM_145028	CUACAA, CCAUCA	3.16	2.43	4.99	3.53	2
MGC29671	201305	NM_182538	GAGAGA, UAAAG	1.73	4.35	4.5	3.53	2
SMPD3	55512	NM_018667	ACCAUA, GCGAGC	3.82	3.8	2.96	3.53	2
IRS2	8660	NM_003749	CAUCGA, CUGCCU	2.22	2.69	5.66	3.52	1
KRTCAP3	200634	NM_173853	AGCCAA, CGGGAU	1.87	4.06	4.61	3.51	2
HIP-55	28988	NM_001014436	CACAUU, ACACAG	1.08	5.17	4.28	3.51	2
EIF3S10	8661	NM_003750	UUCGUA, GAAGA	3.53	3.16	3.84	3.51	3
BRMS1	25855	NM_001024958	GAGAUG, GGAAGC	1.27	5.52	3.72	3.50	2
LOC113730	113730	NM_138433	GCGAGU, AAAUGA	-0.7	7.43	3.76	3.50	2
EBF2	64641	XM_944277	UGCGAAA, CAGAA	1.29	4.97	4.22	3.49	2
DKFZP434M0331	55592	NM_017600	UAAAUA, UGGCGU	0.56	4.85	5.05	3.49	2
DPYSL5	56896	NM_020134	CGUCUA, CUGUAC	5.87	1.88	2.7	3.48	1
KLRA1	10748	NM_006611	UGAUAC, GAAGGC	2.09	6.01	2.35	3.48	1
LOC730394	730394	NM_001042490	GAAACU, UGGACA	0.7	3.35	6.4	3.48	2
KIAA0802	23255	NM_015210	GGGCAA, GGAUG	-0.42	5.72	5.15	3.48	2
SHANK1	50944	NM_016148	GAGUUU, GGGUUC	0.41	6	4.03	3.48	2
RPL13	6137	NM_000977	GGAGAA, UCAGAG	-0.2	7.29	3.34	3.48	2
DRG1	4733	NM_004147	ACGUUG, CAAGAC	3.67	3.16	3.58	3.47	3
FLJ25449	151649	XM_001717727	GGAAAU, AGCAAU	1.04	3.49	5.88	3.47	2
ASAM	79827	NM_024769	GAAUUA, UUAUCA	4.08	1.91	4.41	3.47	2
CXCL6	6372	NM_002993	AAACUG, GCACUU	0.7	6.54	3.16	3.47	2
LOC389792	389792	NM_203434	CCGCAA, AUUGUA	2.81	2.72	4.86	3.46	1
SAA4	6291	NM_006512	GUUCGU, GCAAAC	-0.01	5.16	5.23	3.46	2
KBTBD5	131377	NM_152393	GUAAUU, AAUGAC	2.77	4.34	3.27	3.46	2
PROC	5624	NM_000312	CGGUGA, GCAGUC	2.29	2.78	5.31	3.46	1
TK1	7083	NM_003258	CACUGU, GCACAC	1.45	4.18	4.75	3.46	2
LOC162967	162967	NM_207333	GAGCUU, GCGAAG	1.8	2.88	5.67	3.45	1
CHCHD6	84303	NM_032343	GUGACA, CGUAUU	3.17	4.44	2.73	3.45	2
ADORA1	134	NM_000674	GUGCGGU, CCACA	0.46	4.93	4.95	3.45	2
BAL	83666	NM_031458	GAUUAA, CAGAAU	0.93	6.13	3.26	3.44	2
TIEG2	8462	NM_003597	UUGUAU, GCGCG	0.45	5.48	4.39	3.44	2
PLAGL2	5326	NM_002657	UCAAAU, GGAAGU	2.55	2.98	4.77	3.43	1
SUV420H2	84787	NM_032701	UUACGU, CACGAC	3.04	3.37	3.87	3.43	3

LOC388567	388567	NM_001023561	GGCCAA, AUGUA	2.27	4.67	3.34	3.43	2
C7ORF33	202865	NM_145304	GACGAA, UGUCAU	5.62	2.31	2.34	3.42	1
TBP	6908	NM_003194	AAACUU, GCGGU	0.18	4.72	5.37	3.42	2
C19ORF2	8725	NM_134447	GUUGAU, ACACC	-0.09	2.81	7.55	3.42	1
LOC392395	392395	NM_001013664	GGAGGA, GGAAG	0.52	3.62	6.12	3.42	2
NTRK2	4915	NM_001018064	UCUAUA, GAAUG	0.72	5.6	3.91	3.41	2
FLJ32734	146849	NM_144681	CGGACA, GGGAG	2.26	5.02	2.94	3.41	1
GPR173	54328	NM_018969	AGAUUG, GAACC	0.5	5.35	4.37	3.41	2
THOC2	57187	NM_020449	GAGACA, CCUUGA	2.85	2.81	4.55	3.40	1
UROS	7390	NM_000375	GGAUUA, GGAAU	0.31	3.43	6.47	3.40	2
MAP3K15	389840	NM_001001671	GGAUAU, UCAGA	-0.07	5.36	4.89	3.39	2
CGI-48	51096	NM_016001	GGAAAA, CAUGAU	-0.79	5.96	5.01	3.39	2
ACR	49	NM_001097	GUGAUG, GAAAU	3.42	2.72	4.02	3.39	2
RSU1	6251	NM_152724	GAGACA, GCAGA	1.06	3.45	5.65	3.39	2
MYL9	10398	NM_181526	UUGAUA, CAUCCA	-0.62	5.88	4.9	3.39	2
RPL27	6155	NM_000988	UCGUG, GUAUAA	-1.18	7.15	4.19	3.39	2
EPHA2	1969	NM_004431	GAGAUC, CAAGU	4.99	3.4	1.75	3.38	2
SUPT6H	6830	NM_003170	GACAAA, GAACA	1.97	4.59	3.58	3.38	2
MGC46496	285555	NM_174952	AGAUA, GCUAUG	-0.24	4.49	5.89	3.38	2
HSU79266	29901	NM_013299	CGGACA, CCGCU	3.48	0.73	5.92	3.38	2
MEOX1	4222	NM_013999	CGGAGA, GAGCA	4.82	2.99	2.31	3.37	1
MGC21518	147184	NM_145274	GGGUAU, CGGUG	-1.66	5.48	6.28	3.37	2
LOC151835	151835	NM_153635	CGGCUU, UGAAG	2.03	2.68	5.39	3.37	1
WDR40B	139170	NM_178470	UCACAA, GAAUU	-1.17	3.44	7.83	3.37	2
HRB	3267	NM_004504	CACAAA, GAAGCA	0.64	5.39	4.06	3.36	2
SLC22A4	6583	NM_003059	ACGCAA, CUGUU	2.88	4.54	2.66	3.36	1
HLA-E	3133	NM_005516	UCCUUU, CAGGG	0.81	2.58	6.69	3.36	1
TRPC4	7223	NM_016179	GUCAAA, AGAGA	0.55	5.38	4.15	3.36	2
WDR11	55717	NM_018117	CAAGUU, GGAUG	-0.16	6.49	3.75	3.36	2
GFI1	2672	NM_005263	GUUUA, GCUCGG	0.02	5.06	4.99	3.36	2
FLJ32894	144360	NM_144667	GAGCGA, CCAUCU	3.79	1.7	4.56	3.35	2
ELK1	2002	NM_005229	UGUGGA, GCCAGA	2.98	2.01	5.06	3.35	1
APOL5	80831	NM_030642	CGACUG, GAGGAU	2.08	5.55	2.42	3.35	1
HS3ST6	64711	NM_001009606	GUGAAG, UGAAA	3.32	4.15	2.57	3.35	2

SELO	83642	NM_031454	CGACUA, CCGAGG	1.63	5.23	3.18	3.35	2
MAST4	375449	NM_198828	AUAUUA, GAUGA	3.61	3.67	2.72	3.33	2
KIAA0152	9761	NM_014730	GAUCUU, CUAUA	1.21	4.74	4.05	3.33	2
IFNA13	3447	NM_006900	UCUUUA, AGACAA	1.36	5.7	2.93	3.33	1
ZBTB7	51341	NM_015898	CCAGAA, GGACCU	0.85	2.69	6.45	3.33	1
MRPS2	51116	NM_016034	GAGCGA, GGUUG	-0.12	3.7	6.41	3.33	2
KRTAP4-12	83755	NM_031854	GGAAUU, CCUCUA	4.11	3.45	2.42	3.33	2
FLJ20628	55006	NM_017910	UGAUU, CCUCUG	0.4	4.13	5.44	3.32	2
RPL24	6152	NM_000986	ACCUAA, UAGAAA	2.52	3.89	3.55	3.32	2
FLJ32310	123624	NM_152336	AACGAAA, ACUUG	0.39	4.74	4.83	3.32	2
GCLM	2730	NM_002061	UGUGAA, UAUCA	2.94	3.92	3.09	3.32	2
LOXL1	4016	NM_005576	UACGUU, UCGGCA	3.08	2.32	4.54	3.31	2
DR1	1810	NM_001938	UUGGAA, AAGAA	1.85	3.69	4.4	3.31	2
M17S2	4077	NM_005899	CUUUAA, GAACGU	0.32	6.4	3.22	3.31	2
HOXB13	10481	NM_006361	CCUUAA, GAUGC	3.59	2.22	4.12	3.31	2
APCDD1	147495	NM_153000	UAGAAG, GAACUU	2.81	1.92	5.2	3.31	1
ITM2A	9452	NM_004867	UGCUGU, GUCAA	0.7	4.37	4.86	3.31	2
OR4L1	122742	NM_001004717	GCAAUA, ACGAU	4.75	2.55	2.62	3.31	1
FLJ23514	60494	NM_021827	GGAAUU, AAGCAA	3.45	3.28	3.19	3.31	3
LOC440905	440905	NM_001013711	AAGGGA, AAACC	0.67	2.76	6.48	3.30	1
BZRAP1	9256	NM_004758	UACUAU, CAACCU	0.01	4.2	5.69	3.30	2
HIST1H4K	8362	NM_003541	UUCUAU, UCUCC	5.85	2.77	1.28	3.30	1
NR3C2	4306	NM_000901	CCAACA, GUAUCA	2.01	4.73	3.15	3.30	2
MS4A6A	64231	NM_022349	GAGCUU, GGAAA	1.55	3.67	4.67	3.30	2
PITPNM2	57605	NM_020845	CGGCAU, GAAGA	1.5	6.22	2.17	3.30	1
ACE2	59272	NM_021804	UUGAAA, GCCAUU	0.83	4.08	4.98	3.30	2
IL22RA1	58985	NM_021258	CUAUGC, CACCUG	-0.65	3.67	6.86	3.29	2
AD158	84230	NM_032270	UCCGAAA, GAUAA	3.37	3.67	2.84	3.29	2
LOC168850	168850	NM_176814	GCCGCA, UUAUGA	3.82	4.09	1.96	3.29	2
C14ORF147	171546	NM_138288	CGAAUU, AAUAUA	-1.23	5.96	5.14	3.29	2
C14ORF94	54930	NM_017815	CGGCUA, CAACA	2.81	2.91	4.14	3.29	1
SLC9A1	6548	NM_003047	GAUUUA, GGACAA	0.67	6.11	3.08	3.29	2
CCK	885	NM_000729	UGAGUA, GAAUGU	0.37	6.75	2.74	3.29	1
GPX6	257202	NM_182701	GGCCUC, GUGUAU	0.53	4.5	4.82	3.28	2

PDLIM4	8572	NM_003687	CAGGAU, GCACAG	2.69	3.99	3.17	3.28	2
OR8S1	341568	NM_001005203	GACCAU, CUUCAC	2.64	3.68	3.53	3.28	2
MPP2	4355	NM_005374	JGUAAC, GCUACG	1.18	5.17	3.5	3.28	2
FAM8A1	51439	NM_016255	ACGUAA, CAACCA	0.25	6.27	3.33	3.28	2
GPR109B	8843	NM_006018	GGCCUA, CAGGUG	5.12	1.96	2.76	3.28	1
ACOXL	55289	NM_018308	CGCCUA, AAAUCG	3.88	2.82	3.14	3.28	2
MANSC1	54682	NM_018050	UCGGAA, GUGGGA	3.6	3.37	2.85	3.27	2
CRYAA	1409	NM_000394	GAGUUC, GAAGGU	2.19	3.07	4.56	3.27	2
G6PC	2538	NM_000151	CACAUU, GAAUCU	1.28	3.33	5.21	3.27	2
EFHD1	80303	NM_025202	GAGUAA, CUUCAC	0.19	5.77	3.86	3.27	2
OSGEP	55644	NM_017807	AUCCUA, AUACGC	-0.18	5.39	4.61	3.27	2
RANBP17	64901	NM_022897	GAUCGU, GAGCAC	3.03	1.86	4.91	3.27	2
PPIA	5478	NM_021130	GCAUUU, CAAUGU	0.5	4.72	4.58	3.27	2
ELAC1	55520	NM_018696	GCGCAU, AGAAAU	0.44	3.96	5.39	3.26	2
MOBP	4336	NM_182935	AUCGGAA, ACACUA	6.53	1.11	2.14	3.26	1
FLJ20294	55626	NM_017749	GAGAAA, CAGUAA	0.34	2.29	7.15	3.26	1
KIAA1683	80726	NM_025249	GUAAGU, GGACAU	3.66	2.31	3.8	3.26	2
HIP1R	9026	NM_003959	CGAUCA, UGAAUC	1.48	4.36	3.93	3.26	2
ADPRHL2	54936	NM_017825	GGAGUA, CCAAAG	5.07	1.9	2.79	3.25	1
C14ORF149	112849	NM_144581	CAGAAG, UAAAGC	1.43	5	3.32	3.25	2
PAP	5068	NM_138937	CCUAUG, CACCGA	1.63	3.15	4.95	3.24	2
NDN	4692	NM_002487	AUGAAU, AAAGUA	0.9	3.04	5.79	3.24	2
MATN3	4148	NM_002381	GUAUUG, AAGGUC	1.22	3.86	4.64	3.24	2
TTN	7273	NM_133379	JAAUUA, GAAUUG	4.35	3.22	2.14	3.24	2
CLEC14A	161198	NM_175060	GGCGA, CAAUCAC	0.66	5.69	3.35	3.23	2
KRTAP18-11	386678	NM_198692	CAAUAA, GCAGGU	4.5	3.76	1.44	3.23	2
MAPK4	5596	NM_002747	AGUUCA, UAACAA	2.17	3.94	3.58	3.23	2
OR5D16	390144	NM_001005496	GAAUUA, AAACA	2.17	4.38	3.14	3.23	2
RELA	5970	NM_021975	AGGAAA, GGCUA	2.05	4	3.64	3.23	2
MYCBP	26292	NM_012333	UAUGAA, GCCUAC	0.23	6.96	2.49	3.23	1
GK001	57003	NM_020198	GCGACU, GAGCAG	2.45	4.52	2.7	3.22	1
DUFD1	113115	NM_138419	GAAGA, AGGAGU	0.74	5.11	3.82	3.22	2
HPS4	89781	NM_152843	UGUCA, CAAUGC	0.27	3.05	6.35	3.22	2
COMMD2	51122	NM_016094	UCGCAA, GUGUGC	2.18	2.49	4.99	3.22	1

UBXD2	23190	NM_014607	CGGAAA, CAAGAA	1.19	2.19	6.28	3.22	1
BCL2A1	597	NM_004049	GCAAUA, GGGAAG	4.95	2.22	2.48	3.22	1
LAMA4	3910	NM_002290	JUAUAU, CCAGUG	2.49	3.67	3.48	3.21	2
FLJ35681	283897	NM_175900	GGACUA, CAGCAC	1.23	5.18	3.23	3.21	2
CFDP1	10428	NM_006324	GAGCGA, GCCGUC	2.76	4.48	2.39	3.21	1
KIAA1363	57552	NM_020792	GGAAUA, GCAAU	1.86	2.92	4.85	3.21	1
SH3MD1	9644	NM_014631	CCUUUA, GGACGU	1.47	4	4.15	3.21	2
PAQR6	79957	NM_198406	CGCCUA, GGGUAU	2.8	4.04	2.77	3.20	1
OR4D10	390197	NM_001004705	UCGAUA, AGACUU	1	4.33	4.28	3.20	2
PDGFA	5154	NM_033023	CCCAUGU, CCACUA	1.6	4.89	3.11	3.20	2
RHOJ	57381	NM_020663	GAUUGA, UCAGA	1.12	2.53	5.95	3.20	1
POLR2E	5434	NM_002695	GAGCUA, CCUGUG	1.15	4.52	3.92	3.20	2
LOC339896	339896	NM_207359	GAGGAA, GGAAGA	0.58	4.66	4.35	3.20	2
GRM5	2915	NM_000842	CGGAAA, GAGAUA	2.51	3.91	3.16	3.19	2
TTC6	115669	NM_001007795	GACUA, UGGUAA	1.69	3.99	3.9	3.19	2
BRPF3	27154	NM_015695	GCGCCA, UGAUUG	3.9	3.92	1.75	3.19	2
TBC1D15	64786	NM_022771	GAACUA, CCAAUG	2.05	2.85	4.67	3.19	1
ARCH	339487	NM_178547	GUGUCU, CAGCAG	1.94	4.37	3.26	3.19	2
BCKDHA	593	NM_000709	UCACUA, UCGGCG	1.54	3.92	4.11	3.19	2
GLO1	2739	NM_006708	JUGAAA, CUUCUU	1.51	3.51	4.55	3.19	2
OR2A14	135941	NM_001001659	CUGAUU, CAUUCU	1.11	4.86	3.6	3.19	2
UNG2	10309	NM_021147	GCGACU, CAUAAA	0.67	4.15	4.74	3.19	2
DAZ3	57054	NM_020364	GGAUAU, GAAGUU	-0.83	5.29	5.1	3.19	2
KLF16	83855	NM_031918	JACAAG, GGCCUC	3.9	2.15	3.5	3.18	2
DGKI	9162	NM_004717	AUAUAU, AAGAUG	3.68	3.68	2.19	3.18	2
ZIC2	7546	NM_007129	GGUGGA, CAACUG	0.9	4.24	4.41	3.18	2
RPL26	6154	NM_000987	ACGGAA, CCGAAG	0.14	5.93	3.48	3.18	2
KLK14	43847	NM_022046	CCAUACG, CUGCAA	3.72	1.49	4.33	3.18	2
LOC55924	55924	NM_198926	GAAAGC, UCGACA	5	2.45	2.08	3.18	1
FLJ25818	146779	NM_173503	GACAUA, GUCCCU	4.03	3.03	2.47	3.18	2
GDAP1L1	78997	NM_024034	CCGUCA, UAGUUU	1.12	3.41	5	3.18	2
JMJD1B	51780	NM_016604	GUGAAU, CAGAAU	2.9	2.55	4.08	3.18	1
KHSRP	8570	NM_003685	CUACGGA, GGACA	2.27	3.99	3.27	3.18	2
EEF1E1	9521	NM_004280	CUACUA, CUUCAU	-1.21	4.42	6.31	3.17	2

OVGP1	5016	NM_002557	CGUGA, UCAUC	2.2	3.14	4.17	3.17	2
HSN2	378465	NM_213655	CGGACA, UUUCA	4.18	3.48	1.84	3.17	2
NTNG2	84628	NM_032536	GAGUAA, ACGUC	3.07	2.09	4.34	3.17	2
POLR3C	10623	NM_006468	AACGU, GCAGCG	0.71	4.43	4.36	3.17	2
P2RY6	5031	NM_176797	AAGGU, GGAAU	0.61	5.27	3.62	3.17	2
HCBP6	65991	NM_023934	GUAUU, UAGAA	2.91	3.81	2.77	3.16	1
COG7	91949	NM_153603	CGGCUU, UUACU	2.35	1.85	5.27	3.16	1
MGC26989	254268	NM_152763	UGCAUA, AGACU	2.17	2.29	5.01	3.16	1
MOCOS	55034	NM_017947	CUAUA, GCUGGA	0.24	5.98	3.23	3.15	2
C4ORF6	10141	NM_005750	GCCUGA, GAGUU	2.67	2.95	3.83	3.15	1
FLJ25286	153443	NM_152546	GGAGAA, GAAGC	-0.38	6.2	3.62	3.15	2
SLC15A2	6565	NM_021082	GGGAAU, CCACAA	-1.92	4.75	6.61	3.15	2
BRD8	10902	NM_183359	GCAUAC, AAUAGU	6.52	2.16	0.76	3.15	1
LTBP2	4053	NM_000428	JGCCAA, CCACAU	4.96	2.25	2.23	3.15	1
HSFY2	159119	NM_001001877	CCUUUA, UCAGA	2.56	3.63	3.25	3.15	2
ZNF442	79973	NM_030824	AAGACA, GAAAG	5.65	1.52	2.26	3.14	1
EIF2C4	192670	NM_017629	ACGAAA, CAAUAU	1.92	4.25	3.26	3.14	2
NCR2	9436	NM_004828	GGUAUC, GGACA	0.13	3.87	5.43	3.14	2
VLDLR	7436	NM_001018056	UAUUA, CUGAAA	2.33	2.48	4.61	3.14	1
CART	9607	NM_004291	CAAGAA, GCAAGG	0.18	5	4.23	3.14	2
CYP4F12	66002	NM_023944	JAACGA, UUGCAG	0.09	4.77	4.55	3.14	2
C14ORF58	55640	NM_017791	GACUCA, GCACA	1.16	3.62	4.62	3.13	2
DXS1283E	8228	NM_004650	AUGUUA, GAUCG	4.26	2.59	2.55	3.13	1
KRT16	3868	NM_005557	AGAACU, GACCAA	-0.69	3.39	6.69	3.13	2
KCNA2	3737	NM_004974	AUUGAA, GGAGA	0.77	4.59	4.04	3.13	2
SFI1	9814	NM_014775	GAAUUG, GGAAG	0.75	4.02	4.61	3.13	2
FAM40B	57464	NM_001134336	GGGAAU, AACCU	2.34	2.34	4.69	3.12	1
C6ORF128	221468	NM_145316	UACAAC, GAUCA	1.57	3.74	4.06	3.12	2
PPP1R12B	4660	NM_032103	AUCCGA, GAUAU	1.47	2.83	5.06	3.12	1
TKTL1	8277	NM_012253	GAAUGG, UAGCA	-1.14	4.6	5.89	3.12	2
ABTB2	25841	NM_145804	AUGUA, CCACAC	6.08	1.08	2.18	3.11	1
RIS1	25907	NM_015444	AUAUA, AGGGC	1.75	3.87	3.72	3.11	2
UNQ8193	139322	NM_198450	GUUUCA, CUAGG	0.85	5.56	2.93	3.11	1
UFC1	51506	NM_016406	GAUCA, AAUAUG	0.09	4.22	5.03	3.11	2

KSP37	83888	NM_031950	AAAGAA, CAGACU	3.57	2.35	3.4	3.11	2
GLI3	2737	NM_000168	GCCUUU, GAACAG	4.9	1.56	2.85	3.10	1
TEAD2	8463	NM_003598	CAGUAU, GCAGUU	4.23	2.61	2.47	3.10	1
POLG2	11232	NM_007215	CACGAA, GGCGUA	1.07	4.92	3.32	3.10	2
MART2	55733	NM_018194	GCGUUA, GAAUU	1.5	3.8	4	3.10	2
FSTL5	56884	NM_001128428	ACGGUA, CAUGUA	0.95	4.64	3.71	3.10	2
NDST1	3340	NM_001543	CACAGA, GCGCAC	3.8	2.4	3.08	3.09	2
ITGB4BP	3692	NM_181467	GGAUGA, GAUCGC	1.37	5.18	2.73	3.09	1
OR8A1	390275	NM_001005194	GAGCUU, UCUCAA	0.47	4.68	4.13	3.09	2
STRC	161497	NM_153700	GAUGUA, ACAGAC	3.78	3.05	2.44	3.09	2
PMM2	5373	NM_000303	UCCUGA, AAAUU	3.42	2.48	3.36	3.09	2
OR1M1	125963	NM_001004456	UCAUA, GGAGCA	1.04	4.52	3.7	3.09	2
KIF21B	23046	NM_017596	GUUAA, CGGGUA	3.25	2.85	3.15	3.08	2
C14ORF45	80127	NM_025057	CAGGUA, GCAAGA	3.13	2.65	3.47	3.08	2
RIOK2	55781	NM_018343	GGCAAA, GUCCAG	0.05	4.71	4.49	3.08	2
ATP1B1	481	NM_001001787	GGCGUA, AUGAAC	-0.61	6.08	3.78	3.08	2
ZFAND6	54469	NM_019006	GUACAA, CAGAUU	-0.73	2.3	7.68	3.08	1
CHFR	55743	NM_018223	CUCUAU, GCACUC	5.14	0.46	3.64	3.08	2
GUCY2D	3000	NM_000180	GGAGGA, UCACGG	1.02	4.2	4.02	3.08	2
ING1L	3622	NM_001564	GGUAGU, GAACAG	0.64	4.81	3.78	3.08	2
CDKN3	1033	NM_005192	AAUUA, UGGGAG	0.47	3.82	4.92	3.07	2
KIAA0913	23053	NM_015037	AGACA, GGGAUC	4.19	2.44	2.59	3.07	1
MGC45873	148398	NM_152486	UCGGAA, AAGAGC	3.11	3.7	2.39	3.07	2
FLJ23420	80131	NM_025061	GGCAUU, GGAGAA	5.34	1.68	2.18	3.07	1
PIR51	10635	NM_006479	UAGAUU, GAAUC	1.91	4.17	3.12	3.07	2
GMRP-1	84280	NM_032320	UUACUA, UAGUAC	0.91	3.52	4.77	3.07	2
ZNF284	342909	NM_001037813	GAGAUA, CCGUAG	0.36	4.82	4.01	3.06	2
MGC35521	246330	NM_145065	CGUCAU, CUGAGA	-0.85	4.02	6.02	3.06	2
TGM6	343641	NM_198994	CAGCAA, UGGCAC	3.68	3.62	1.88	3.06	2
PLCL4	9651	NM_014638	UGGAAA, CCUUUC	2.98	3.94	2.26	3.06	1
TAS2R10	50839	NM_023921	UCAAGA, UUGCAU	1.27	5.11	2.79	3.06	1
SOX14	8403	NM_004189	CUGUAA, AGACUC	5.35	2.21	1.59	3.05	1
LENG8	114823	NM_052925	CAUUAA, GGACAA	3.8	2.1	3.25	3.05	2
C10ORF99	387695	NM_207373	GGUGUA, GCAUCC	1.39	3.43	4.33	3.05	2

NEUROD1	4760	NM_002500	CUAAGA, UCCCAU	0.34	3.94	4.87	3.05	2
CDC25C	995	NM_022809	GUUUAA, GAGAG	4.89	2.26	1.99	3.05	1
NEUROG2	63973	NM_024019	GAAGA, GGGAUU	3.16	2.29	3.68	3.04	2
MLSTD1	55711	NM_018099	JAAUAU, UGCUAU	-0.11	3.92	5.32	3.04	2
GDF7	151449	NM_182828	GACCCA, CCGAAA	2.6	3.58	2.95	3.04	1
BZW2	28969	NM_014038	UGGCGA, CUAACA	0.79	4.33	4	3.04	2
TIMP3	7078	NM_000362	GGUAUC, CCGACA	2.29	2.64	4.18	3.04	1
ZNF83	55769	NM_018300	JCAAAU, CAACGU	2.07	3.2	3.84	3.04	2
SLC6A5	9152	NM_004211	GAGGGA, AGAUCA	0	4.6	4.51	3.04	2
MHC2TA	4261	NM_000246	CAAUAA, GAAGU	2.41	4.29	2.4	3.03	1
RAB39	54734	NM_017516	CGGAUU, UCACA	3.28	4.27	1.54	3.03	2
TTC10	8100	NM_006531	GGUGAU, AGGCAA	2.15	3.11	3.83	3.03	2
KIAA1838	84498	NM_032448	GUGUAU, GAUGA	0.73	4.11	4.25	3.03	2
CLOCK	9575	NM_004898	AACCUA, GAACAA	2.37	2.69	4.02	3.03	1
CD276	80381	NM_025240	JGCGAAU, CUCCA	2.25	2.9	3.92	3.02	1
ZFHX2	85446	NM_033400	GCAUGA, GUUGG	-0.21	3	6.28	3.02	2
PSMC4	5704	NM_153001	JGCGCAU, UCUAC	4.23	1.74	3.09	3.02	2
PTPN3	5774	NM_002829	CAAGUU, GUUGA	1.82	4.11	3.13	3.02	2
AGPS	8540	NM_003659	AUGUCA, GGACCU	0	3.91	5.14	3.02	2
PTPN2	5771	NM_080423	AUGAUC, AUACA	1.58	2.39	5.07	3.01	1
ZFP1	162239	NM_153688	AGAUUA, CUGGAA	2.1	2.9	4.04	3.01	1
HIST1H3B	8358	NM_003537	CGGAAA, GCUAA	0.53	4.51	4	3.01	2
SST	6750	NM_001048	GAGAUC, GAACC	2.76	2.09	4.18	3.01	1
KRTAP5-9	3846	NM_005553	ACCCUU, GCUCUA	1.36	2.96	4.69	3.00	1
PRDM10	56980	NM_199439	GAUUUA, CCAAGA	0.82	4.08	4.11	3.00	2
ELF3	1999	NM_004433	GUCUUU, GCCAU	2.5	3.46	3.05	3.00	2
PDIK1L	149420	NM_152835	UGAGUA, UGAAA	2.48	3.15	3.38	3.00	2
Raptor	57521	NM_020761	CGACAA, AGAAGG	4.56	3.36	1.08	3.00	2
CRAT	1384	NM_144782	GACCAA, GUACCA	4.18	2.01	2.81	3.00	1
FBXL7	23194	NM_012304	AAGUGA, GUCUCA	2.41	2.24	4.35	3.00	1
PRRG3	79057	NM_024082	GCAUGA, CAGUCU	0.27	4.47	4.26	3.00	2
ZNF335	63925	NM_022095	GCAAGU, GCACCA	2.71	4.62	1.66	3.00	1
C18ORF19	125228	NM_152352	GACUUA, GUACGU	2.14	1.7	5.15	3.00	1
KCTD9	54793	NM_017634	UCGUUU, UCAAG	1	4.56	3.43	3.00	2

RPS21	6227	NM_001024	GAGUCA, CAGGUU	-1.56	3.76	6.79	3.00	2
MLL4	9757	NM_014727	CAACUA, GGGCGA	4.4	3.1	1.48	2.99	2
FHOD1	29109	NM_013241	ACACCGA, ACGCAU	3.3	2.06	3.62	2.99	2
HSP90B1	7184	NM_003299	GCUCUA, GGACGG	2.43	2.37	4.18	2.99	1
FOSL2	2355	NM_005253	UAGAUA, CCACUC	1.79	4.55	2.64	2.99	1
LOC146167	146167	NM_001080442	GCCUAU, GCUUCG	-0.71	5	4.69	2.99	2
MGC5528	79075	NM_024094	GUUAA, UAGAUG	1.64	4.56	2.78	2.99	1
SURB7	9412	NM_004264	AGCUAA, ACAAGA	3.12	2.6	3.25	2.99	2
C10ORF61	26123	NM_001013840	AACUUA, UGUAAU	0.26	3.72	4.99	2.99	2
RCL1	10171	NM_005772	GGCUU, CUACAG	2.67	2.76	3.54	2.99	1
LOC55954	55954	NM_001003692	GCGAGA, CUGACA	2.91	4.43	1.62	2.99	1
NUDT3	11165	NM_006703	GGACGU, UAAAAU	0.55	2.56	5.85	2.99	1
HDAC4	9759	NM_006037	GGCUAA, GAGUGU	3.13	4.84	0.99	2.99	2
BAIAP2	10458	NM_006340	GUCACA, GUAUGC	0.73	3.83	4.39	2.98	2
ZBTB42	100128927	NM_001137601	ACGUGU, CCAGCC	0.58	5.31	3.06	2.98	2
MGC11256	79174	NM_024324	GAAUGC, GCUGCC	1.01	3.39	4.54	2.98	2
FLJ40126	283461	NM_001031748	GGUCUA, GUAAAC	0.61	3.85	4.48	2.98	2
TAF1B	9014	NM_005680	GACUAA, UGUGAA	1.98	3.14	3.82	2.98	2
CENTA1	11033	NM_006869	GCGACA, UCGGGA	-1.03	5	4.97	2.98	2
WFDC9	259240	NM_147198	UAGACA, GGGUAC	5.45	1.44	2.04	2.98	1
LOC653314	653314	NM_001080544	CGGAUU, UGGCCA	0.48	5.51	2.94	2.98	1
CTSD	1509	NM_001909	CGCUCA, GGCACA	-0.33	5.17	4.09	2.98	2
TCF1	6927	NM_000545	GCAGCG, GAGAGA	2.26	3.89	2.77	2.97	1
GABRA6	2559	NM_000811	UACGUU, GAUAU	4.73	1.95	2.23	2.97	1
FLJ31438	130162	NM_001135598	AUUAAA, AGAGA	2.43	2.9	3.58	2.97	1
SIRT1	23411	NM_012238	GAGAUA, GGAUAC	1.32	2.64	4.95	2.97	1
HIMAP4	55303	NM_018326	GUCGUA, GCAAAC	0.93	4.75	3.23	2.97	2
MS4A2	2206	NM_000139	CAUCCU, GGGAAC	0.37	3.73	4.81	2.97	2
PTX1	51290	NM_016570	GCGGAU, GAGGC	2.17	3.16	3.57	2.97	2
MOSPD1	56180	NM_019556	ACGGAU, CUACAA	0.94	3.14	4.82	2.97	2
DEFB130	245940	NM_001037804	AGCCCUA, CAAACU	-0.12	5.49	3.53	2.97	2
DEC1	50514	NM_017418	GUGUUA, AGAGAG	0.34	5.18	3.38	2.97	2
HIST1H3E	8353	NM_003532	GCAAAC, GAGAGG	2.44	3.64	2.8	2.96	1
PIWIL4	143689	NM_152431	AUAAUC, CGACAG	5.21	1.63	2.03	2.96	1

EAF2	55840	NM_018456	AGUUA, GGAGAA	2.02	3.17	3.68	2.96	2
ZNF581	51545	NM_016535	CGAGUA, GGUAA	2.06	1.83	4.97	2.95	1
CTNNA3	29119	NM_013266	GAGAUU, CGGAAC	1.11	4.22	3.53	2.95	2
C9ORF25	203259	NM_147202	UGGCUU, ACAUGA	-0.42	3.27	6.01	2.95	2
C6ORF4	10758	NM_147200	GCGGUA, CCAGAA	1.17	3.1	4.58	2.95	2
C16ORF33	79622	NM_024571	GGCGAG, CCGGAA	1.14	2.91	4.8	2.95	1
ORC3L	23595	NM_181837	GAAUUA, CCACAA	0.19	3.37	5.29	2.95	2
AXL	558	NM_001699	GUAUCA, GACGAA	-0.42	5.49	3.78	2.95	2
UNC84A	23353	NM_025154	UACUUU, GCGCU	3.74	2.98	2.12	2.95	1
CACNA2D1	781	NM_000722	AUUAUA, GAACAA	3.22	2.63	2.99	2.95	1
EMID2	136227	NM_133457	CGGAGA, GUGACA	0.73	3	5.11	2.95	2
SLC32A1	140679	NM_080552	UCCGCA, GGGAGG	2.34	2.4	4.09	2.94	1
NMNAT3	349565	NM_178177	CUCGAC, GGCCAA	1.21	4.16	3.46	2.94	2
DKFZP547I048	127254	NM_001002912	AAGAAA, GAGCGA	0.19	4.81	3.83	2.94	2
UACA	55075	NM_018003	GGACUA, GCAAGA	0.05	4.35	4.43	2.94	2
GSTK1	373156	NM_015917	ACAUG, GAGAAG	-0.57	2.58	6.81	2.94	1
PSMC3	5702	NM_002804	AAUAC, GGACAAU	0.34	5.03	3.44	2.94	2
GPR68	8111	NM_003485	CGUGUA, GACCAU	0.91	4.1	3.8	2.94	2
ARHGAP24	83478	NM_001025616	GCGAAA, GUGUUA	2.19	2.71	3.9	2.93	1
FLJ12787	84135	NM_032175	GCGUUU, AAUAAA	-0.19	5.68	3.31	2.93	2
DAF	1604	NM_000574	CAGAUA, UCGGUA	4.59	2.1	2.1	2.93	1
GREM2	64388	NM_022469	GAUAUA, GGUCUU	1.64	3.71	3.44	2.93	2
FGFR1OP	11116	NM_194429	CGUUUA, GGAAGC	1.47	4.96	2.36	2.93	1
RP13-36C9.6	441521	NM_001007551	GAAUCA, CCGAUC	5.69	0.42	2.67	2.93	1
RPS25	6230	NM_001028	UAUGAU, CAAAGA	2.95	2.99	2.84	2.93	0
ARRB2	409	NM_199004	GAGAUU, GGGCUU	1.41	3.87	3.5	2.93	2
TNPO1	3842	NM_153188	GCCUUA, GUAAAU	0.89	3.29	4.6	2.93	2
HSD17B3	3293	NM_000197	GGACUA, CAUCUA	0.6	3.51	4.67	2.93	2
NINJ2	4815	NM_016533	AGUUA, GAAAAG	2.91	2.62	3.24	2.92	1
LRCH1	23143	NM_015116	CGGACA, UCAGUU	2.5	2.82	3.45	2.92	1
FLJ20481	54947	NM_017839	GCGUUA, CAUUUA	-0.27	5.87	3.17	2.92	2
ADAM28	10863	NM_021777	UAGAGU, UGAAGU	1.3	4.2	3.25	2.92	2
TAFA3	284467	NM_182759	CGGCUCA, CCACCA	1.02	3.05	4.67	2.91	2
IGSF2	9398	NM_004258	GACAAA, AAAGUC	0.97	3.57	4.2	2.91	2

HLA-DRB4	3126	NM_021983	GAGCUU, GCGCU	0.71	4.03	4	2.91	2
TEX10	54881	NM_017746	GAACGA, CCAAU	-0.75	6.05	3.44	2.91	2
TBX4	9496	NM_018488	CAGGUU, GCUAU	2.5	4.11	2.12	2.91	1
CALB2	794	NM_007088	GAGCAU, CCUGA	1.95	2.99	3.79	2.91	1
SLITRK1	114798	NM_052910	CGGAAA, CCGAAG	-0.58	4.25	5.06	2.91	2
ZNF545	284406	NM_133466	GGUUA, CGGCA	1.06	3.72	3.94	2.91	2
CENTD2	116985	NM_015242	UACUCU, CCGCA	-0.3	3.68	5.34	2.91	2
C14ORF133	63894	NM_022067	GCUAUC, GAACA	-0.83	5.84	3.71	2.91	2
KIDINS220	57498	NM_020738	GAGACU, GAUGA	3.43	2.96	2.32	2.90	1
ORC1L	4998	NM_004153	GUCGAA, CAUCGU	1.65	2.72	4.34	2.90	1
VPRBP	9730	NM_014703	UGCGCA, GGAAU	1.06	3.29	4.36	2.90	2
FLJ21963	79611	NM_024560	CGUCAA, UCGAGU	2.09	3.44	3.18	2.90	2
STX18	53407	NM_016930	GAGUUA, GUUCA	1.04	3.68	3.98	2.90	2
OR7C2	26658	NM_012377	AAAGA, GCUGAC	0.9	5.08	2.72	2.90	1
RIPK1	8737	NM_003804	CGCAAA, GCACAA	0.78	3.67	4.25	2.90	2
SP110	3431	NM_004510	GUGAAU, GAGGA	0.75	5.1	2.84	2.90	1
IGFALS	3483	NM_004970	GGGAA, GGAAUA	-1.61	4.95	5.35	2.90	2
HOP	84525	NM_139211	AGGCAU, CGAGGA	4.58	2.45	1.65	2.89	1
FLJ20241	54862	NM_017721	GAGGUC, GCGGA	1	3.25	4.43	2.89	2
FLJ32859	152405	NM_152539	GAAGAU, GUACA	0.73	3.68	4.27	2.89	2
OC90	729330	XM_001129959	GCGGAA, AGAUCA	4.58	1.5	2.59	2.89	1
XRN2	22803	NM_012255	GUCGUA, UCGUU	1.37	3.96	3.34	2.89	2
CD24	934	NM_013230	CAUCUA, GAUCAG	1.06	2.83	4.78	2.89	1
ERP70	9601	NM_004911	UACAAA, GCGAGU	0.01	3.63	5.03	2.89	2
LOC338759	359787	NM_199286	GUGUAA, GGGCU	3.79	2.04	2.83	2.89	1
FLJ20647	55013	NM_017918	UCGGAA, CGACA	2.76	1.72	4.18	2.89	1
GHSR	2693	NM_004122	UGAAA, CUGGAC	-0.05	5.11	3.6	2.89	2
CES1	1066	NM_001266	GAGUU, AUUCU	2.3	2.7	3.65	2.88	1
STXBP3	6814	NM_007269	CGUUA, GUAAA	3.72	1.82	3.09	2.88	2
C20ORF12	55184	NM_018152	GACUAA, GAAUU	1.81	2.69	4.13	2.88	1
SDC4	6385	NM_002999	CAACAA, GAAUC	1.32	3.9	3.41	2.88	2
OR8G1P	26494	NM_001002905	UGUCAA, AUAAA	4.26	3.19	1.17	2.87	2
HUS1	3364	NM_004507	GAUAUU, CCAUAA	2.28	3.16	3.18	2.87	2
FLJ40311	124535	NM_001080439	GGACUU, GACCA	1.96	3.34	3.3	2.87	2

CXCL14	9547	NM_004887	CAGGUA, GCUAC	1.16	4.02	3.42	2.87	2
MTUS1	57509	NM_020749	GCGUUU, UAGGG	-1.02	5.04	4.58	2.87	2
LOC388931	388931	NM_001080473	GACUUA, CUGUG	0.36	4.25	3.98	2.86	2
TRAM1	23471	NM_014294	UUAUUA, GAUAAU	3.68	1.61	3.29	2.86	2
RIF1	55791	NM_001006945	GAGAUG, CUUGAA	2.81	2.63	3.14	2.86	1
ATR	545	NM_001184	CAGAUA, CCACGA	0.05	5.64	2.89	2.86	1
KIAA1361	57551	NM_020791	UCUAA, CUAAAG	-0.61	5.04	4.15	2.86	2
CCL15	6359	NM_032965	UUGCU, GUCCGG	2.78	3.54	2.25	2.86	1
DCNP1	140947	NM_130848	GAUUA, AGGAUA	-0.53	5.03	4.07	2.86	2
ITPK1	3705	NM_014216	GGCACA, AGAGUU	1.33	4.04	3.19	2.85	2
ST8SIA4	7903	NM_175052	ACGAGA, AUUAGG	2.13	0.76	5.67	2.85	1
RARRES1	5918	NM_002888	AAGUUC, UGGAAC	5.63	0.72	2.2	2.85	1
UBL4	8266	NM_014235	GGAUU, AGAAGC	1.85	3.1	3.6	2.85	2
CELSR2	1952	NM_001408	UUCUA, GGUGAC	3.61	2.54	2.39	2.85	1
MGC39325	90362	NM_147189	CGUUAA, GAUAAU	2.13	2.95	3.46	2.85	1
MGC3234	66005	NM_023947	AAGUC, GAGUGU	2.03	3.23	3.28	2.85	2
HYAL4	23553	NM_012269	AGACGA, GGAAUU	1.12	3.39	4.03	2.85	2
MGC42090	256130	NM_152774	GUUUAA, CUAGUA	-0.11	4.85	3.8	2.85	2
SCGB3A2	117156	NM_054023	CCCUUU, CAUUAA	1.39	3.7	3.43	2.84	2
TMIE	259236	NM_147196	GGAAA, GUAAUG	1.16	4.11	3.25	2.84	2
NIFIE14	10430	NM_032635	GUACAA, ACAUCA	-0.29	4.14	4.67	2.84	2
MDS009	56986	NM_020234	ACCAAA, GGACCU	-1.63	3.98	6.17	2.84	2
LEAP-2	116842	NM_052971	UAAACU, CAUAAU	3.27	3.26	1.98	2.84	2
GCKR	2646	NM_001486	UACUGU, GUCCGU	-0.69	4.7	4.5	2.84	2
ATG16L1	55054	NM_198890	GGGACA, GCAUUC	5.64	2.35	0.5	2.83	1
LOC343071	343071	NM_001039361	GGAAAA, GAUGA	2.67	3.2	2.62	2.83	1
NOLA1	54433	NM_032993	GUCUUA, CCGCGG	2.71	3.44	2.33	2.83	1
LOC342357	342357	NM_001012981	ACGUAA, GCUGAA	0.27	4.42	3.79	2.83	2
GNB5	10681	NM_006578	GUGUAC, AUAGGC	-0.36	4.99	3.85	2.83	2
TOE1	114034	NM_025077	GGGAAA, GUGACA	-1.29	5.87	3.9	2.83	2
HMGCS1	3157	NM_002130	UAGUUA, GAACAC	3.9	1.47	3.1	2.82	2
DNAJC1	64215	NM_022365	UCGUAA, GGAAAA	0.34	2.68	5.45	2.82	1
SETDB1	9869	NM_012432	GCACAA, GGGAU	-0.13	5.52	3.08	2.82	2
CYP2J2	1573	NM_000775	GGCAU, UAAAAG	2.8	3.3	2.35	2.82	1

OR4F17	81099	NM_001005240	UGGAAA, CAACG	0.44	3.33	4.68	2.82	2
GRLF1	2909	NM_004491	UGUAA, GAGGG	2.23	3.25	2.96	2.81	1
TRIM52	84851	NM_032765	CAGUA, GGUGG	2.2	2.61	3.63	2.81	1
TM7SF4	81501	NM_030788	GAUAA, CCGGAU	3.03	1.14	4.27	2.81	2
C2ORF30	27248	NM_015701	AUAUA, CGAAGU	2.41	1.66	4.35	2.81	1
GALNT13	114805	NM_052917	AUGAA, GGAAGA	1.89	4.12	2.41	2.81	1
FLJ20527	54967	NM_001031705	UACAU, GGGCCA	0.43	4.62	3.37	2.81	2
NEDD4L	23327	NM_015277	GACUCU, GAUCA	-1.47	6.74	3.14	2.80	2
FLJ11127	54491	NM_019018	GAGAUA, AGGCG	1.47	2.57	4.36	2.80	1
VWF	7450	NM_000552	GCUGUU, AAACG	1.72	2.59	4.09	2.80	1
IL1F5	26525	NM_173170	CUCUAU, GCGCU	1.2	2.67	4.52	2.80	1
LOC389834	389834	NM_001013655	AGCCAA, CACUG	-1.96	5	5.35	2.80	2
CALM2	805	NM_001743	CUUGUA, GAUGG	2.78	1.97	3.63	2.79	1
EPC2	26122	NM_015630	UCGAUA, CAUCA	2.36	3.27	2.75	2.79	1
P2RY8	286530	NM_178129	CAAAUC, GCAAA	3.56	2.77	2.04	2.79	1
GPRC5B	51704	NM_016235	CGUCGU, CAAUG	-1.21	4.16	5.42	2.79	2
PIP5K2A	5305	NM_005028	JAUGGA, GCAUG	2.73	3.5	2.13	2.79	1
PRKACB	5567	NM_207578	GGACUU, GAUAA	2.51	2.77	3.08	2.79	1
BST1	683	NM_004334	GCAUA, CAGACU	2.36	2.1	3.9	2.79	1
TACR2	6865	NM_001057	GUUUGG, CCAAG	1.98	3	3.38	2.79	2
FKBP10	60681	NM_021939	CGACGA, CAACAA	3.07	3.85	1.43	2.78	2
MRO	83876	NM_031939	GAGAUU, AUUCAA	1.86	3.14	3.35	2.78	2
MAPK9	5601	NM_002752	AGGUUA, AGCCA	1.77	2.6	3.98	2.78	1
TTF2	8458	NM_003594	CAAGUA, GCAACA	1.71	3.91	2.73	2.78	1
NPC1	4864	NM_000271	UUAUA, GCGAAC	0.06	4.67	3.62	2.78	2
FBXL16	146330	NM_153350	CGCAUC, GCUCC	2.52	2.62	3.19	2.78	1
C9	735	NM_001737	AUGAGA, GAACC	2.45	3.31	2.57	2.78	1
HIST4H4	121504	NM_175054	GAGAA, CGGCCA	4.24	2.12	1.96	2.77	1
DEFA3	1668	NM_005217	GCAUU, GGAGAA	3.91	0.36	4.05	2.77	2
TIGD4	201798	NM_145720	UCGGAU, AGAUA	1.82	3.48	3.02	2.77	2
RNASEL	6041	NM_021133	ACAAUA, GAACA	1.41	3.07	3.84	2.77	2
GPR149	344758	XM_293580	ACUUU, GCUUUG	1.28	2.88	4.16	2.77	1
GPR115	221393	NM_153838	CCUAAA, CCAAAU	-0.43	4.52	4.23	2.77	2
FLJ10511	55156	NM_018120	UUGAU, GGUAU	4.38	1.84	2.09	2.77	1

FLJ36031	168455	NM_175884	CGGAUG, AAGGUU	0.36	2.27	5.68	2.77	1
C1QL2	165257	NM_182528	UCGAUU, AAUAA	0.98	3.94	3.38	2.77	2
CHAC2	494143	NM_001008708	GCGGGA, CAUUC	0.56	2.58	5.16	2.77	1
ARMC3	219681	NM_173081	CGGGAA, GGAGA	4.32	3.11	0.87	2.77	2
LMBR1L	55716	NM_018113	UCCUUA, GGUCC	2.81	2.59	2.89	2.76	0
THAP9	79725	NM_024672	GCGUUA, UGUCA	1.35	4.22	2.72	2.76	1
OR52D1	390066	NM_001005163	UGGCUA, CCAUGU	2.93	2.67	2.69	2.76	0
CPNE4	131034	NM_130808	GGACUA, CCAAGG	1.08	3.5	3.71	2.76	2
IGSF4D	253559	NM_153184	GGAGAA, GCCAA	1.2	4.97	2.11	2.76	1
LOC90353	90353	NM_145232	ACCUU, UGGCCGU	0.77	4.15	3.37	2.76	2
PRR5	55615	NM_001017529	GGACUA, CCGGC	2.99	1.96	3.32	2.76	1
DDX46	9879	NM_014829	CGGAAA, GGCGA	2.44	2.11	3.72	2.76	1
SLPI	6590	NM_003064	UGCUUA, UGUGA	1.98	3.22	3.07	2.76	2
NEUROD6	63974	NM_022728	GUAAA, AAAUAG	0.07	4.08	4.11	2.75	2
CHIC1	53344	NM_001039840	GUGUA, CAUCAG	1.09	4.33	2.84	2.75	1
AXIN1	8312	NM_181050	CAUUAA, GAGCAA	4.49	1.35	2.41	2.75	1
CA8	767	NM_004056	UUGUU, GGGAAU	3.08	1.92	3.25	2.75	2
LOC400689	57677	NM_020917	AGUUAA, CAACA	1.89	4.27	2.09	2.75	1
LOC96610	96610	NM_080926	ACAUAA, GUUAU	-1.03	4.13	5.15	2.75	2
LOC201725	201725	NM_001008393	GAUUA, GGAGGU	2.49	2.11	3.64	2.75	1
NOL3	8996	NM_003946	CGAGAC, GAAGAU	-1.23	4.77	4.7	2.75	2
GSR	2936	NM_000637	GAUGUU, GGUCU	-1.26	5.17	4.33	2.75	2
KLHL11	55175	NM_018143	UGAUA, AAGGGA	2.39	1.33	4.51	2.74	1
SLC7A2	6542	NM_001008539	CGAAA, GUAAAG	0.2	3.11	4.92	2.74	2
FLJ46247	374786	NM_198529	UGGAAA, GAAUGU	-1.63	6.5	3.36	2.74	2
DFFA	1676	NM_004401	UUGAUA, ACGCAG	1.76	3.74	2.72	2.74	1
RNF122	79845	NM_024787	CUAUUG, UGAAA	0.92	3.43	3.87	2.74	2
IL4I1	259307	NM_152899	GGCAUU, GAGGAU	-0.15	3.2	5.17	2.74	2
CD19	930	NM_001770	CGGCCA, UCAAGA	-0.37	3.72	4.87	2.74	2
KLK7	5650	NM_139277	CAAGGA, CCACAC	2.81	1.65	3.76	2.74	1
ELA1	1990	NM_001971	GGCUAU, UCACCC	1.46	3.05	3.71	2.74	2
SH3KBP1	30011	NM_001024666	GCUGAA, CUAUCC	0.11	4.1	4.01	2.74	2
KIAA1822	84439	NM_032425	GAGGAU, CUACAA	1.79	2.56	3.86	2.74	1
FLJ14075	79954	NM_024894	GGACGA, GUUAAA	2.45	3.98	1.77	2.73	1

TCEB3B	51224	NM_016427	JCGUUA, GAUCAG	3.09	2.54	2.56	2.73	1
ALDH3A2	224	NM_000382	UAAAUU, GCUCA	1.91	3.21	3.06	2.73	2
NANS	54187	NM_018946	GCAAA, GGUGU	1.44	3.7	3.04	2.73	2
KIAA1279	26128	NM_015634	GAUUCA, CAUCAG	2.4	3.16	2.61	2.72	1
MCOLN2	255231	NM_153259	AAGUU, UCAGAU	0.88	4.08	3.21	2.72	2
KIAA1285	27153	NM_015694	GCGAGU, CGUGAU	4.79	2.95	0.42	2.72	1
IL1RAPL1	11141	NM_014271	GAUCAA, AAUCU	4.24	3.36	0.56	2.72	2
TNK1	8711	NM_003985	GUCUAA, GGCGCA	3.33	2.2	2.63	2.72	1
MTHFD1	4522	NM_005956	GAAGUA, GGACG	2.64	2.83	2.69	2.72	0
FLJ38705	286128	NM_173832	AGCACA, CUGAA	2.62	2.1	3.44	2.72	1
APITD1	378708	NM_199294	ACUUU, GAUGU	1.92	2.79	3.45	2.72	1
MGC40047	284521	NM_175911	GAGUA, GCAGGU	1.28	2.74	4.14	2.72	1
NETO2	81831	NM_018092	UGAAA, CCAAUG	4.5	1.6	2.05	2.72	1
OR4K1	79544	NM_001004063	JGGAUA, AGUGCU	3.85	2.27	2.02	2.71	1
ANKH	56172	NM_054027	AGUGAU, GGCCAU	2.72	4.3	1.11	2.71	1
NYD-SP29	126820	NM_145172	CGGAAU, GGAAG	0.89	3.2	4.04	2.71	2
PGR1	93621	NM_033296	GAGAAG, CAUUAA	0.82	2.94	4.37	2.71	1
ZNF354C	30832	NM_014594	GAGAAA, CUUCA	-0.65	2.68	6.1	2.71	1
CXorf64	100130613	NM_001122716	AAUGG, UAGCUU	4.46	0.94	2.72	2.71	1
PTCD2	79810	NM_024754	GCAUAU, GGAAA	1.64	3.26	3.22	2.71	2
EDG8	53637	NM_030760	CAGGUA, AGAAC	1.12	3.97	3.03	2.71	2
ST18	9705	NM_014682	GUGAUA, GCAGA	1.66	3.52	2.93	2.70	1
PKD2	5311	NM_000297	UGUUGU, CAGAU	0.86	4.82	2.42	2.70	1
ZC3HAV1	56829	NM_024625	GGGUAA, GGUAA	1.06	2.49	4.55	2.70	1
DUSP21	63904	NM_022076	AGGUAA, CUACAG	0.18	4.42	3.5	2.70	2
SMPX	23676	NM_014332	CGAUUU, CAAUAU	3.48	1.41	3.2	2.70	2
C9ORF37	85026	NM_032937	CAAAUA, UGACAA	2.59	1.28	4.22	2.70	1
FLJ90650	206338	NM_173800	GCAUU, ACGUGU	1.71	3.25	3.13	2.70	2
SCXA	100129885	NM_001008271	GCCCAA, ACCAGA	0.54	4.35	3.2	2.70	2
STK32B	55351	NM_018401	UCAUAU, CAGAA	-0.86	6.25	2.69	2.69	1
MRAP	56246	NM_206898	AACAU, GUGAUC	2.71	4.07	1.28	2.69	1
LRRC8	56262	NM_019594	CCGGCA, CAAAGC	-1.65	3.43	6.28	2.69	2
MGC33329	256979	NM_152782	CGAGAU, ACGAAA	3.94	2.26	1.86	2.69	1
PRDM6	93166	XM_937753	GAAUAA, GGGCAC	2.6	2.34	3.12	2.69	1

SLC2A14	144195	NM_153449	JGUUAA, CAAGGA	-1.24	3.24	6.06	2.69	2
EFNA1	1942	NM_182685	ACUACA, GAAUG/	2.59	3.41	2.05	2.68	1
RTEL1	51750	NM_016434	:AAUUA, GACAUU	0.94	3.95	3.16	2.68	2
PRSS7	5651	NM_002772	AGCAAA, CGAUGU	-0.38	2.72	5.71	2.68	1
KLHL10	317719	NM_152467	JGCUUA, UCACUC	-0.63	3.06	5.62	2.68	2
DMAP1	55929	NM_001034024	GCGUCU, CUGCGC	-0.67	5.37	3.35	2.68	2
SLC39A2	29986	NM_014579	GGGCUA, GCAGUC	5.15	0.86	2.03	2.68	1
KIAA0528	9847	NM_014802	AAUUUA, GCUCA/	2.18	2.51	3.35	2.68	1
LPPR4	9890	NM_014839	GACUUU, GCAAUU	1.45	2.67	3.92	2.68	1
SFTPD	6441	NM_003019	GCUUG, UGACAG	0.5	4.06	3.48	2.68	2
AASS	10157	NM_005763	GGCUUA, GGUGA(	2.19	2.78	3.06	2.68	1
LOC441518	441518	NM_001078173	UGGUAU, GGUCA	0.44	4.23	3.35	2.67	2
GABARAPL2	11345	NM_007285	CAGAUU, GAUGG/	3.66	1.46	2.89	2.67	1
LCE1C	353133	NM_178351	GGACUA, CGCAA/	1.03	3	3.98	2.67	2
ZNF595	152687	NM_182524	UGAACA, GAGCCU	2.24	2.47	3.29	2.67	1
RAB5C	5878	NM_004583	CAAUUU, GCAAU(	1.87	2.4	3.73	2.67	1
KCNJ10	3766	NM_002241	UCAUUA, GAGCU(	1.54	2.4	4.06	2.67	1
RAD9A	5883	NM_004584	CUCUUA, GCGGA/	-0.08	4.72	3.36	2.67	2
GOT2	2806	NM_002080	CGGUUU, GCUUAU	-0.66	3.3	5.36	2.67	2
EPHA5	2044	NM_182472	GUCGAA, UGAGU(	2.59	1.78	3.62	2.66	1
PJA1	64219	NM_001032396	GACAGU, AAACU(	0.11	2.85	5.03	2.66	1
UGT1A5	54579	NM_019078	GGCGUU, CAUCGA	0.06	3.33	4.6	2.66	2
OR8B8	26493	NM_012378	CAACCA, UGAUAA	0.43	3.36	4.19	2.66	2
MGC33424	143888	NM_153705	GCGCCA, CGAGAC	2.38	2.05	3.54	2.66	1
FLJ13841	79755	NM_024702	CGGAGA, GCCGAU	2.65	2.13	3.19	2.66	1
LOC653121	653121	NM_001040441	AGACGU, AUUCA/	-0.43	3.91	4.48	2.65	2
MNDA	4332	NM_002432	UUAUGU, GUAAG	3.49	1.64	2.83	2.65	1
OR5AC2	81050	NM_054106	GACUAA, GGACUU	-0.18	3.73	4.41	2.65	2
KIAA1040	23041	NM_015026	ACACUA, GGCAGU	1.34	3.76	2.86	2.65	1
RPIP8	10900	NM_006695	GAGCUA, GGACU	2.68	1.99	3.28	2.65	1
SPRED2	200734	NM_181784	AUGUAA, CAAAG(	0.25	4	3.7	2.65	2
WDR40A	25853	NM_015397	CGUUU, CUCGAG	0.21	4.44	3.3	2.65	2
USP33	23032	NM_201626	GAAUAG, CAGCU(	-0.24	3.25	4.94	2.65	2
RKHD1	399664	NM_203304	UCGUGA, GCCGA	-0.71	2.13	6.53	2.65	1

ETV4	2118	NM_001986	GCUCAU, GGACUU	2.67	1.24	4.03	2.65	1
TARDBP	23435	NM_007375	AAUAUG, GGGCUU	0.45	3.59	3.9	2.65	2
GTF2I	2969	NM_001518	GUAGAA, GAUUGC	-0.53	4	4.47	2.65	2
MKRN2	23609	NM_014160	CCCUAA, ACUGUG	3.37	1.94	2.62	2.64	1
NQO1	1728	NM_001025434	GGCUUA, GAUGAC	1.82	2.08	4.02	2.64	1
AKAP6	9472	NM_004274	GGAUAG, CAUCAC	-0.26	3.52	4.66	2.64	2
HOXA9	3205	NM_152739	GUUCU, GUGAUG	-0.95	5.44	3.43	2.64	2
DHX35	60625	NM_021931	UCGGGAA, CAAUU	3.06	2.81	2.04	2.64	1
LOC151871	151871	NM_138815	GCGUUU, CGGGAC	1.36	3.93	2.62	2.64	1
LOC400566	400566	NM_001013672	CGGAGA, GGAAAU	0.33	4.56	3.02	2.64	2
MKRN3	7681	NM_005664	GCUAUU, AUUAUC	2.44	3.11	2.35	2.63	1
PCLKC	54825	NM_017675	GUAUA, GAGCCU	2.42	2.55	2.93	2.63	0
C6orf120	387263	NM_001029863	GACCUA, GGGUAG	-0.03	3.74	4.19	2.63	2
FLJ39660	284992	NM_001080539	CCGAAA, AAGCAA	-0.49	5.35	3.04	2.63	2
FLJ25067	149840	NM_152504	UGGAAU, AUUAAC	3.85	3.48	0.56	2.63	2
CDC2	983	NM_033379	CUAUUA, GUAUA	1	3.16	3.73	2.63	2
LOC129530	129530	NM_174898	GCACGA, CCAGGA	0.98	3.06	3.85	2.63	2
C21ORF7	56911	NM_020152	GGCGAU, UGAGAU	-0.12	3.81	4.18	2.62	2
PNMA3	29944	NM_013364	GAGAUA, GGCACA	3.99	2.42	1.45	2.62	1
TRIM6-TRIM34	445372	NM_001003819	GGGUUA, CGAUUU	1.57	3.6	2.69	2.62	1
ECG2	84651	NM_032566	GGGAAU, CCGAGA	0.25	4.54	3.07	2.62	2
OR1F1	4992	NM_012360	UAUAUG, GCACCA	-0.77	4.25	4.38	2.62	2
PRY	9081	NM_004676	UUUGAA, CCAAUU	2.48	3.97	1.4	2.62	1
GRAP	10750	NM_006613	GCCCGU, UAAACU	1.56	3.24	3.04	2.61	2
GPR172A	79581	NM_024531	AUUCUG, CAGGAA	1.59	3.87	2.38	2.61	1
EIF4A2	1974	NM_001967	GGGAUU, CUUUGU	0.59	2.75	4.5	2.61	1
ZCCHC2	54877	NM_017742	GUGUUA, AUAAAC	0.48	4.83	2.53	2.61	1
NEIL3	55247	NM_018248	GUGUAA, CUAUGU	0	4.75	3.09	2.61	2
HERC3	8916	NM_014606	GAAGUA, GCAAAG	1.4	2.24	4.19	2.61	1
OR5B3	441608	NM_001005469	GAGCUU, UCUGUA	1.22	3.31	3.3	2.61	2
RHO	6010	NM_000539	GUGUAA, GGUCAU	0.85	4.06	2.92	2.61	1
ARL10A	285598	NM_173664	GCAUUU, GCUUCA	3.89	3.73	0.2	2.61	2
POU2F1	5451	NM_002697	UGAUA, CCAACUC	2.72	1.64	3.46	2.61	1
HNMT	3176	NM_001024074	GUGCAU, CAUCUU	2.62	2.75	2.45	2.61	0

LOC136242	136242	NM_001008270	CCUCAA, CUGCUA	1.73	4.05	2.04	2.61	1
SALL4	57167	NM_020436	GAAUAA, CAAGAU	2.08	3.22	2.51	2.60	1
RAPSN	5913	NM_032645	GCAUUU, CAUUGC	1.57	2.22	4.02	2.60	1
PWWP1	154150	NM_138574	GGACGA, GGAUAC	0.1	4.14	3.57	2.60	2
ZC3H3	23144	NM_015117	CCGCCA, CAAUAA	1.99	2.87	2.93	2.60	0
JUNB	3726	NM_002229	GGGAUA, GAACAG	3.25	-0.01	4.55	2.60	2
MJD	4287	NM_030660	AUCCAU, GCACUA	3.13	3.52	1.14	2.60	2
PTTG1	9232	NM_004219	GUUUCA, GGGAAU	2.94	3.42	1.43	2.60	1
RAB28	9364	NM_004249	AUGUUA, GAGCAU	3.67	1.86	2.25	2.59	1
ABCC12	94160	NM_033226	GGCAAA, AGCAAU	2.17	4.32	1.29	2.59	1
NALP11	204801	NM_145007	GCACCA, GAUCAU	0.04	5.16	2.58	2.59	1
MRPL4	51073	NM_146387	CAGCAU, CCGUAC	1.7	2.84	3.23	2.59	1
COLEC12	81035	NM_130386	CGUUGA, GCCAAC	0.41	5.15	2.2	2.59	1
PXMP4	11264	NM_183397	GAAUCA, GCUGAC	0.23	5.08	2.45	2.59	1
DIP13B	55198	NM_018171	GCAGAA, GCGGAA	0.55	3.11	4.1	2.59	2
LOC283337	283337	NM_001004304	UCCUUA, AAGCAA	2.13	3.86	1.76	2.58	1
SULF2	55959	NM_198596	CAAUAA, GGUGCU	3.16	2.34	2.24	2.58	1
TRAF5	7188	NM_001033910	CCUAUA, GAGCAA	2.54	3.3	1.9	2.58	1
TNFRSF10C	8794	NM_003841	GCCCUA, CAAUGA	2.06	1.28	4.4	2.58	1
FLJ11000	55281	NM_018295	GGCAAC, AGACAG	1.71	3.51	2.52	2.58	1
FLJ34154	283450	NM_173813	ACGAGA, ACUGAA	1.53	3.83	2.38	2.58	1
FOXD2	2306	NM_004474	CACAUU, GCUGCC	0.35	4.49	2.9	2.58	1
OR5AY1	343170	NM_001004732	GAAUUU, UCUAC	2.41	3.21	2.11	2.58	1
BACE1	23621	NM_138973	UCAUUG, UAUGGC	2.2	1.57	3.96	2.58	1
C6orf190	387357	NM_001010923	GGUAGA, AUUGAA	0.8	3.05	3.87	2.57	2
CYB5	1528	NM_148923	CGAUUU, GAUGAC	0.36	2.93	4.43	2.57	1
SEMA3E	9723	NM_012431	GAUCGA, GAGAAU	-0.94	3.19	5.47	2.57	2
MGC34646	157807	NM_173519	CGAAUC, CAGCUU	4.93	0.56	2.22	2.57	1
CPZ	8532	NM_003652	GGGCUG, UGACA	2.75	1.92	3.04	2.57	1
E2F1	1869	NM_005225	AUGAGA, GAGCAG	1.79	3.45	2.46	2.57	1
TRAPPC3	27095	NM_014408	GGAUGA, CCCUAC	1.03	2.29	4.38	2.57	1
ARGFX	503582	NM_001012659	GGCAUA, CCUCCA	0.97	3.19	3.54	2.57	2
MRPL30	51263	NM_145213	GUCGUA, GAGAAC	0.09	3.38	4.23	2.57	2
PCTK2	5128	NM_002595	UGACUU, AAUGGA	2.68	3.15	1.86	2.56	1

C8ORF1	734	NM_004337	GUAUCA, UGUGGU	0.15	3.5	4.04	2.56	2
ZMYND12	84217	NM_032257	UCGCAU, ACUCUU	1.2	3.01	3.47	2.56	2
OR52B4	143496	NM_001005161	GGUAUG, CAAGAA	2.42	2.46	2.79	2.56	0
C10ORF57	80195	NM_025125	GCGCCA, CAUCACA	1.78	2.08	3.81	2.56	1
RBP4	5950	NM_006744	GGAGUU, GUGAGC	1.27	3.14	3.26	2.56	2
SET7	80854	NM_030648	GGAUUA, GGAGUG	0.71	2.31	4.65	2.56	1
ANKMY1	51281	NM_017844	GACGAAA, UCACCC	-0.79	2.64	5.82	2.56	1
FBXL12	54850	NM_017703	GUGUAA, AGACAG	2.07	1.72	3.87	2.55	1
FAM36A	116228	NM_198076	GGACUU, GGGAAG	1.3	4.16	2.2	2.55	1
IKBKE	9641	NM_014002	GACAUG, GCAUUG	-0.65	3.96	4.35	2.55	2
OSGEPL1	64172	NM_022353	CCGCUA, CUGGUG	4.85	2.72	0.08	2.55	1
KLK1	3816	NM_002257	GGAUUG, UGACAC	-0.97	5.43	3.19	2.55	2
NADSYN1	55191	NM_018161	CGGACA, UCUGCA	2.76	3.11	1.78	2.55	1
FLJ25200	151651	NM_144715	GGAUAA, GUUUAC	3.93	1.2	2.51	2.55	1
CACNA1C	775	NM_000719	UAUUUA, GGGUAC	3.01	2.08	2.55	2.55	1
SLC26A4	5172	NM_000441	GGAAUU, GCUAU	1.76	3.59	2.29	2.55	1
KIAA1219	57148	NM_020336	GAUUCA, CCUCGC	0.53	3.11	4	2.55	2
GAJ	84057	NM_032117	GAGCGA, CAAAGA	2.47	2.12	3.04	2.54	1
CDV-1	28981	NM_031473	CCGUAA, CCACAG	2.4	2.07	3.16	2.54	1
DAZAP2	9802	NM_014764	UAGGUU, UCAGAC	0.28	4.44	2.9	2.54	1
RIMS2	9699	NM_014677	GGCGUA, GCAUGU	1.98	3.1	2.54	2.54	1
CDC25B	994	NM_212530	GAAUA, UGGAUA	0.22	2.35	5.05	2.54	1
LOC256394	256394	NM_001080451	GUUAAU, CGGCAC	1.85	2.91	2.86	2.54	0
CDH17	1015	NM_004063	GUCAAA, GAACCG	2.34	2.68	2.59	2.54	0
SUPT3H	8464	NM_003599	GCGUAU, CAAACU	1.36	3.38	2.87	2.54	1
KIAA1715	80856	NM_030650	GCGGUA, ACGAUG	2.88	2.02	2.7	2.53	0
NALP5	126206	NM_153447	GAUUAA, GCGUGA	1.84	2.59	3.17	2.53	1
ARRDC1	92714	NM_152285	UCAGGCA, CCUAU	-0.19	4.13	3.66	2.53	2
FLJ10159	55084	NM_018013	GGUAAA, GCGACA	0.71	3.68	3.2	2.53	2
C2ORF27	29798	NM_013310	AUUUA, GAAAGU	3.62	3.59	0.38	2.53	2
PMS2L5	5383	NM_174930	CCAGGU, GCAAAA	3.34	1.65	2.6	2.53	1
MGC39520	196472	NM_153364	GUUCAA, CACCCAU	2.15	1.92	3.52	2.53	1
FLJ36870	285349	NM_173658	GCGAAU, GCAGUC	0.23	3.29	4.07	2.53	2
FLJ36198	219990	NM_173801	CGGAUGA, ACAUA	2.26	4.21	1.11	2.53	1

MGC4562	115752	NM_133375	UAUUCA, GAAGGA	1.33	3.9	2.35	2.53	1
BICC1	80114	XM_499560	GAGCGA, CCAGGU	0.61	2.39	4.58	2.53	1
CGI-90	51115	NM_016033	UGGAUA, GUAUG	1.63	2.63	3.31	2.52	1
RPL3	6122	NM_001033853	GGGCUA, GGAUC	-1.05	4.1	4.52	2.52	2
SPRY1	10252	NM_199327	GGAAUA, UAAGA	4.12	2.04	1.4	2.52	1
LOC148206	148206	NM_182515	GGGCAU, GGCUU	-2.09	7.35	2.3	2.52	1
OTOA	146183	NM_170664	AGGUUU, UAGCC	4.06	1.35	2.14	2.52	1
MGC26988	153745	NM_130899	GUUCAA, CUCUA	2.03	2.47	3.05	2.52	1
DAZ2	57055	NM_001005786	GGAUAU, GAAGU	-0.58	4.54	3.58	2.51	2
SLC6A1	6529	NM_003042	ACGUAU, UGGAGU	2.89	2.81	1.83	2.51	0
COBL	23242	NM_015198	UAACGA, CAAGA	1.36	2.88	3.29	2.51	1
HIST1H2AG	8969	NM_021064	AGCACA, UGAAAA	2.59	1.91	3.03	2.51	1
PHKB	5257	NM_001031835	GAGUUA, GAAGA	2.31	3.09	2.12	2.51	1
TAS1R2	80834	NM_152232	CCGUGU, GAUCAC	0.87	3.96	2.69	2.51	1
OGG1	4968	NM_016827	GGUGGA, GCUCAG	-0.17	4.04	3.65	2.51	2
TEX9	374618	NM_198524	CAGUUU, CGGCAA	2.95	2.91	1.65	2.50	0
PHEX	5251	NM_000444	AAGAUU, CCUACA	1.87	2.01	3.63	2.50	1
DKFZP434C245	25886	NM_015426	CUCGUA, UCAUG	1.44	3.13	2.94	2.50	1
LOC646424	646424	NM_001080525	AUCAAAC, ACUCU	0.09	5.18	2.24	2.50	1
HKR1	284459	NM_181786	GGUUUA, UGAAGA	2.83	1.68	2.99	2.50	0
KCNJ6	3763	NM_002240	GACUAA, GGAUAU	2.14	1.9	3.46	2.50	1
PLSCR1	5359	NM_021105	AAGAAG, CCAAAU	-0.68	4.02	4.16	2.50	2
FLJ10970	55273	NM_018286	CCCUAA, CAUCACU	2.9	1.99	2.6	2.50	0
LGI2	55203	NM_018176	CCGAAU, CCAAGA	2.63	2	2.86	2.50	0
ATP1B3	483	NM_001679	GGGUUA, CCCUUG	1.77	2.47	3.25	2.50	1
XTP7	90332	NM_138568	UGCGCGA, CGUCA	0.79	3.89	2.81	2.50	1
TPK1	27010	NM_022445	CCUUU, UAAGGG	3.44	3.13	0.91	2.49	2
KRT15	3866	NM_002275	AUCUAA, GGAGGU	-0.37	3.72	4.13	2.49	2
RAB9P40	10244	NM_005833	GUCUAC, CCACAG	-0.43	5.41	2.5	2.49	1
LOC340351	340351	NM_178563	GGAAUA, GAACAG	3.51	1.44	2.53	2.49	1
LOC124976	124976	NM_001124758	GGGAUU, ACCUCA	2.8	2.32	2.36	2.49	0
CYBRD1	79901	NM_024843	CGGCUU, CAGCUG	1.54	3.7	2.23	2.49	1
RNPC1	55544	NM_183425	AGGUAU, GAGAAU	0.6	3.27	3.6	2.49	2
FLJ22655	79785	NM_024730	GGAAUC, AAGGAA	1.22	2.66	3.59	2.49	1

RNF19	25897	NM_015435	CUGUAA, ACAAAU	0.84	3.1	3.53	2.49	2
KIAA0652	9776	NM_014741	GAGUUU, CCAUA(	-0.58	3.49	4.56	2.49	2
HNF4G	3174	NM_004133	CAGUAA, CCAAUC	-1.23	3.52	5.18	2.49	2
PDIR	10954	NM_006810	CGCUCA, CCAAAG	1.79	3.23	2.44	2.49	1
NPTX2	4885	NM_002523	AUGUG, AGGCAU	2.15	1.93	3.37	2.48	1
PIAS3	10401	NM_006099	GAAGUU, GGAAG(	2.4	2.48	2.57	2.48	0
OGFR	11054	NM_007346	ACAACUA, CCAAGU	1.61	3.52	2.31	2.48	1
NT5C1B	93034	NM_033253	GGGAAA, AGGUAU	0.25	3.68	3.51	2.48	2
ZNF583	147949	NM_152478	GUUCUA, GUGGAU	-1.42	3.24	5.61	2.48	2
FLJ13236	79962	NM_024902	GCGUAU, GCUUU	2.77	2.68	1.98	2.48	0
MGC16309	90506	NM_033413	AGCGAU, GUCUCU	-0.25	3.68	4	2.48	2
LOC131368	131368	NM_175056	GAUUAA, CUGCU(	2.78	2.61	2.03	2.47	0
AD031	83935	NM_032021	UAUUUA, CAGAU(	2.17	2.51	2.73	2.47	0
SLIT3	6586	NM_003062	CGACUA, AGACUA	0.96	2.83	3.62	2.47	1
SLC12A8	84561	NM_024628	ACGUUA, CAUACG	-0.02	3.32	4.11	2.47	2
MRC2	9902	NM_006039	GAUGGA, AAACUC	2.1	1.8	3.5	2.47	1
FLJ21415	79794	NM_024738	CGAAUA, AAGCAU	1.66	2.91	2.83	2.47	0
KRTAP1-1	81851	NM_030967	GACUUA, CCUAG(	3.82	2.27	1.3	2.46	1
FLJ21174	79921	NM_001006937	ACGUUA, CUCAU	2.38	3.91	1.1	2.46	1
TUFM	7284	NM_003321	GCUGUC, CGAGAU	2.15	3.26	1.98	2.46	1
KIAA1618	57714	NM_020954	CGGAAA, GCACAA	1.71	2.77	2.91	2.46	0
VMD2	7439	NM_004183	GAGUUC, CGUCGA	3.03	2.95	1.4	2.46	1
NSBP1	79366	NM_030763	GCCCAA, CCUUCAU	1.78	2.93	2.67	2.46	0
ZFYVE20	64145	NM_022340	GAGAAA, UCGAUA	0.77	2.43	4.17	2.46	1
FNDC3	22862	NM_014923	GGUAUA, GCCAG(	3.92	0.85	2.61	2.46	1
SBF1	6305	NM_002972	CGAUGA, GCGGA(	3.36	1.49	2.53	2.46	1
KCTD11	147040	NM_001002914	GACUAG, CAACCU	1.06	3.17	3.14	2.46	2
LGMN	5641	NM_005606	GGAAUA, GAACAG	2.65	1.67	3.05	2.46	1
FLJ35976	284618	NM_001039517	CGAUUA, CCUAGA	1.61	3.27	2.49	2.46	1
OR4C45	403257	NM_001005513	UGUCA, GGGAUU	0.23	4.89	2.25	2.46	1
MRPL54	116541	NM_172251	UAAAGA, CCCUGA	0.13	3.57	3.67	2.46	2
UTP14A	10813	NM_006649	GACCUA, AACCAU	-0.77	3.9	4.23	2.45	2
MUT	4594	NM_000255	CACAAAU, CGGAA(	3.03	3.09	1.24	2.45	2
RHD	6007	NM_016225	CAGAUU, CAUAAC	2.1	3.36	1.9	2.45	1

PKD1L3	342372	NM_181536	GAUUUA, CCCAAU	-0.84	3.4	4.8	2.45	2
MGC16028	112752	NM_001102564	GAAUUU, UGGACC	0.22	3.41	3.72	2.45	2
ROR1	4919	NM_005012	AAUAAA, CAUCAA	4.89	-0.64	3.1	2.45	2
FLJ33318	162461	NM_153229	GUCGCA, CCACAG	1.77	3.1	2.48	2.45	1
COX11	1353	NM_004375	UUUUA, GAGGUG	0.92	2.55	3.88	2.45	1
WNT10A	80326	NM_025216	UGCACU, GAGGAC	0.31	3.25	3.79	2.45	2
GALNT5	11227	NM_014568	GUCCCA, GCAAAG	3.05	2.24	2.05	2.45	1
INPPL1	3636	NM_001567	GUGUGA, CCAAGA	2.63	1.69	3.02	2.45	1
PIGS	94005	NM_033198	GGCGUC, UUUCAC	0.86	3.02	3.46	2.45	2
PCDHGC4	56098	NM_032406	GGACGU, UGUAGC	2.89	1.52	2.92	2.44	0
POU6F1	5463	NM_002702	AGUCUA, AAACGA	1.72	2	3.61	2.44	1
SUHW2	140883	NM_080764	CCCAUA, GCUCUU	0.81	4.34	2.18	2.44	1
MGC13204	83695	NM_031465	UGAAU, CGAGGA	3.72	1	2.6	2.44	1
SLC27A2	11001	NM_003645	GCUGUU, GGAUAU	3.29	1.7	2.33	2.44	1
TMEM22	80723	NM_001097600	UGCUAU, GGAUA	2.96	2.21	2.15	2.44	0
DKFZP564O123	25978	NM_014043	GAUCGA, UGAAGA	2.82	3.04	1.46	2.44	1
LOC284018	284018	NM_181655	UCGGCAA, CAAAAC	2.19	2.86	2.27	2.44	0
BCAT2	587	NM_001190	UGGGAA, GGGCA	0.53	2.71	4.08	2.44	1
GRIA2	2891	NM_000826	GGUUA, GAAUAA	-0.5	2.8	5.02	2.44	1
CYLN2	7461	NM_032421	GAAGAA, CGUAUC	2.48	3.37	1.46	2.44	1
NUBP2	10101	NM_012225	GUCCGA, GAGCUC	0.28	3.61	3.42	2.44	2
UQCRC2	7385	NM_003366	GUGUGA, UAAAGC	2.42	2.36	2.53	2.44	0
MAGEA4	4103	NM_001011550	CGUAUA, CCGUGA	1.43	3.75	2.12	2.43	1
XKRY	9082	NM_004677	GUGAUA, AGACAC	1.2	2.22	3.88	2.43	1
DMD	1756	NM_004019	CGACUA, CAAGAC	0.66	3.48	3.16	2.43	2
C18ORF26	284254	NM_173629	UGGAAA, GUACAA	2.91	2	2.38	2.43	0
IMP-3	10643	NM_006547	AGGUAA, GGAUUU	-0.43	4.06	3.66	2.43	2
KIAA0961	22835	NM_014898	CUAAUA, CAGCGA	4.13	0.7	2.45	2.43	1
RBM15	64783	NM_022768	CAUAUA, UAGCAC	2	1.37	3.91	2.43	1
FLJ11126	55308	NM_018332	GCGCAU, UGGAU	0.02	2.4	4.86	2.43	1
C21ORF127	29104	NM_182749	AGGUAA, GCAUCA	3.4	2.12	1.76	2.43	1
OTUD6B	51633	NM_016023	AAAUUA, CAUUAU	2.65	3.29	1.33	2.42	1
SPAG7	9552	NM_004890	GAGGAA, AGAUGA	0.95	3.72	2.6	2.42	1
PHC3	80012	NM_024947	CGGAAA, CUACAC	-0.35	4.31	3.31	2.42	2

BTBD4	140685	NM_025224	UGCACA, GAACGA	3.4	2.45	1.41	2.42	1
TSPAN13	27075	NM_014399	CGUACA, CUGUAU	3.38	0.7	3.18	2.42	2
HPS5	11234	NM_181508	UUAGA, GGAGUU	2.73	2.95	1.58	2.42	0
THAP4	51078	NM_015963	GGUUAA, GUGGA	2.21	1.45	3.6	2.42	1
CGI-96	27341	NM_015703	GUCGA, GGGUGG	1.99	2.75	2.52	2.42	0
GALNT1	2589	NM_020474	CGGACU, CGAAU	1.27	3.6	2.39	2.42	1
MGC29816	91782	NM_152272	CGCUAA, GGAGGU	2.84	2.43	1.98	2.42	0
QPCT	25797	NM_012413	UCAAAU, CCUUAA	1.68	2.86	2.71	2.42	0
TBC1D4	9882	NM_014832	GCUUCA, GGUGGA	0.9	3.46	2.89	2.42	1
FLJ12903	64766	NM_001017406	AAGAUA, GCUCAU	0.71	2.96	3.58	2.42	1
LOC90806	90806	NM_144567	GACAG, AGACAAU	0.42	2.43	4.39	2.41	1
AMOTL2	51421	NM_016201	GCAGUA, CAUAAA	0.03	4.19	3.02	2.41	2
FLJ22349	79879	NM_024821	GGCACU, CGAGGA	-0.51	4.18	3.57	2.41	2
DKFZP564C186	26155	NM_015658	AUCUGC, GAAUGG	3.29	1.91	2.03	2.41	1
GPRC5D	55507	NM_018654	AUGUUU, GCUCAU	3.12	0.8	3.31	2.41	2
LOC223075	223075	NM_194300	GAGGUA, GCAUCA	2.15	3.25	1.83	2.41	1
ZNF99	7652	NM_001080409	AGCAAU, CCUUAC	1.66	2.37	3.2	2.41	1
C18ORF37	125476	NM_194281	GCUUCU, CAGAGC	2.15	2.21	2.87	2.41	0
ZDHHC18	84243	NM_032283	GAACGA, GGAUUU	1.41	2.78	3.04	2.41	1
TAFA5	25817	NM_015381	AUGUUA, ACCACC	1.15	3.95	2.13	2.41	1
LOC93349	93349	NM_138402	CGGGUA, CCAAGA	0.51	4.22	2.49	2.41	1
APCS	325	NM_001639	CUAGUU, GGAGA	-0.47	3.89	3.8	2.41	2
KIAA0232	9778	NM_001100590	CGGAUU, CGUCAA	3.21	1.45	2.55	2.40	1
KRTAP5-2	440021	NM_001004325	GAGUGA, CUUAU	-0.05	3.65	3.61	2.40	2
TAFA2	338811	NM_178539	CCGGAU, UGACAU	3.33	1.6	2.28	2.40	1
SGCE	8910	NM_003919	CCUUUA, GCACAU	1.2	3.11	2.9	2.40	1
HBQ1	3049	NM_005331	CCGAGUA, CAACG	0.37	3.69	3.15	2.40	2
ATPIF1	93974	NM_016311	CGCCAU, AGAAGC	-1.16	5.55	2.8	2.40	1
IL6ST	3572	NM_175767	UACUAU, UAAAUA	3.38	2.43	1.38	2.40	1
KAB	9859	NM_014812	UUGGUA, CCUACA	3.03	1.75	2.41	2.40	1
PITPN	5306	NM_006224	CAUAUA, CGUGGA	2.36	1.52	3.31	2.40	1
NYD-SP25	89882	NM_001001875	ACCCUA, CCACUC	1.87	2.01	3.31	2.40	1
DKFZP434C212	26130	NM_015635	GCGUUA, AGAAUC	1.5	2.07	3.61	2.39	1
MYBBP1A	10514	NM_014520	CUAUUG, CGACUU	3.52	1.03	2.62	2.39	1

DKFZP434O0213	91355	NM_001135772	CCGGUA, CCUCAU	2.01	2.89	2.27	2.39	0
CD58	965	NM_001779	UAUGUG, GCACU	-0.61	3.07	4.71	2.39	2
KCND1	3750	NM_004979	ACCGAA, GCAAG	2	3.38	1.78	2.39	1
LOC148213	148213	NM_138286	GACACA, ACGUGA	1.08	3.27	2.81	2.39	1
TMEM91	641649	NM_001098825	GCCUUU, GCUGGA	0.22	3.06	3.88	2.39	2
ECRG4	84417	NM_032411	UCGAAA, UCAACU	-1.53	4.01	4.68	2.39	2
MGC4707	79096	NM_001003678	AGCCAAU, UCUCA	3.31	3.02	0.82	2.38	2
GBX2	2637	NM_001485	CAGCGA, GCGCU	0.46	4.13	2.56	2.38	1
MGC50273	408029	NM_214461	AUUUA, GAAAGU	2.83	2.15	2.16	2.38	0
FLJ10826	55239	NM_001031707	CAAGUU, GGGAAG	2.61	1.33	3.2	2.38	1
LOC91431	91431	NM_138698	CGCCAA, UUAAAU	2.35	2.72	2.07	2.38	0
ZNF336	64412	NM_022482	CCAAGA, CGGAUA	1.42	2.74	2.98	2.38	0
AMICA	120425	NM_153206	UCGUUA, GACCA	4.32	0.55	2.26	2.38	1
CTSF	8722	NM_003793	AUAUGA, GAGGA	1.68	2.67	2.78	2.38	0
LZTFL1	54585	NM_020347	AUCUUA, GGAAA	-0.81	3.19	4.75	2.38	2
ABCE1	6059	NM_002940	AAUUAC, CAAAG	3.35	3.15	0.62	2.37	2
C9ORF13	79987	NM_153366	GGGAAA, GGGCA	1.58	2.99	2.55	2.37	0
MIZF	25988	NM_198971	CGCAAA, GCAGGG	0.18	3.62	3.32	2.37	2
RAB41	347517	NM_001032726	GGAAUA, GGGUA	2.7	1.9	2.51	2.37	0
OR13J1	392309	NM_001004487	UAGAUA, CCAUG	2.36	1.69	3.06	2.37	1
CPA2	1358	NM_001869	GUCUAG, GUACA	2.85	2.62	1.63	2.37	0
EVC2	132884	NM_147127	AUCUAA, AGACGA	2.55	2.52	2.03	2.37	0
G6PC2	57818	NM_021176	ACAGUU, CUACCG	3.09	1.85	2.15	2.36	1
PPFIBP2	8495	NM_003621	CCGGAA, AUGCAG	0.74	2.19	4.16	2.36	1
MGC34648	199870	NM_152660	AGGAAA, UAUCCU	3.25	2.21	1.62	2.36	1
CNO	55330	NM_018366	UACGAA, AGUAUG	0.97	2.32	3.79	2.36	1
RSHL1	81492	NM_030785	GCGAAA, GACGAG	1.97	2.41	2.69	2.36	0
GPR37	2861	NM_005302	UAAUAU, GAAAG	-0.19	2.55	4.71	2.36	1
PABPC1	26986	NM_002568	UAAUUU, GGACA	2.15	2.79	2.13	2.36	0
MYST2	11143	NM_007067	UGAUUA, UAGGA	3.85	2.27	0.94	2.35	1
LOC55908	55908	NM_018687	GGAUAU, GAAUCU	-1.54	4.9	3.7	2.35	2
PGBD2	267002	NM_001017434	GAGACA, CCAAAU	2.3	2.22	2.54	2.35	0
LOC389708	286319	NM_001004125	GAUCUU, AGGCC	-0.51	3.53	4.03	2.35	2
HBP1	26959	NM_012257	GAUGUA, GCAAAU	1.94	2.98	2.12	2.35	0

C19ORF15	57828	NM_021185	ACGAAA, AGAUGA	1.77	3.14	2.13	2.35	1
GUCY2C	2984	NM_004963	AGACUU, CGGAUG	0.18	4.43	2.43	2.35	1
ADORA2A	135	NM_000675	GAUUCA, GCCAU	0.66	2.74	3.63	2.34	1
DNASE2	1777	NM_001375	AAGAUC, GAACUA	1.17	3.64	2.21	2.34	1
GJA3	2700	NM_021954	CAACAU, UGUACC	0.14	2.65	4.23	2.34	1
KCNC1	3746	NM_004976	GAACGU, CCAACA	0.39	2.5	4.13	2.34	1
FLJ20366	55638	NM_017786	UCGUCA, GCACAG	-0.4	3.59	3.83	2.34	2
CES3	23491	NM_024922	UGACGUA, AAUUC	3.39	2.91	0.71	2.34	1
CYLC1	1538	NM_021118	AGGAAA, CAGAGA	2.2	2	2.81	2.34	0
GAGEC1	9506	NM_007003	AAAGUA, CCUAAC	0.73	3.78	2.5	2.34	1
KLHDC3	116138	NM_057161	ACAUUU, CCUACA	-0.02	4.97	2.06	2.34	1
RNF150	57484	NM_020724	CUUUUA, CCAAUG	3.42	0.25	3.33	2.33	2
CHRM2	1129	NM_001006629	AAACGA, CCAUUA	3.16	1.76	2.08	2.33	1
PAX6	5080	NM_001604	AGUAAA, GUGCGA	3.13	-0.43	4.3	2.33	2
SIGLEC10	89790	NM_033130	CGGCAU, UCAUGA	3.02	2.31	1.67	2.33	1
NOR1	127700	NM_206837	GCUCUA, GCUUU	1.59	2.9	2.51	2.33	0
KIAA0478	9923	NM_014870	CUGAUA, GCAGUG	1.19	3.69	2.12	2.33	1
NECAP2	55707	NM_018090	UCCGCAU, GCCAAA	0.75	4.14	2.11	2.33	1
IGSF6	10261	NM_005849	CCGCAA, GGUACC	2.05	2.15	2.8	2.33	0
EIF2C1	26523	NM_012199	GACUUU, UGACAA	2.4	2.06	2.53	2.33	0
FLJ10055	55062	NM_017983	GGACGU, CAAACU	0.22	2.36	4.41	2.33	1
CDC14B	8555	NM_003671	GUGACA, GUACAU	-0.35	2.1	5.24	2.33	1
COX6B2	125965	NM_144613	GAAUAA, GCCAGA	-0.46	4.92	2.53	2.33	1
LAMC3	10319	NM_006059	AGAUGU, GAACGC	-0.52	3	4.51	2.33	2
ARHE	390	NM_005168	CAAUCA, CAGCAA	-0.99	2.88	5.1	2.33	1
LOC126017	126017	NM_001004301	UACUCA, GUCAAC	1.35	3.12	2.5	2.32	1
FLJ12949	65095	NM_178159	GGGAUU, AAGAGA	2.04	2.72	2.21	2.32	0
DLGAP4	22839	NM_183006	GGAACA, CAUCAU	2.06	2.55	2.36	2.32	0
SLCO2A1	6578	NM_005630	ACGUGA, GAAGGC	2.19	3.25	1.52	2.32	1
ZNF35	7584	NM_003420	UAUUCA, CCAAGA	2.18	2.4	2.38	2.32	0
RAI	10848	NM_006663	CGGAAA, GCAGAC	1.63	3.14	2.19	2.32	1
CPLX1	10815	NM_006651	CGUGUU, CGUGUU	-0.14	3.19	3.92	2.32	2
CCRK	23552	NM_012119	CAGUAU, GAACAG	2.02	2.8	2.13	2.32	0
COQ7	10229	NM_016138	CGGGCA, GCACUU	1.25	2.74	2.96	2.32	0

FLJ16636	158431	NM_001001662	GACAUA, CUAAGG	1.11	3.7	2.14	2.32	1
LOC159090	159090	NM_145284	CGGACU, GGGAA	0.88	4.03	2.04	2.32	1
RASAL1	8437	NM_004658	UGCGAU, GGCAA	3	2.16	1.78	2.31	1
SSNA1	8636	NM_003731	GAGGAG, CCGCCU	3.02	2.17	1.75	2.31	1
ABC1	63897	NM_022070	CGGCUA, GGAAG	2.68	2.44	1.81	2.31	0
RNF166	115992	NM_178841	UGGAAA, GGACU	2.54	2.88	1.5	2.31	0
FLJ38663	91574	NM_152269	UCGGAA, GGGAG	2.44	2.37	2.11	2.31	0
C10ORF97	80013	NM_024948	GCAUUA, AAGUCU	2.1	2.01	2.81	2.31	0
OR6C3	254786	NM_054104	UGGUUU, UAACA	0.31	2.18	4.43	2.31	1
A4GALT	53947	NM_017436	AGUUCG, AGAAA	0.05	2.99	3.88	2.31	1
MGC31963	112770	NM_144580	AAUUAA, GCACC	3.55	-0.27	3.63	2.30	2
CAST	831	NM_173061	AGUCUA, GCGAA	2.41	1.79	2.71	2.30	0
FLJ11752	92344	NM_152281	GGCUAA, CAACAA	2.63	2.13	2.14	2.30	0
LOC123169	123169	NM_138792	GGUGCA, GCCGG	-0.96	4.34	3.52	2.30	2
ZNF607	84775	NM_032689	AUAUCA, CUUUA	2.1	3	1.8	2.30	1
SOX12	6666	NM_006943	GAUUA, GGGCGA	0.35	3.25	3.29	2.30	2
MKNK1	8569	NM_198973	AGUGA, GGAGUA	2.65	1.53	2.71	2.30	0
SV2A	9900	NM_014849	GUGUAC, AAGGG	0.36	2.03	4.5	2.30	1
DKFZP586A0522	25840	NM_014033	UAAUUA, AGUGU	1.72	2.81	2.35	2.29	0
BTG3	10950	NM_006806	UUCGUU, GAGAG	1.46	3.14	2.28	2.29	1
MGC14560	51184	NM_016301	GACUAU, CCUCUA	0.52	3.92	2.44	2.29	1
IMP4	92856	NM_033416	GGUCGA, GCAAU	2.16	2.71	2	2.29	0
SERPINE1	5054	NM_000602	GAUCGA, UCUCC	1.11	3.38	2.38	2.29	1
GLTP	51228	NM_016433	CGGUGA, GCGGC	0.54	3.38	2.95	2.29	1
LRP10	26020	NM_014045	GACUAC, GCCAGG	2.3	1.97	2.6	2.29	0
NR1H4	9971	NM_005123	CGUCAU, CCAGA	1.16	2.64	3.07	2.29	1
CAPS	828	NM_080590	CUACUA, GUGAG	-0.31	2.42	4.76	2.29	1
PAPOLG	64895	NM_022894	CGCCAU, GCAAA	-0.61	5.31	2.17	2.29	1
HTR1F	3355	NM_000866	UUGUGA, GCAAA	4.37	0.34	2.15	2.29	1
MGC3248	84516	NM_032486	CGCCUU, GUGCU	2.71	2.03	2.12	2.29	0
OTUB2	78990	NM_023112	GAUUAA, GAGCAG	0.08	3.86	2.92	2.29	1
RPL30	6156	NM_000989	GGGGUA, GACAA	-0.34	3.68	3.52	2.29	2
MLF1	4291	NM_022443	GCCAUGA, CAAAC	2.58	-0.46	4.73	2.28	1
TPP2	7174	NM_003291	AUUAUA, GAGUAA	1.28	2.66	2.91	2.28	0

BAAT	570	NM_001701	AGUUU, GACUAU	1.77	2.39	2.69	2.28	0
PCDHB13	56123	NM_018933	GCGUUU, GAGCAA	3.07	1.58	2.19	2.28	1
ARHGEF4	50649	NM_015320	CUACUC, CAACGG	0.62	3.5	2.72	2.28	1
HSPE1	3336	NM_002157	GUCGCU, CCACUG	0.52	2.77	3.55	2.28	1
PRKCQ	5588	NM_006257	AAGUCU, GCAAUU	-0.18	3.5	3.52	2.28	2
KCTD13	253980	NM_178863	CGUGA, GAGGUU	-0.49	4.43	2.9	2.28	1
CKAP1	1155	NM_001281	GAGAUA, UGUAUC	1.77	2.3	2.76	2.28	0
FLJ20811	54470	NM_001009584	GACUUA, AAGAGG	1.46	2.28	3.09	2.28	1
CCDC50	152137	NM_174908	GAGAUU, GCAGAC	0.27	2.6	3.96	2.28	1
MONDOA	22877	NM_014938	GUUUAU, GAAGAC	-1.19	3.23	4.79	2.28	2
MGC20419	112970	NM_138417	GGUUUA, CAGAGC	3.03	1.73	2.06	2.27	1
GAGE8	100101629	NM_012196	GUGUGA, GAUGA	2.82	2.33	1.67	2.27	0
EOS	260429	NM_152891	GCUGCU, CAACCU	1.74	2.96	2.12	2.27	0
HCN3	57657	NM_020897	UAACUU, GGCCA	0.74	2.43	3.65	2.27	1
ARSE	415	NM_000047	CCAACA, ACGAGU	0.36	3.16	3.3	2.27	2
G3BP	10146	NM_198395	ACAGUA, GAAGG	2.27	-0.01	4.55	2.27	1
FABP3	2170	NM_004102	GGGAAA, ACACAC	1.46	3.19	2.16	2.27	1
PRY2	442862	NM_001002758	UUUGAA, CCAAUU	1.27	3.31	2.23	2.27	1
SLC9A7	84679	NM_032591	AAUGA, UCUUUG	2.03	2.12	2.65	2.27	0
C6ORF60	79632	NM_024581	GAGCUA, CCAAGA	1.18	2.65	2.97	2.27	0
MXRA7	439921	NM_198530	AAGAAG, CAUCAU	0.16	3.79	2.85	2.27	1
EPLIN	51474	NM_016357	GCGUAA, AAAUGA	2.01	2.63	2.16	2.27	0
KIAA0146	23514	NM_001080394	GGUGUA, GAAGUC	2.59	1.43	2.78	2.27	0
METTL1	4234	NM_023033	GGACAA, GGAAUC	2.71	1.42	2.66	2.26	0
HYDIN	54768	NM_017558	GCCUUA, AGAUUC	1.83	2.46	2.5	2.26	0
STARD13	90627	NM_052851	CCGGUA, GCUUAU	-0.82	3.4	4.21	2.26	2
KIAA1012	22878	NM_014939	GAAGAA, CUCCAU	2.41	2.54	1.83	2.26	0
STATIP1	55250	NM_018255	CAUUGU, UCACAA	0.49	4.13	2.16	2.26	1
SFRS7	6432	NM_001031684	CCUAGA, CGACGU	2.51	2.77	1.5	2.26	0
ORMDL2	29095	NM_014182	UGGGUA, GUGUA	4.23	2.89	-0.35	2.26	1
TTC8	123016	NM_198310	UUACAA, GCGGAA	1.46	2.21	3.1	2.26	1
FLJ20202	54855	NM_017709	GAGAUA, UCAAAA	1.32	2.38	3.07	2.26	1
TPST2	8459	NM_003595	GGUCUA, UGUCCA	2.01	3.17	1.58	2.25	1
MXD4	10608	NM_006454	UAGAAA, GGACAU	0.66	3.79	2.31	2.25	1

SORCS3	22986	NM_014978	UGUCAA, CUAUGC	-0.23	3.12	3.87	2.25	2
TSPAN11	441631	XM_940107	GGUUCU, CAGCAG	-0.67	3.17	4.26	2.25	2
LOC139886	139886	NM_001012968	GCAUUA, GUUUA	2.67	2.71	1.37	2.25	0
NMUR1	10316	NM_006056	CUACUA, GGGCGU	2.16	2.29	2.3	2.25	0
ADCK4	79934	NM_024876	AGCAUU, UCACAG	2.01	1.04	3.7	2.25	1
DHRS10	51171	NM_016246	AUCGUG, GCAUUC	2.13	3.9	0.72	2.25	1
ZNF206	84891	NM_032805	GGGCGU, GAGGAC	2.86	2.39	1.49	2.25	0
APOBEC1	339	NM_005889	GCAUGA, UAAAGA	0.49	2.42	3.83	2.25	1
LOC285527	285527	NM_001039751	ACGGAA, GAUGAA	-1.07	4.11	3.7	2.25	2
HRNR	388697	NM_001009931	GGAGUG, CGUCAA	3.03	2.68	1.01	2.24	1
NDUFA8	4702	NM_014222	AAAUUA, GUGCAG	1.37	3.22	2.12	2.24	1
ACTR1B	10120	NM_005735	GUGCUA, CGGCUG	-1.63	4.56	3.78	2.24	2
UBE4B	10277	NM_006048	CAGAUU, GCUAAA	4.12	3.3	-0.72	2.23	2
SLC9A3R2	9351	NM_004785	CACUUC, GGAAGG	3.57	1	2.13	2.23	1
OR5AK2	390181	NM_001005323	UGAAUU, CAGCAU	-0.01	2.89	3.82	2.23	1
RDH10	157506	NM_172037	CGACUA, GUUCAG	-0.78	2.93	4.55	2.23	1
FLJ14803	84928	NM_032842	GACUUC, AUAAUA	3.32	2.75	0.62	2.23	1
HPCA	3208	NM_002143	GGGAAA, CGCCAA	3.14	2.75	0.8	2.23	1
CASP4	837	NM_001225	GACAAA, GAACUC	2.27	1.48	2.94	2.23	0
DDAH2	23564	NM_013974	GGCAAC, GAGCUC	1.27	2.36	3.06	2.23	1
RODH-4	8608	NM_003708	UGGCGA, CUGAGC	0.16	3.28	3.25	2.23	2
CD8A	925	NM_171827	ACCUGG, CGAGAU	-0.2	3.19	3.7	2.23	2
C6ORF129	154467	NM_138493	CACAAA, UCUAGA	3.11	0.75	2.82	2.23	1
OPN1SW	611	NM_001708	CAUGUU, GUAGGA	2.95	2.89	0.84	2.23	0
LNPEP	4012	NM_175920	GGAAUA, UAGCAC	1.53	2.11	3.04	2.23	1
HFE2	148738	NM_213652	GAUUCA, GCCUAG	0.98	2.41	3.29	2.23	1
AP1S2	8905	NM_003916	AGCACA, GGAAU	0.72	2.35	3.61	2.23	1
KIAA1847	84619	NM_181485	GGACGA, CCGAUG	0.5	3.63	2.55	2.23	1
UNC5A	90249	NM_133369	CGAGAA, CGGCUG	3.12	2.01	1.54	2.22	1
TAF2	6873	NM_003184	AGGCUAU, CCAAG	-0.46	3.24	3.89	2.22	2
OR1K1	392392	NM_080859	GAGGGA, GCGAAG	-1.69	4.03	4.33	2.22	2
MGC12466	93474	NM_033213	GAAGUU, UGUUGC	1.22	2.78	2.66	2.22	0
ZNF286	57335	NM_001130842	GGAAAU, CUGACU	-0.36	4.07	2.95	2.22	1
ERAF	51327	NM_016633	GAGUCA, AGACAA	-0.73	3.17	4.22	2.22	2

SLC4A5	57835	NM_021196	GGAUCA, GGACCC	2.01	3.86	0.79	2.22	1
OVCA2	124641	NM_080822	UCAAGU, GAGCAG	1.9	2.46	2.3	2.22	0
IL20RB	53833	NM_144717	UGUUU, AGGCAG	2.81	2.59	1.25	2.22	0
ZNF331	55422	NM_018555	GACUUA, GAAAUA	1.2	2.38	3.07	2.22	1
TAF5	6877	NM_006951	UAAGAU, GCAUCA	1.63	2.96	2.05	2.21	0
PAFAH1B3	5050	NM_002573	CGGACA, GCAGGU	-1.12	3.86	3.9	2.21	2
LOC340843	340843	NM_001013629	GUUAUA, GCAAAU	-1.61	2.73	5.5	2.21	1
LAIR1	3903	NM_021708	UCUAUU, GCAUGC	2.68	2.66	1.26	2.20	0
LOC113179	113179	NM_138422	CACAUA, GGCUAC	2.6	0.54	3.46	2.20	1
EIF1AY	9086	NM_004681	GUUUAA, GAAAU	-0.46	4.49	2.57	2.20	1
OXCT1	5019	NM_000436	GAUUUA, GCAGA	3.24	2.48	0.87	2.20	1
HSPC138	51501	NM_016401	ACGAAU, CCAUGA	2.33	1.15	3.11	2.20	1
C7ORF28A	51622	NM_015622	CAAUAA, GCUCAU	0.54	3.05	3	2.20	2
SCOC	60592	NM_032547	GAAUGA, GGAAAU	2.92	0.88	2.78	2.19	0
ADAMTS2	9509	NM_021599	CGCUUU, GCAAGG	2.25	3.27	1.06	2.19	1
PRKAG2	51422	NM_016203	AUGGUA, GAAUAC	2.25	1.55	2.78	2.19	0
MACF1	23499	NM_012090	UGAUAA, AAAAGU	2.18	1.84	2.55	2.19	0
PHOX2A	401	NM_005169	UUCGUA, GGACGA	1.95	2.1	2.52	2.19	0
H3F3A	3020	NM_002107	GACGUA, GCAACU	2.07	1.41	3.07	2.18	1
C20ORF46	55321	NM_018354	GAACUA, UGGAAC	0.42	4.1	2.03	2.18	1
DDIT4L	115265	NM_145244	GGCUUU, UGAGAA	3.76	2.36	0.42	2.18	1
MAEA	10296	NM_005882	GACGUG, GAACUC	1	3.14	2.39	2.18	1
PSCD2	9266	NM_004228	UUGAAC, GUAAGA	3	0.74	2.79	2.18	1
PLD2	5338	NM_002663	CUAUAU, CAAGGU	2.39	1.9	2.24	2.18	0
C9ORF18	254956	NM_198469	CUAUAA, GGCAUU	-0.94	2.07	5.4	2.18	1
TBX5	6910	NM_080718	GGCUAA, CACAAG	1.26	2.99	2.28	2.18	0
PHYHIP	9796	NM_014759	GAGCGA, GCGUCA	1.13	2.26	3.14	2.18	1
CHST4	10164	NM_005769	GGUAA, GCUACC	0.55	3.21	2.77	2.18	1
GALNT4	8693	NM_003774	CGGAUA, GUUAUA	-0.23	4.26	2.5	2.18	1
LOC345557	345557	NM_001005473	GGAUAA, CAGUGA	-0.54	4.35	2.72	2.18	1
EDG6	8698	NM_003775	CUAUUG, GCACAC	3.56	2.36	0.6	2.17	1
DERL1	79139	NM_024295	ACGUACA, CAACAA	0.17	3.79	2.56	2.17	1
FLJ12644	65251	NM_023074	GCGGAA, GUGAAC	0.76	2.29	3.46	2.17	1
EIF3S5	8665	NM_003754	GCAGUA, GCACAA	2.47	2.75	1.28	2.17	0

LGR6	59352	NM_001017404	JGUGUA, GGAAAU	2.07	2.71	1.72	2.17	0
NOS2A	4843	NM_153292	JAGUUU, GCUGCA	0.39	3.86	2.25	2.17	1
FLJ20265	54872	NM_017733	GAAUCA, CAGCUC	4.07	2.4	0.02	2.16	1
CHRAC1	54108	NM_017444	AGGAA, GGACGU	1	2.4	3.09	2.16	1
DHRS8	51170	NM_016245	AUUUAC, CUGGUU	4.21	2.06	0.22	2.16	1
ADSL	158	NM_000026	AUCAUC, GAGAUG	2.5	1.84	2.14	2.16	0
WDR67	93594	NM_145647	UCGAUA, GCAGAU	-0.16	2.69	3.96	2.16	1
COX4I2	84701	NM_032609	GCGCUA, GCACAC	3.23	3.2	0.05	2.16	2
LOC51760	51760	NM_016524	ACGAGU, GAUAUU	2.48	2.33	1.66	2.16	0
KIAA1449	57599	NM_020839	CGUGAA, ACACAU	1.63	2.66	2.18	2.16	0
MGC16044	91523	NM_138371	ACAUAA, GCACAA	1.48	2.44	2.55	2.16	0
PAK2	5062	NM_002577	GCAGUA, ACAGUG	-0.19	3.62	3.04	2.16	2
FLJ11773	60673	NM_021934	GGUCAA, GGUGAC	-0.37	3.92	2.92	2.16	1
C18ORF21	83608	NM_031446	UCGAAA, UCUUAA	2.18	3.23	1.05	2.15	1
OTX2	5015	NM_172337	GGAUAU, CAGCAC	1.46	2.82	2.18	2.15	0
LOC150297	150297	NM_001010859	GCACGA, GGUUAA	2.62	2.03	1.8	2.15	0
C19ORF4	25789	NM_012109	UGUGA, GCACAAC	0.03	2.8	3.62	2.15	1
CDY	203611	NM_001001722	CAGAUA, CCGAAA	-0.56	2.86	4.15	2.15	1
RALBP1	10928	NM_006788	GGAGUA, AAAGAC	-1.19	3.97	3.66	2.15	2
POU3F3	5455	NM_006236	GAGCGA, GCUCUA	3.03	2.06	1.35	2.15	1
LAMA5	3911	NM_005560	CAGCGA, AGUCCG	0.84	2.92	2.68	2.15	0
CHPPR	9650	NM_014637	GCGAAA, ACAUAA	0.57	2.43	3.44	2.15	1
NIP	90527	NM_144565	GAGUAU, CGGUGC	-1.52	4.33	3.63	2.15	2
TERF1	7013	NM_003218	JUGAUA, AGAGUA	2.18	1.76	2.49	2.14	0
DGKB	1607	NM_145695	CGAAUA, CGUCAC	2.41	2.1	1.92	2.14	0
ZNF696	79943	NM_030895	GAGUCA, UGGUG	0.34	2.92	3.17	2.14	1
FLJ31952	146857	NM_144682	CGAGUA, GAUAAU	1.1	3	2.32	2.14	1
PLEKHG2	64857	NM_022835	CAAUA, UGACAG	0.23	3.18	3.01	2.14	2
NUP155	9631	NM_004298	CGUGUA, ACAUUC	0.2	2.85	3.37	2.14	1
CNTN4	152330	NM_175613	CAGCGA, GCAGUG	1.4	2.06	2.95	2.14	0
CD59	966	NM_000611	UGUAA, GUGUGA	1.35	2.28	2.78	2.14	0
CAGLP	163688	NM_138705	CGACUA, GGAACA	-0.22	2.54	4.09	2.14	1
RIOK1	83732	NM_153005	JAUCUC, GGAGGC	-1.2	2.81	4.8	2.14	1
RUVBL2	10856	NM_006666	GGAGUA, GAAACC	-1.72	3.18	4.95	2.14	2

SPOP	8405	NM_001007228	GAGUGC, CAACUA	2.05	2.82	1.53	2.13	0
FLJ25421	150350	NM_152512	GGGAAA, ACAAGA	1.41	2.58	2.41	2.13	0
PRB3	5544	NM_006249	AGGACA, GUAACA	-0.34	3.46	3.26	2.13	2
DSG1	1828	NM_001942	AUUAG, UUGAUG	0.72	2.23	3.42	2.12	1
POLE2	5427	NM_002692	GUAAAU, AGAUG	0.08	3.02	3.27	2.12	2
LOC387921	387921	NM_001017370	AUUAUA, CAGGCU	3.44	0.3	2.63	2.12	1
UGT1A6	54578	NM_205862	UACUUG, CGUGAU	2.25	3.2	0.91	2.12	1
FLJ20071	54808	NM_017653	GGCACA, AUGACU	2.2	1.81	2.34	2.12	0
TRPC6	7225	NM_004621	GAUUAU, CAUCAU	2.06	2.85	1.44	2.12	0
UBE2Q1	55585	NM_017582	CACAAC, CGACCU	0.25	2.7	3.4	2.12	1
C6ORF89	221477	NM_152734	GAGCUU, ACUGGA	-0.71	3.94	3.12	2.12	2
UTS2	10911	NM_006786	GUGAGA, GGAUUU	4.79	-0.47	2.02	2.11	1
RBBP4	5928	NM_005610	UAGAA, GCUAUG	2.85	1.35	2.14	2.11	0
DT1P1A10	90121	NM_058163	GAGAAU, GGAGCU	1.96	2.32	2.06	2.11	0
LOC90167	90167	NM_194277	GCGUCU, GUUCCA	1.32	2.49	2.53	2.11	0
AHCY	191	NM_000687	GGAGUA, CAAGGU	1.11	2.35	2.88	2.11	0
BVES	11149	NM_007073	AGAUUA, GCUGGU	-0.28	3.36	3.26	2.11	2
FLJ20010	54494	NM_019021	CAAAUA, CCGCCA	3.8	0.35	2.18	2.11	1
SLITRK4	139065	NM_173078	CGUCAA, UGAGAA	2.07	1.94	2.32	2.11	0
RRM1	6240	NM_001033	UUCAUU, UGGAA	1.34	2.16	2.83	2.11	0
SOX17	64321	NM_022454	GUAUA, GAGGUG	1.3	2.86	2.17	2.11	0
LOC51333	51333	NM_016643	UCGCACA, UAUAC	-1.15	4.43	3.05	2.11	2
PCNT	5116	NM_006031	CUGAAG, CAGCAC	-0.07	2.73	3.65	2.10	1
OR7G3	390883	NM_001001958	AUGUCA, CUGCUC	2.39	3.7	0.22	2.10	1
CRISPLD2	83716	NM_031476	GGGUAU, GCAAAU	0.38	2.34	3.59	2.10	1
FZD7	8324	NM_003507	UCAUA, UGAUGU	-0.71	5.02	2	2.10	1
DERP6	23587	NM_203415	CAUAUA, GAGGAA	2.25	1.75	2.3	2.10	0
ARFRP1	10139	NM_003224	GUGUCA, UGAAGU	2.05	2.41	1.84	2.10	0
CNTNAP4	85445	NM_138994	GCGGAU, CAAAUU	1.65	2.22	2.43	2.10	0
GAL	51083	NM_015973	GCGGUC, AGAAUC	1.45	2.83	2.02	2.10	0
PPYR1	5540	NM_005972	CUGUUA, UGGAGU	2.84	2.15	1.3	2.10	0
BCAS3	54828	NM_017679	CGUCUA, CCAAGA	0.54	3.59	2.16	2.10	1
EFNA4	1945	NM_182689	UUUGUA, CAUCAC	-0.6	3.72	3.17	2.10	2
C2ORF15	150590	NM_144706	CAGUUA, CCUUGG	2.7	2.17	1.41	2.09	0

ACAS2L	84532	NM_032501	GGUGA, CCGCAG	2.15	1.04	3.09	2.09	1
PABPC5	140886	NM_080832	UGCGAU, GAUGA	1.35	2.01	2.92	2.09	0
FZD8	8325	NM_031866	CAAUCA, UCACCGU	0.54	2.12	3.62	2.09	1
NDUFS5	4725	NM_004552	GUUAUA, AAUAAA	-0.25	2.2	4.33	2.09	1
ORF1-FL49	84418	NM_032412	UGCUAA, ACUAU	-0.38	4.27	2.39	2.09	1
SPTB	6710	NM_000347	CAGAAA, GUUAAU	4.18	2.32	-0.23	2.09	1
CLK1	1195	NM_001024646	UGAGUA, GAGAA	0.25	2.58	3.44	2.09	1
PSAPL1	768239	NM_001085382	AGCGUU, UUGAGU	2.85	2.26	1.15	2.09	0
KRTAP13-2	337959	NM_181621	GGUUA, GAAACA	0.83	3.16	2.27	2.09	1
LOC56920	56920	NM_020163	AGUCAU, AGGACA	2.68	2.46	1.11	2.08	0
LIN7B	64130	NM_022165	GAGCUA, AGGGUG	0.61	3.28	2.36	2.08	1
GNPDA1	10007	NM_005471	CCUACA, CAGGUG	0	3	3.24	2.08	2
PALMD	54873	NM_017734	AAUGAA, GGACA	-0.53	3.18	3.59	2.08	2
ZNF350	59348	NM_021632	GCAUAA, UCAUA	2.53	2.37	1.33	2.08	0
RNF180	285671	NM_178532	GCCUAA, CCAAGA	2.52	0.95	2.76	2.08	0
IL19	29949	NM_153758	UCCUGU, CCAAGA	-0.17	2.05	4.35	2.08	1
DSCR9	257203	NM_148675	AUGUCUA, GUUAG	-1.05	4.4	2.88	2.08	1
ODC1	4953	NM_002539	UGUAUU, ACAAAU	-1.4	4.84	2.79	2.08	1
FLJ22344	79772	NM_001002796	CAACGA, GAAAGU	1.25	2.81	2.16	2.07	0
GLI	2735	NM_005269	GUGUAA, GGACGA	1	3	2.22	2.07	1
RAD54L	8438	NM_003579	GACUUU, GCACGA	-0.46	3.96	2.72	2.07	1
LOC339768	339768	NM_194312	CUAGUA, GGACA	-0.27	3.36	3.13	2.07	2
OR4F5	79501	NM_001005484	UGGAAA, CAACG	0.41	2.91	2.89	2.07	0
NETO1	81832	NM_138999	AAGUAU, ACGUU	3.99	2.18	0.04	2.07	1
INHBA	3624	NM_002192	CAUAGA, GGAUU	1.06	2.49	2.65	2.07	0
FLJ21986	79974	NM_001105533	GCGUAU, CUCAAU	0.23	2.31	3.66	2.07	1
P2RX4	5025	NM_175567	CGCAAA, GGAAAA	0.75	2.26	3.19	2.07	1
MAGEB4	4115	NM_002367	CGGCAU, UGAUA	2.15	3.03	1.01	2.06	1
C9ORF19	152007	NM_022343	UGGGCA, GUGGCA	1.46	2.04	2.68	2.06	0
TWSG1	57045	NM_020648	GCGAGU, ACACUA	0.12	2.71	3.35	2.06	1
LOC284361	284361	NM_206538	GGACUA, AGUCCU	3.45	0.63	2.09	2.06	1
LOC199675	199675	NM_174918	GAGUUA, GAAUGA	1.28	2.27	2.62	2.06	0
AGRN	375790	NM_198576	UUGAA, GCACGU	0.83	2.84	2.5	2.06	0
SDC1	6382	NM_002997	ACGGUA, CAUCAG	-0.82	3.72	3.27	2.06	2

MTF1	4520	NM_005955	UUCUUA, GGAAG	2.46	2.15	1.55	2.05	0
MYH4	4622	NM_017533	CUCAUC, CUAGAG	3.11	-0.01	3.05	2.05	2
FAM57B	83723	NM_031478	JAUUUA, UCUCAG	2.14	2.75	1.26	2.05	0
TCP1	6950	NM_001008897	GCAAUG, AUGAUC	-0.86	2.77	4.24	2.05	1
C11ORF16	56673	NM_020643	GUUAGA, CAUGGU	3.27	0.79	2.08	2.05	1
NPY	4852	NM_000905	JACUAC, GGUGAU	3.18	2.56	0.4	2.05	1
SNX4	8723	NM_003794	GAAUUU, GCGACG	2.95	0.55	2.64	2.05	0
TAF1C	9013	NM_139353	UCUCUA, GAUCAC	1.33	2.43	2.37	2.04	0
MPHOSPH9	10198	NM_022782	JACAGA, GGAAAC	0.67	3.19	2.27	2.04	1
KRTHA4	3885	NM_021013	GGUAUA, CAAAAG	0.42	3.01	2.7	2.04	1
MBNL1	4154	NM_207294	UGUAA, GAAAAU	0.16	3.53	2.44	2.04	1
ATP10D	57205	NM_020453	CAAAU, GCGCAGU	2.09	1.49	2.54	2.04	0
TRAPPC6B	122553	NM_177452	UAGAUU, GCUUA	2.02	2.57	1.53	2.04	0
DF	1675	NM_001928	GGUUCU, CCAAGC	2	0.01	4.11	2.04	1
SELE	6401	NM_000450	GAAUU, GAAGAA	0.13	3.54	2.45	2.04	1
NIPSNAP1	8508	NM_003634	AGGACA, CCAGGA	-0.65	4	2.77	2.04	1
BCL10	8915	NM_003921	CAGAUC, GGGCAU	-0.61	2.46	4.27	2.04	1
CSAD	51380	NM_015989	GAUUAC, UAUCAC	3.01	0.76	2.34	2.04	1
NOLA3	55505	NM_018648	GAUCGA, CAUAAA	1.49	2.22	2.39	2.03	0
MGC39696	255193	NM_152771	UCAAAU, CGUCAA	2.12	3.18	0.79	2.03	1
MGC11308	84975	NM_032889	GAUCGA, GCAUUA	1.79	2.17	2.13	2.03	0
MGC26733	200403	NM_144992	UGGCGA, GGAGCU	0.98	2.11	3	2.03	1
KBTBD1	401265	NM_001003760	CCCUAA, GGGUGG	0.97	2.91	2.21	2.03	0
GEFT	115557	NM_133483	CGCUA, AGAAAAU	0.14	3.31	2.63	2.03	1
LRP16	28992	NM_014067	GCAAGG, UGAAGA	-2.28	4.72	3.64	2.03	2
NY-SAR-41	343099	NM_206886	UACAAA, GAUAGA	0.1	2.07	3.9	2.02	1
FLJ10349	54707	NM_018066	GAGGUU, CAUCGA	-0.22	3.98	2.31	2.02	1
SRCAP	10847	NM_006662	AGAACA, GGUCGU	-0.25	2.83	3.49	2.02	1
TNNI3	7137	NM_000363	AGUUUA, GAGAUA	2.31	0.77	2.98	2.02	0
OPRK1	4986	NM_000912	GGAUCC, GAAUA	-0.56	3.44	3.18	2.02	2
FAM3C	10447	NM_014888	AGGAAAU, UACAA	0.82	2.74	2.49	2.02	0
BTBD1	53339	NM_001011885	CUCUGU, GAGAG	1.89	2.07	2.09	2.02	0
DEFB112	245915	NM_001037498	UGAAU, UUUAGU	1.07	2.02	2.96	2.02	0
HRIHFB2122	11078	NM_138632	CUAUAC, GGAUGU	0.74	2.65	2.66	2.02	0

CBL	867	NM_005188	AUAAA, UAGCCC	0.65	2.96	2.44	2.02	0
ORC2L	4999	NM_006190	GGAGUA, GGAUA	-0.5	2.8	3.75	2.02	1
PPP2R1A	5518	NM_014225	UGUGCA, GUUCA	2.04	2.26	1.74	2.01	0
MOGAT2	80168	NM_025098	CAGAUU, GAUUC	2.09	1.8	2.14	2.01	0
OR10G3	26533	NM_001005465	GGUGUU, UGUCA	2.61	2.4	1.01	2.01	0
DBP	1628	NM_001352	CGCCUU, CCAAU	2.58	2.63	0.81	2.01	0
GCHFR	2644	NM_005258	CGAUGA, CAAUAA	1.03	2.18	2.81	2.01	0
EHMT1	79813	NM_024757	UGCGAA, UGUUA	1.11	2.43	2.47	2.00	0
NCBP1	4686	NM_002486	CAAGUA, GAAAGG	0.08	2.17	3.76	2.00	1
SPAG4	6676	NM_003116	CGAUUA, GUGAG	-0.49	3.76	2.74	2.00	1
UFM1	51569	NM_016617	AAGUUC, GAACU	-0.66	3.71	2.96	2.00	1
NOTCH2NL	388677	NM_203458	CACUUA, CCAAGA	-1.32	3.71	3.6	2.00	2
