# Supplementary figures and images for: Reovirus infection is regulated by NPC1 and endosomal cholesterol homeostasis
Source: PLoS Pathog. 2022 Mar 9;18(3):e1010322. doi: 10.1371/journal.ppat.1010322 (PMC8906592; doi:10.1371/journal.ppat.1010322)

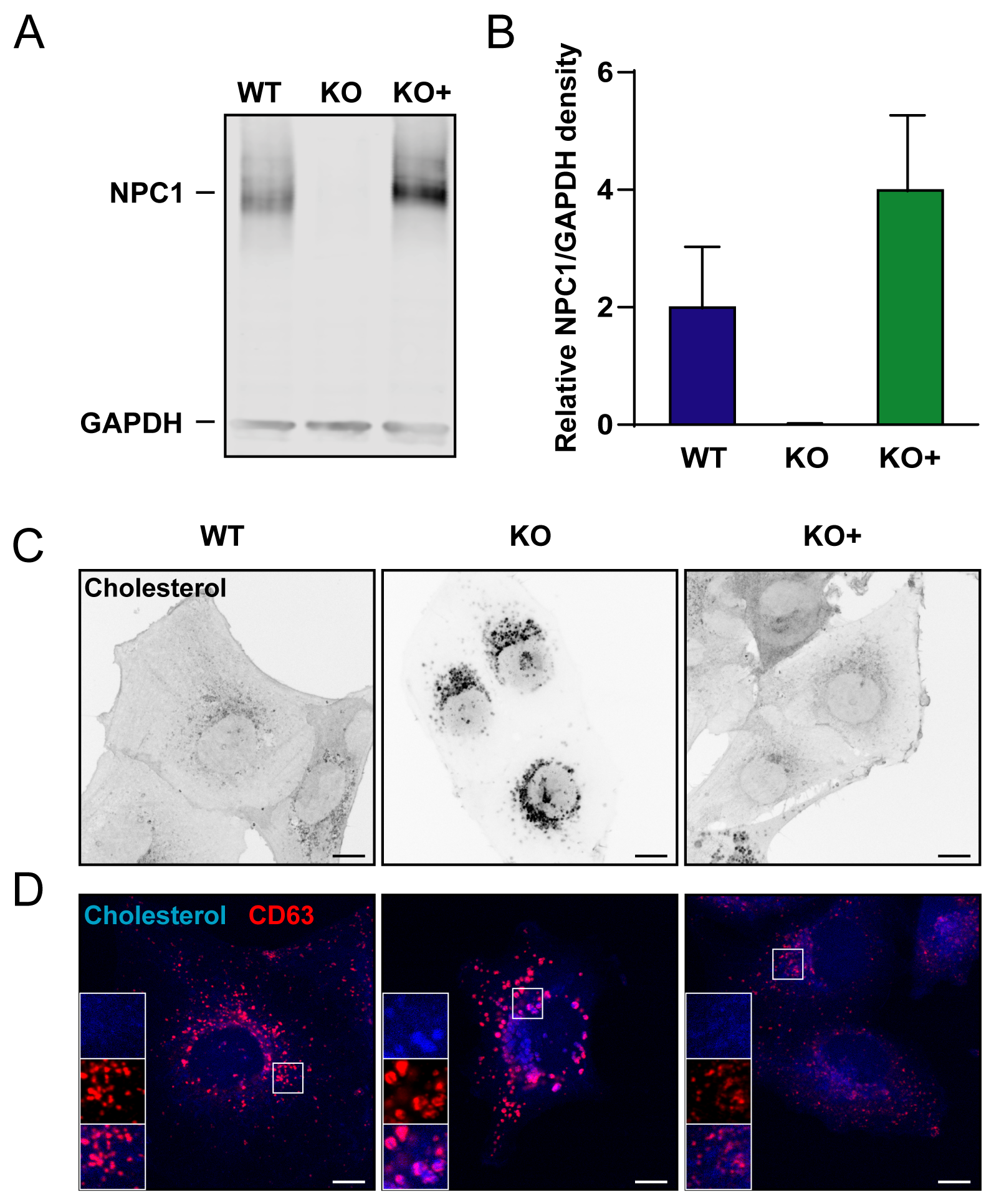

Supplement: S1 Fig — (A, B) Lysates of WT, KO, and KO+ HBMECs were subjected to electrophoresis and immunoblotting using an NPC1 antiserum. GAPDH was used as loading control. A representative immunoblot is shown. The results are presented as the mean of two independent experiments. Error bars indicate standard deviation. Statistical analysis was done by two-tailed unpaired t-test. (C) WT, KO, and KO+ HBMECs were stained with filipin III to detect cholesterol distribution. Representative images are shown. Scale bars, 10 μm. (D) WT, KO, and KO+ HBMECs were stained with filipin III and an anti-CD63 antibody to detect the subcellular localization of cholesterol. Representative images are shown. Scale bars, 10 μm. (TIF) [file ppat.1010322.s003.tif]

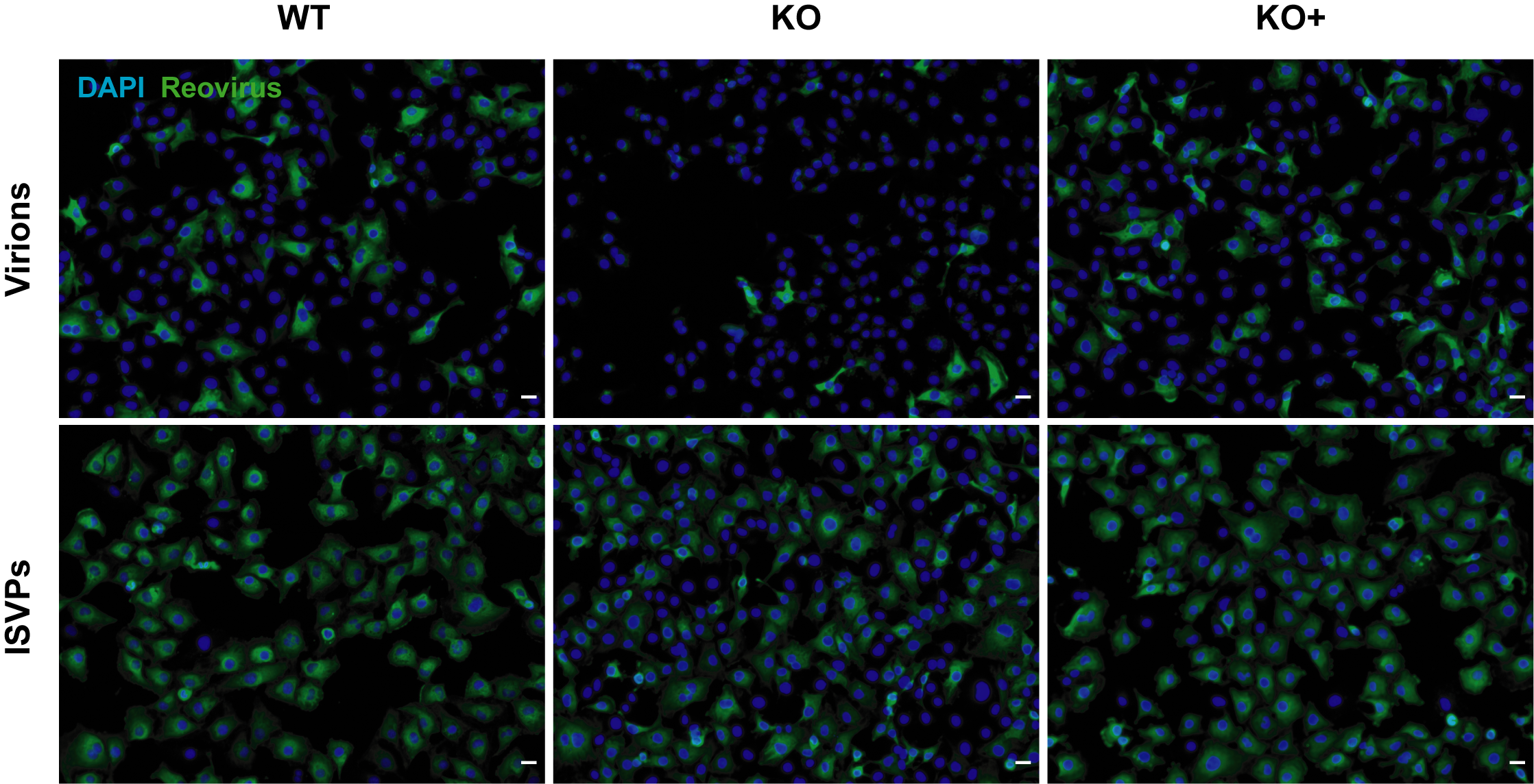

Supplement: S2 Fig — WT, KO, and KO+ HBMECs were adsorbed with reovirus (A) virions or (B) ISVPs at MOIs of 10,000 or 100 particles/cell, respectively, fixed at 18 h post-adsorption, and stained for reovirus antigen using IF. Representative micrographs are shown. (TIF) [file ppat.1010322.s004.tif]

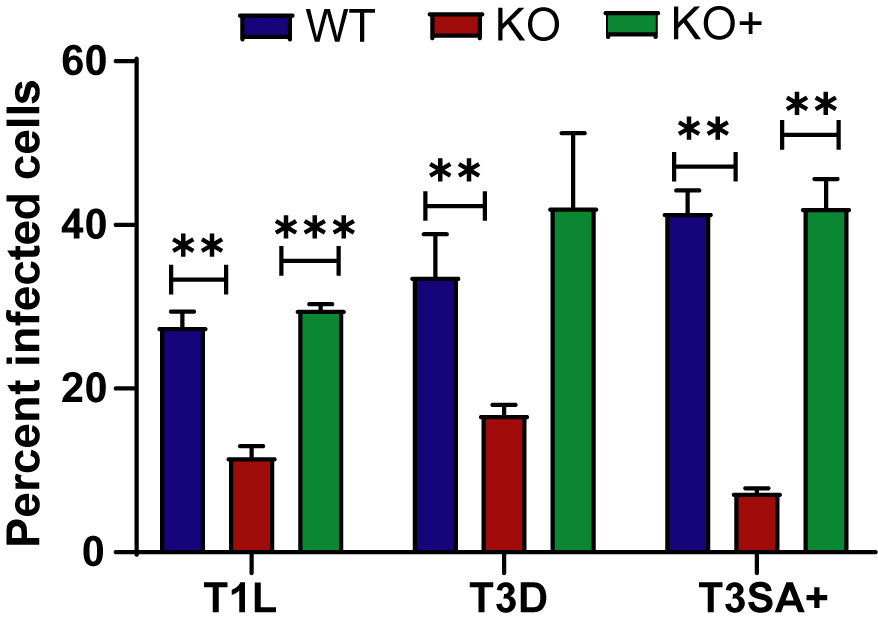

Supplement: S3 Fig — (A, B) WT, KO, and KO+ HBMECs were adsorbed with reovirus virions at MOIs of 10,000 particles/cell, and fixed at 18 h post-adsorption. The percentage of infected cells was determined by enumerating reovirus-infected cells following immunostaining with a reovirus-specific antiserum. Error bars indicated standard deviation. **, P < 0.01; ***, P < 0.001, as determine by 2-way ANOVA, Tukey’s multiple comparisons test. (TIF) [file ppat.1010322.s005.tif]

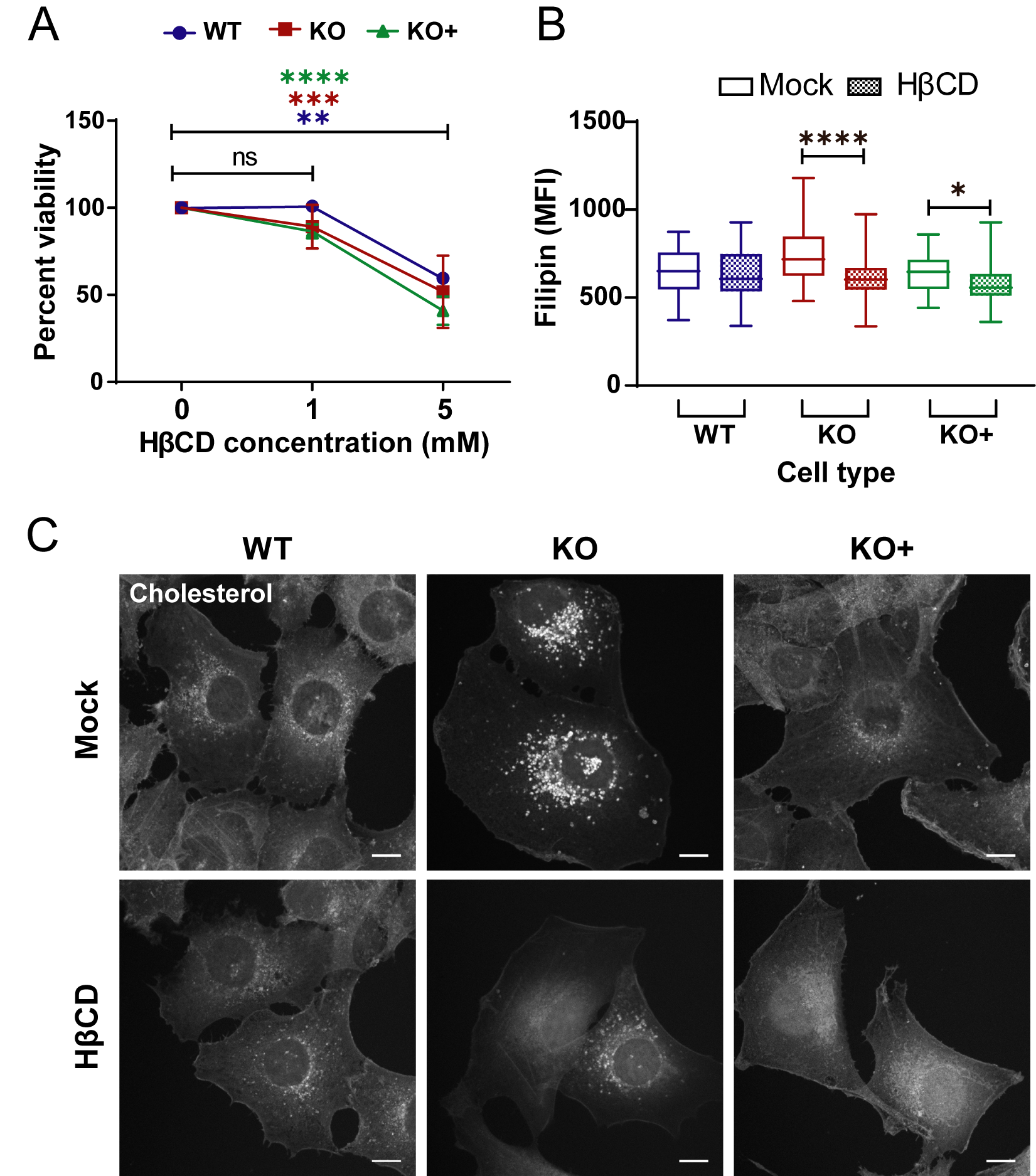

Supplement: S4 Fig — (A) WT, KO, and KO+ HBMECs were treated with HβCD at the concentrations shown for 48 h and assessed for viability using the Presto blue cell viability reagent. The results are presented as the mean cell viability of three independent experiments. Error bars indicated standard deviation. **, P < 0.01; ***, P < 0.001; ****, P < 0.0001, as determined by two-way ANOVA. (B, C) Cells were treated with 1 mM HβCD or PBS (mock) for 48 h, fixed with 4% PFA, stained with filipin III, and imaged using confocal microscopy. (B) The results are presented as the mean filipin III staining (quantified by MFI) of ~ 50 cells from three independent experiments. Error bars indicate the minimum and the maximum values. *, P < 0.05; ****, P < 0.0001, as determined by two-tailed unpaired t-test. (C) Representative images of cholesterol distribution in HβCD-treated and mock-treated cells are shown. Scale bars, 10 μm. (TIF) [file ppat.1010322.s006.tif]
